# Supplementary material for: NodoMap: A single-cell and spatial transcriptomic atlas of the mouse nodose ganglion
Source: Cell Press Blue. 2026 Jul 20;1(4):None. doi: 10.1016/j.cpblue.2026.100072 (PMC13385471; doi:10.1016/j.cpblue.2026.100072)
Supplement: Document S1. Figures S1–S15 [file mmc1.pdf]

CPBLUE, Volume *1*

Supplemental information

NodoMap: A single-cell and spatial transcriptomic atlas of the mouse nodose ganglion

Sijing Cheng, Georgina K.C. Dowsett, Kara Rainbow, Mariana Norton, Anna G. Roberts, Phyllis Phuah, Gavin A. Bewick, Brian Y.H. Lam, Giles S.H. Yeo, and Kevin G. Murphy

Supplementary Figure 1

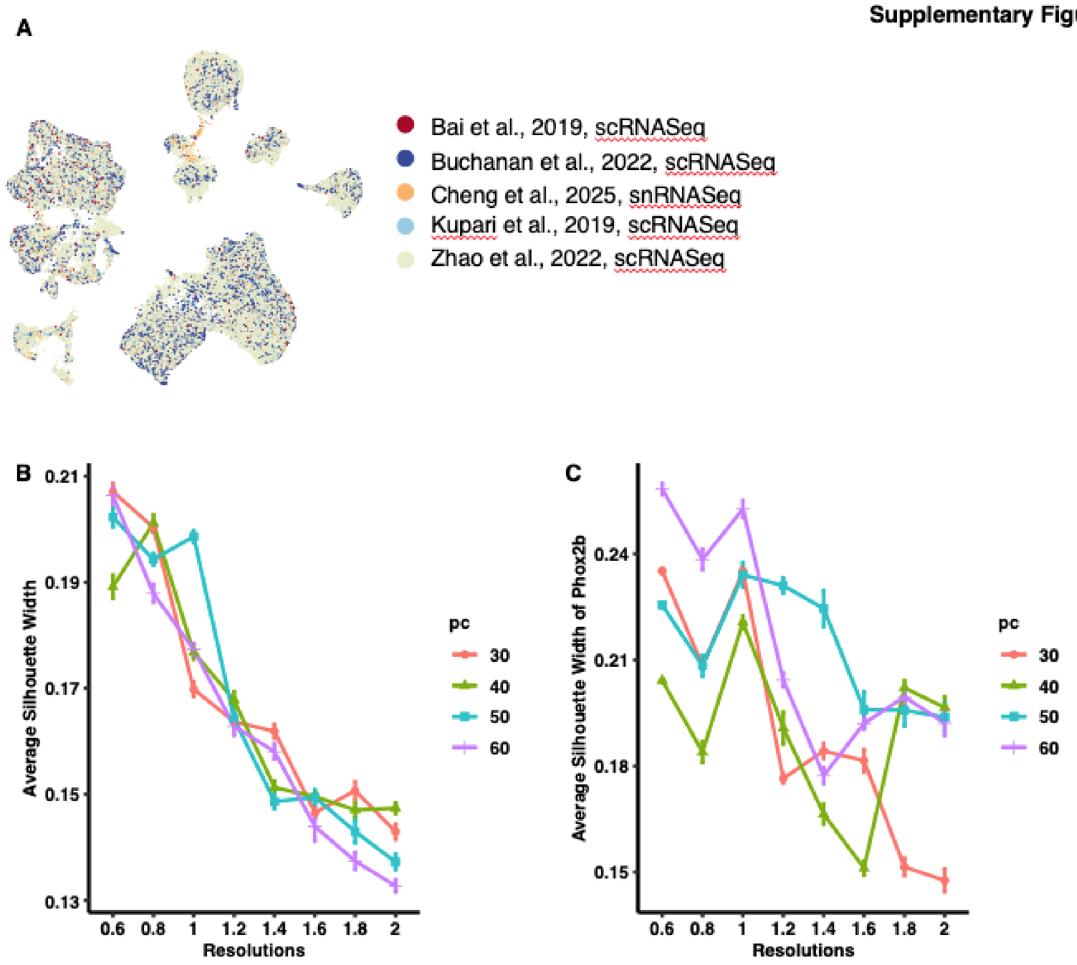

Supplementary Figure 1: Dataset integration quality control. **(A)** UMAP plot coloured by dataset. There were 1979 from Bai et al[15], 17719 from Buchanan et al[28], 1724 from Cheng et al, 4,127 from Kupari et al[14] and 80887 from Zhao et al[31] single cells/nuclei used for downstream analysis. **(B-C)** Line plots of averaged average silhouette width score of all clusters **(B)** and *Phox2b* expressed clusters **(C)** from principal components (PCs) 30 to 60 (increment = 10) and resolutions 0.6 to 2 (increment = 0.2). Together with log-normalised expression of *Phox2b* (NGN marker) and *Prdm12* (JGN marker), single cell atlas defined under PC = 50 and resolution = 1.4 was regarded as the best clustering model.

Supplementary Figure 2

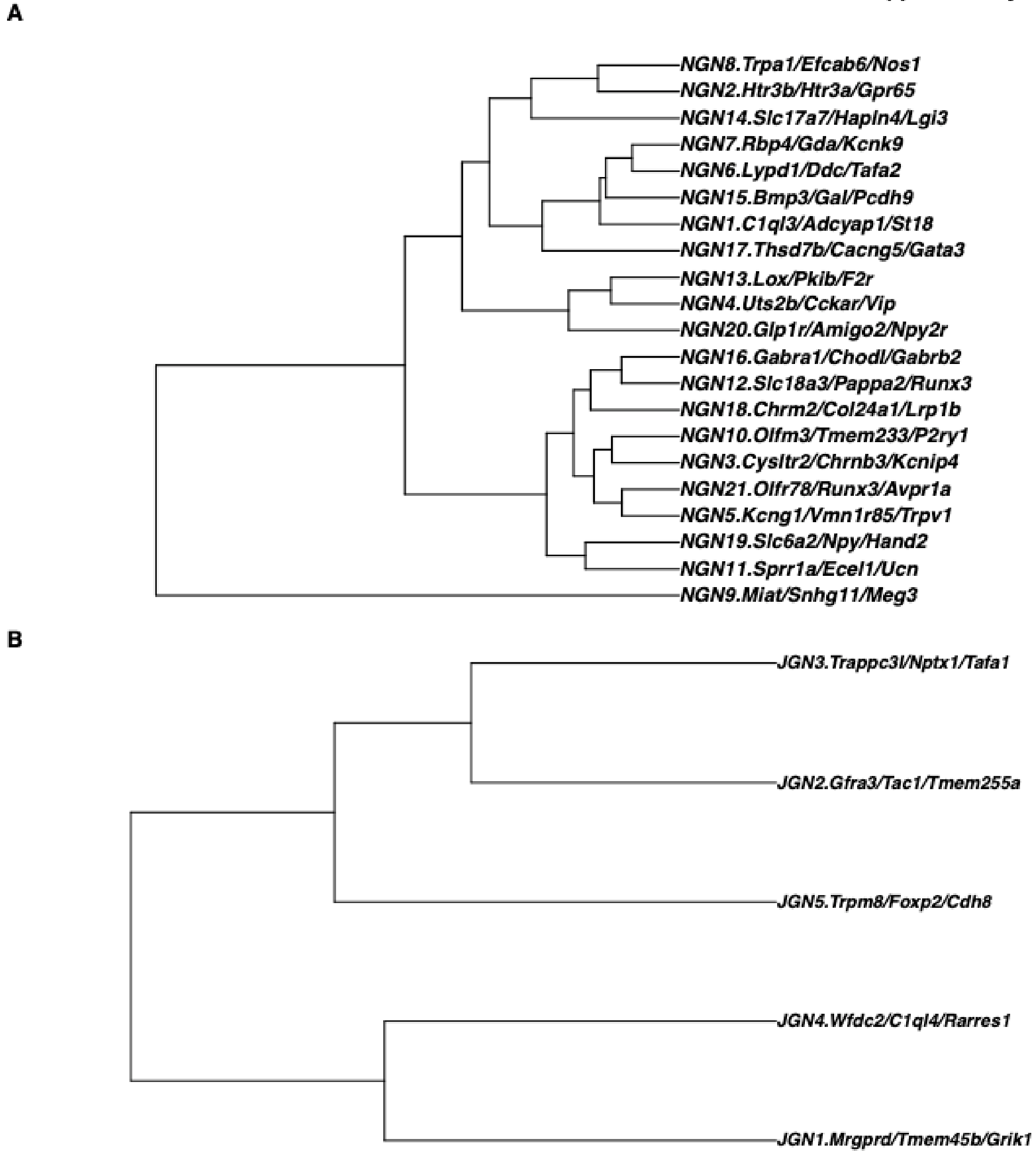

Supplementary Figure 2: Dendrograms of the neuronal clusters. **(A)** The phylogenetic tree plot of the 21 NGN clusters. **(B)** The phylogenetic tree plot of the 5 JGN clusters.

Supplementary Figure 3

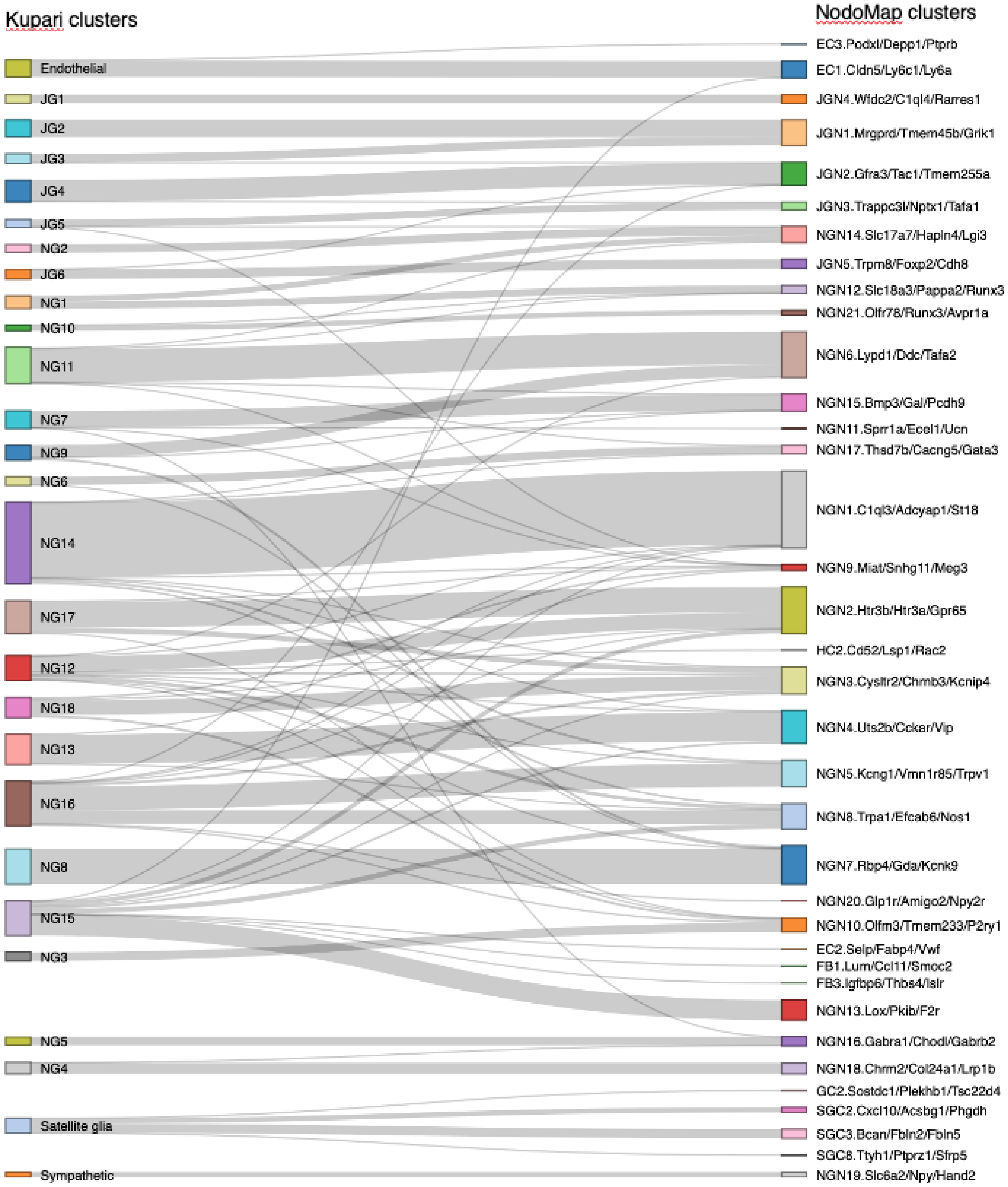

Supplementary Figure 3: Sankey plot highlighting the alignment of the cluster annotation from Kupari et al[14] compared with the annotation based on all five datasets (NodoMap clusters).

Supplementary Figure 4

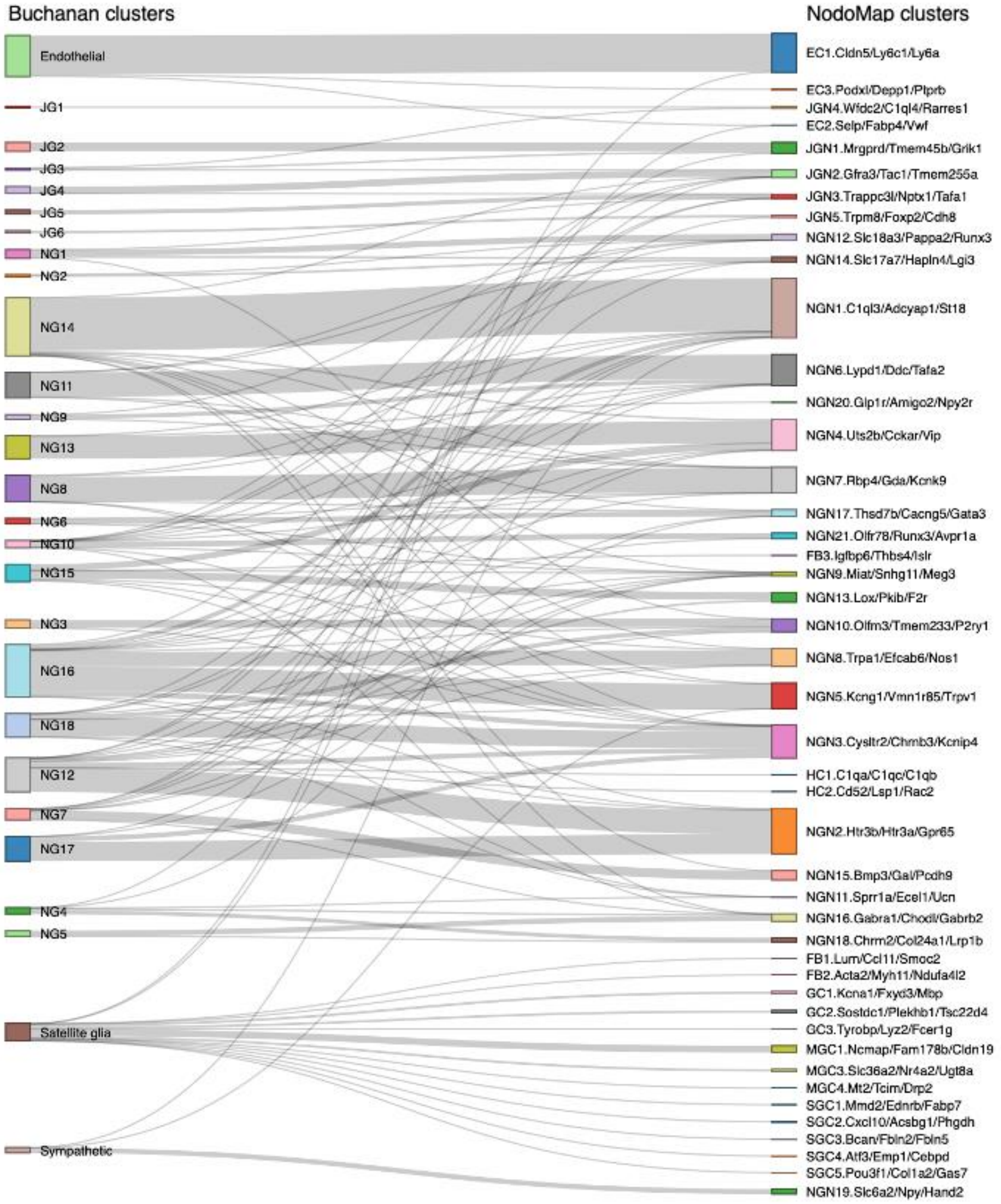

Supplementary Figure 4: Sankey plot highlighting the alignment of the cluster annotation from Buchanan et al[28] compared with the annotation based on all five datasets (NodoMap clusters).

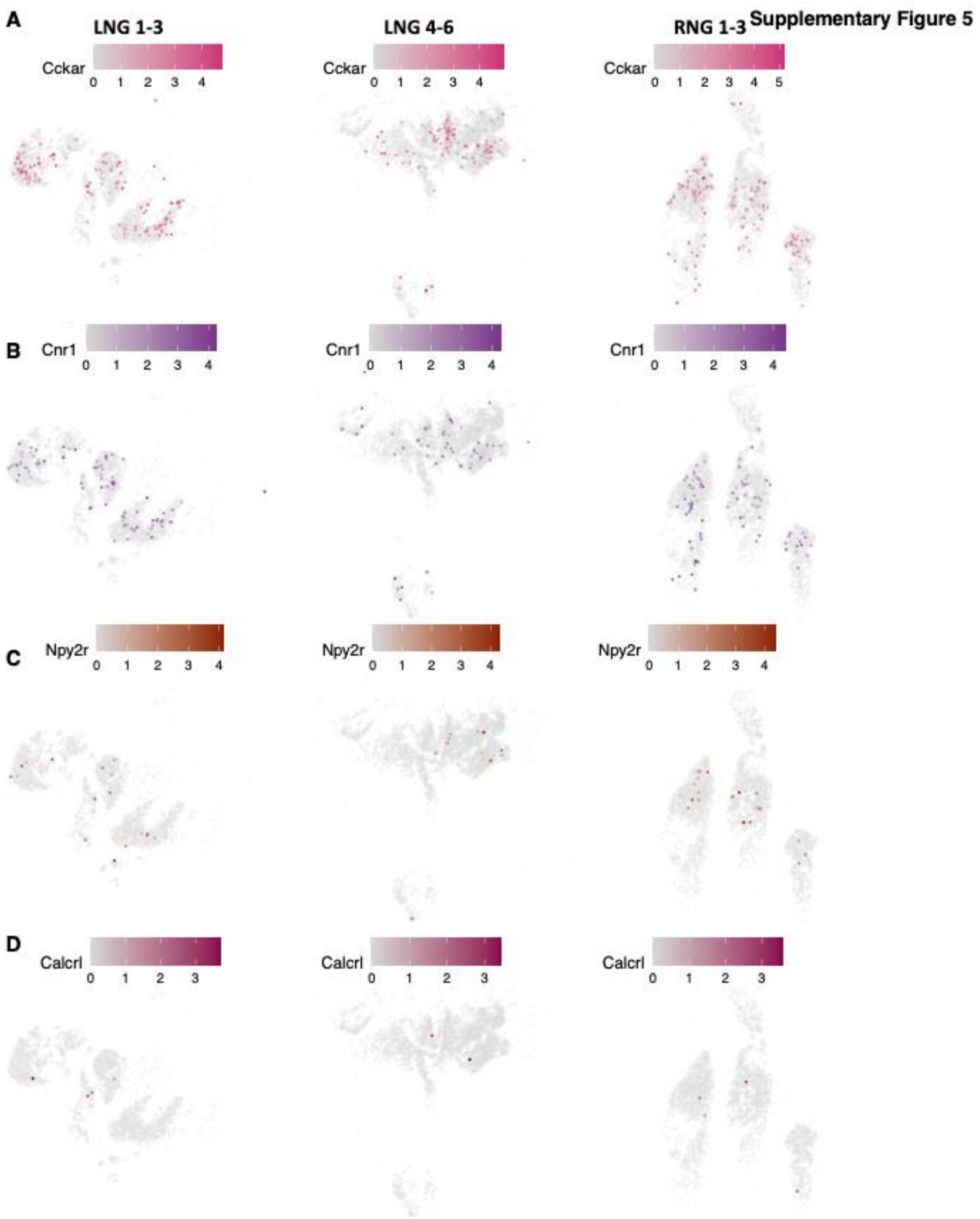

Supplementary Figure 5: Spatial feature plot showing the expression of highly expressed receptors for metabolic signals **(A-D)**. The left and central tiles each were mounted with sections from three left nodose ganglia (six individual left ganglia represented in total). The right tile was a section from three right nodose ganglia (three individual right ganglia represented).

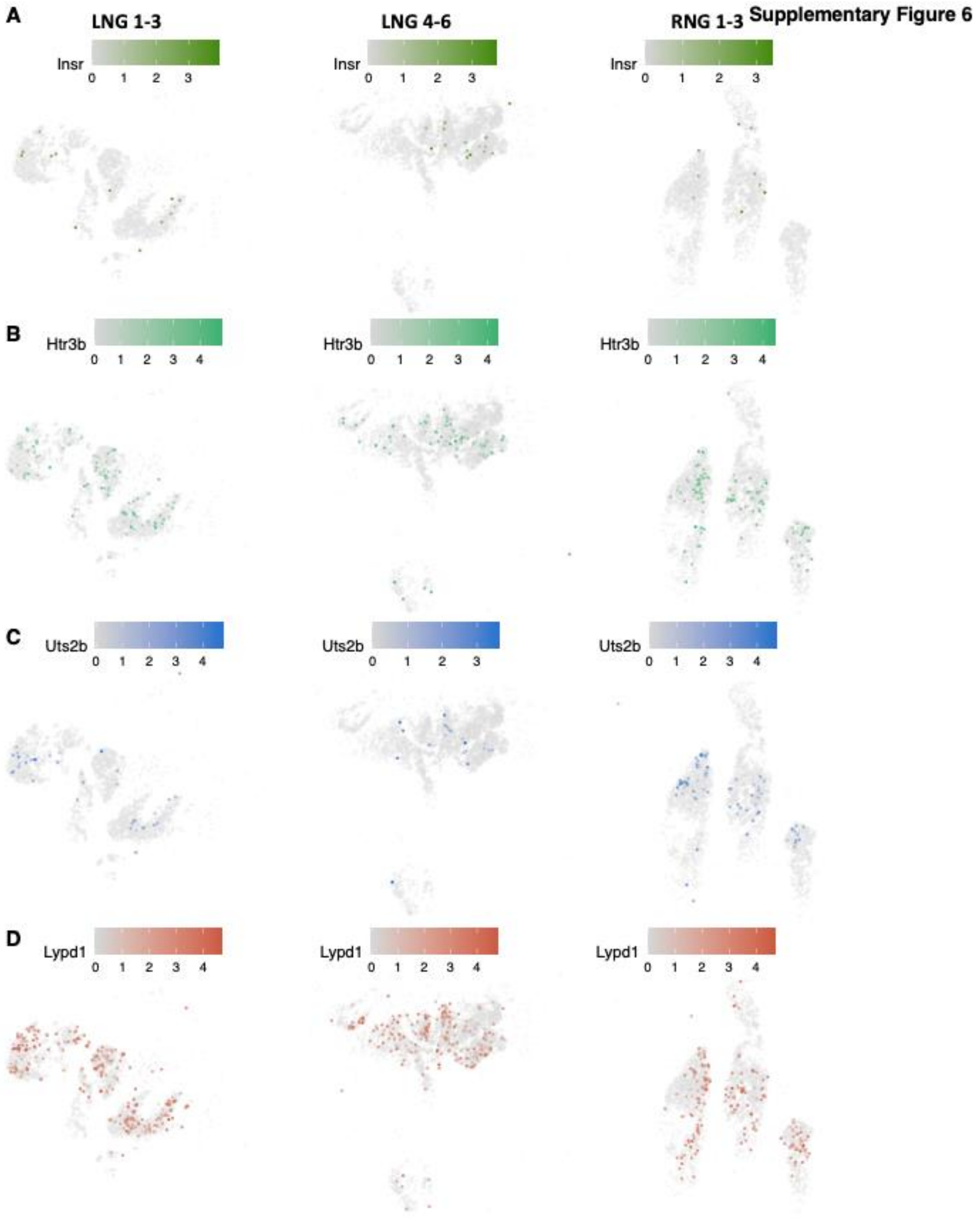

Supplementary Figure 6: Spatial feature plot showing the expression of highly expressed receptors for common NGN cluster markers **(A-D)**. The left and central tiles each were mounted with sections from three left nodose ganglia (six individual left ganglia represented in total). The right tile was a section from three right nodose ganglia (three individual right ganglia represented).

Supplementary Figure 7

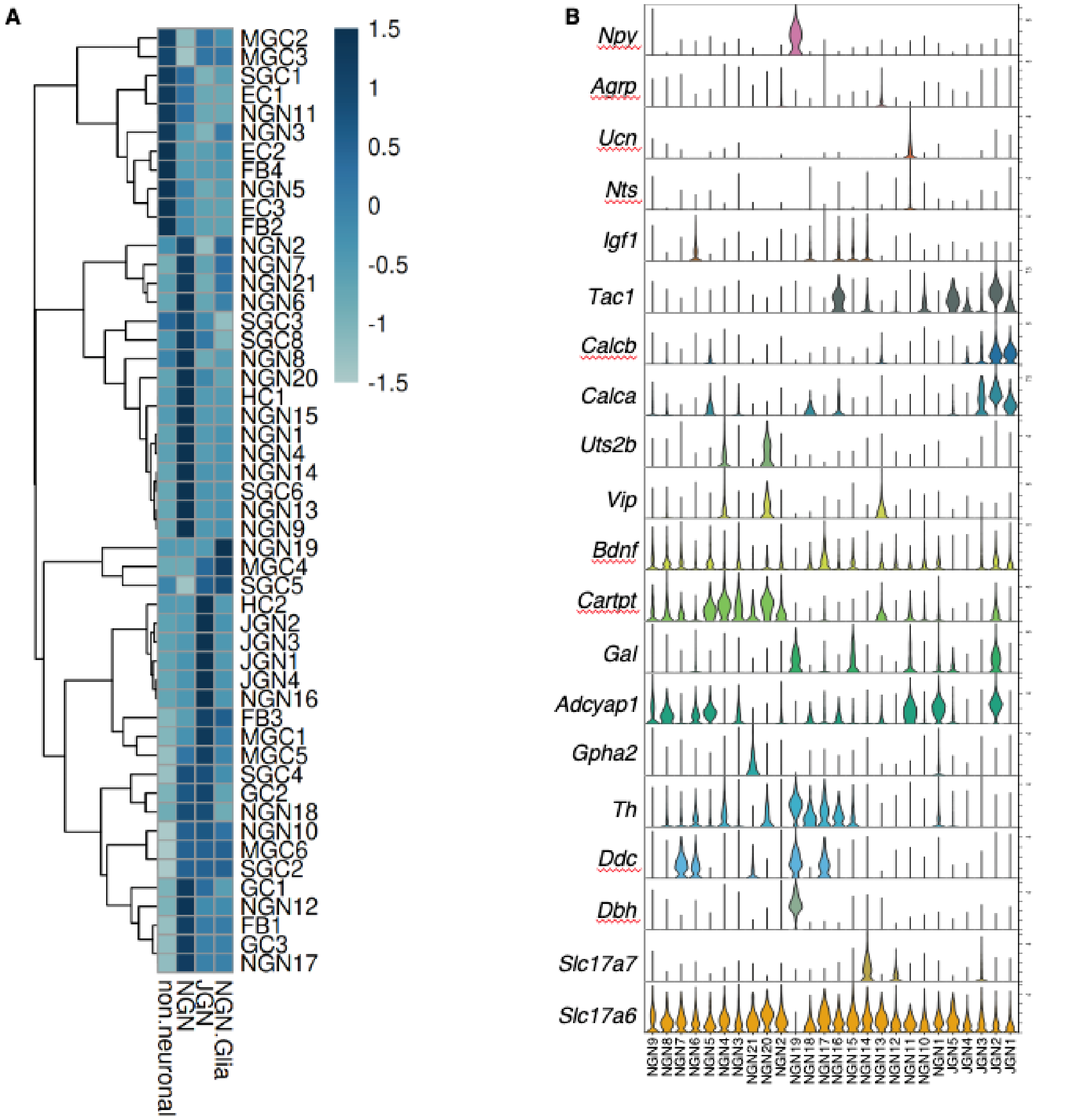

Supplementary Figure 7: Neighbourhood analysis and neuropeptide and neurotransmitter expression across neuronal clusters **(A)** Neighbourhood analysis was performed on the RCTD cell assignments in the spatial transcriptomics data. This formed a total of 4 neighbourhoods which were then labelled based on the co-occurrence of different cell types. The heatmap displays the scaled appearance of assigned cell types within each bin in the spatial transcriptomics dataset. Rows are clustered. **(B)** Violin plot showing expression of neuropeptide- and neurotransmitter-associated genes in nodose and jugular neuronal clusters.

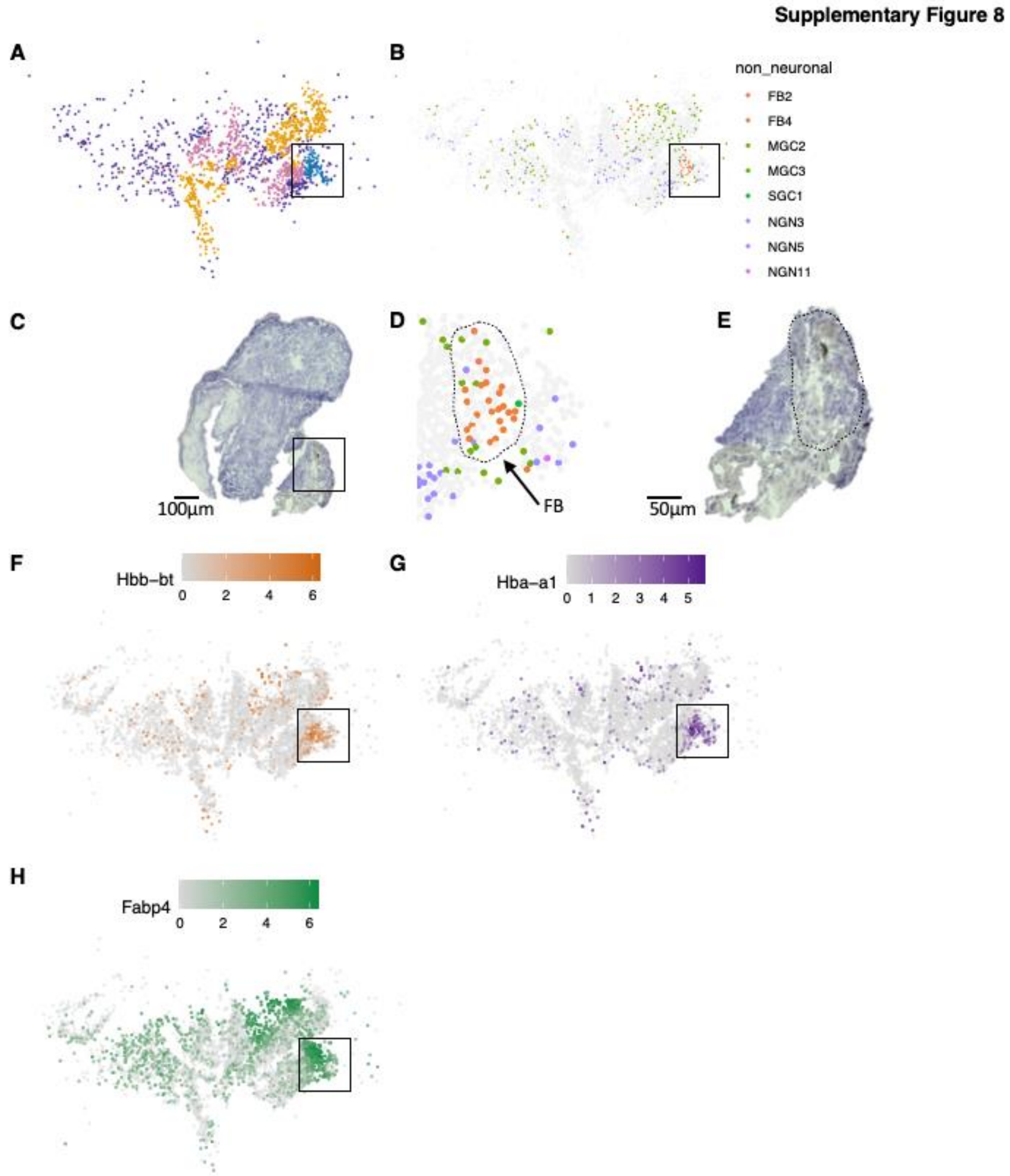

Supplementary Figure 8: Neighbourhood analyses including the non-neuronal niche which likely includes a blood vessel **(A)** Neighbourhood analysis on the RCTD output, with blue to highlight non-neuronal cell types (same as Figure 1E). **(B)** Spatial transcriptomics with RCTD and neighbourhood predicted non-neuronal clusters. **(C)** Haematoxylin staining of an adjacent section of the nodose ganglion. The zoomed-in image of the square section from panel B **(D)**, and panel C **(E)** with the fibroblasts (FB, orange) circled. **(F-H)** Spatial feature plot showing the blood cell marker genes Hbb-bt **(F)** and Hba-a1 **(G)** and the fibroblast marker gene Fabp4 **(H)**.

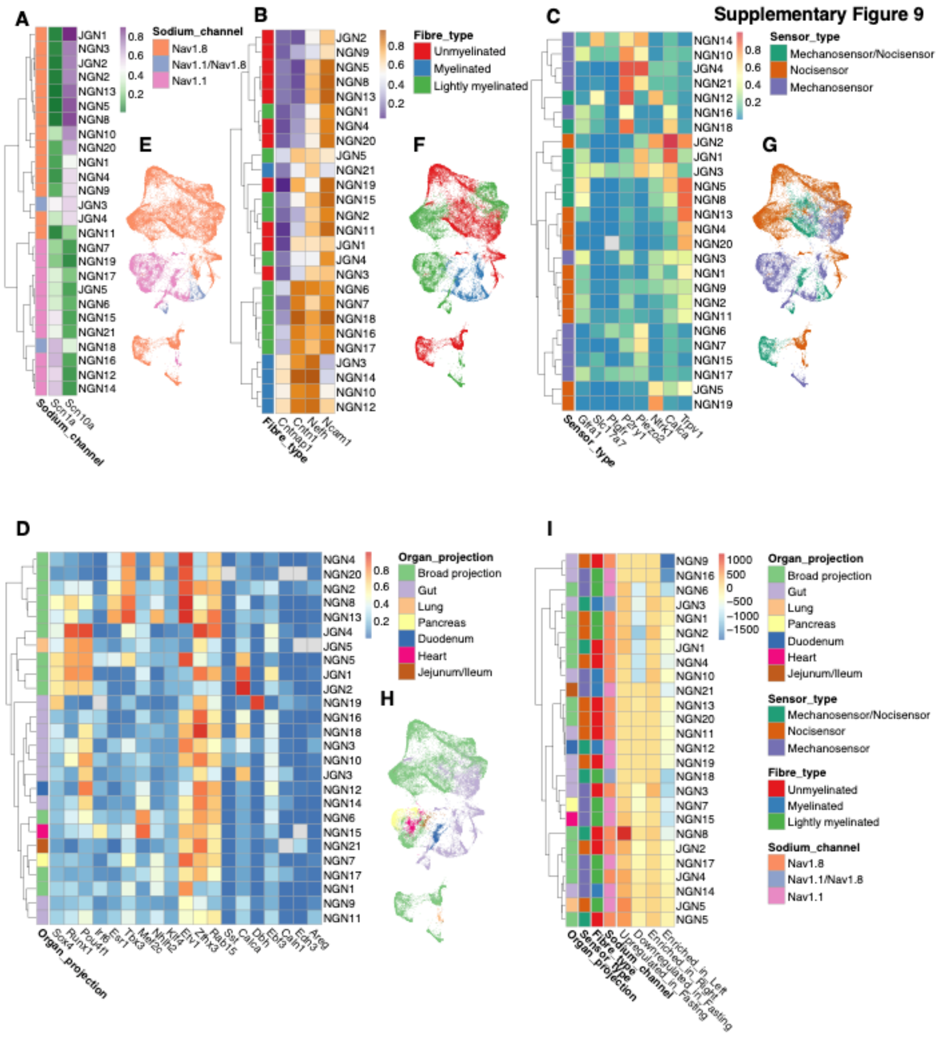

Supplementary Figure 9: Neuronal cluster classifications. **(A-D)** The clustered heatmaps showing the proportion of cells expressing the marker genes within the corresponding neuronal clusters. The neuronal clusters were clustered by the Boolean values. By comparing the expression level of marker genes, the neuronal clusters were further characterised based on their **(A)** sodium channels (Nav1.1 (*Scn1a*) or Nav1.8 (*Scn10a*)); **(B)** fibre types (myelinated or unmyelinated (*Cntnap1*, *Cntn1, Nefh, Ncam1*)); **(C)** sensor types (mechanosensor (*Gfra1, Slc17a7, Ptgfr, P2ry1, Piezo2*) or nocisensor (*Ntrk1, Calca, Trpv1*)); **(D)** organ projections (lung (*Sox4, Runx1, Pou4f1*), heart (*Irf6, Esr1, Tbx3, Mef2c*), pancreas (*Nhlh2, Klf4*), gut (*Etv1, Zfhx3, Rab15*), stomach (*Sst, Calca*), duodenum (*Dbh, Ebf3*), jejunum/ileum (*Caln1, Edn3*), broad projection (marker genes from two or more organs). **(E-H)** UMAP plots of NodoMap neuronal clusters, coloured based on the classification of sodium channels **(E)**, fibre types **(F)**, sensor types **(G)**, and the peripheral organ projections **(H)**. **(I)** The heatmap of 4 neuronal annotations and the number of upregulated/downregulated DEGs in fasting or enriched DEGs in left or right nodose ganglia.

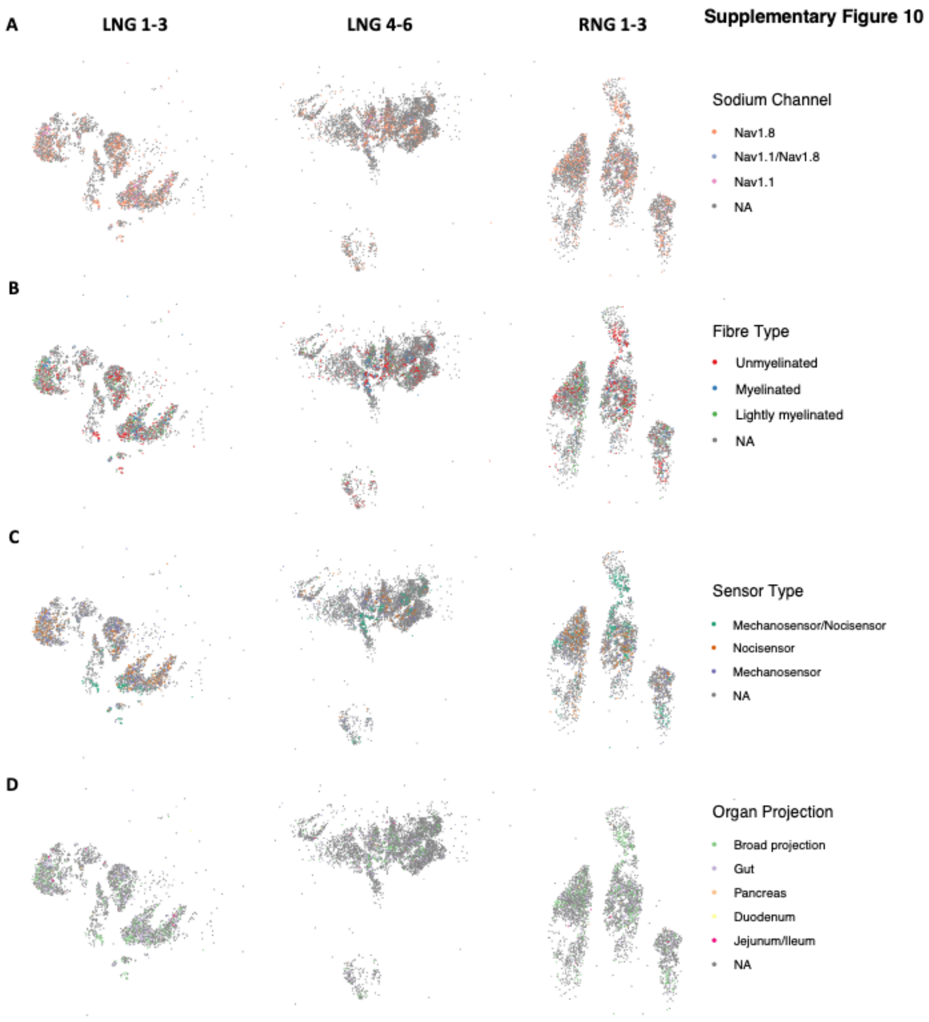

Supplementary Figure 10: Spatial analysis of neuronal cluster classifications **(A)** RCTD annotation of spatial transcriptomics spots, coloured by sodium channel type. **(B)** RCTD annotation of spatial transcriptomics spots, coloured by fibre type. **(C)** RCTD annotation of spatial transcriptomics spots, coloured by sensor type. **(D)** RCTD annotation of spatial transcriptomics spots, coloured by organ projection annotation. The left and central tiles each were mounted with sections from three left nodose ganglia (six individual left ganglia represented in total). The right tile was a section from three right nodose ganglia (three individual right ganglia represented).

Supplementary Figure 11

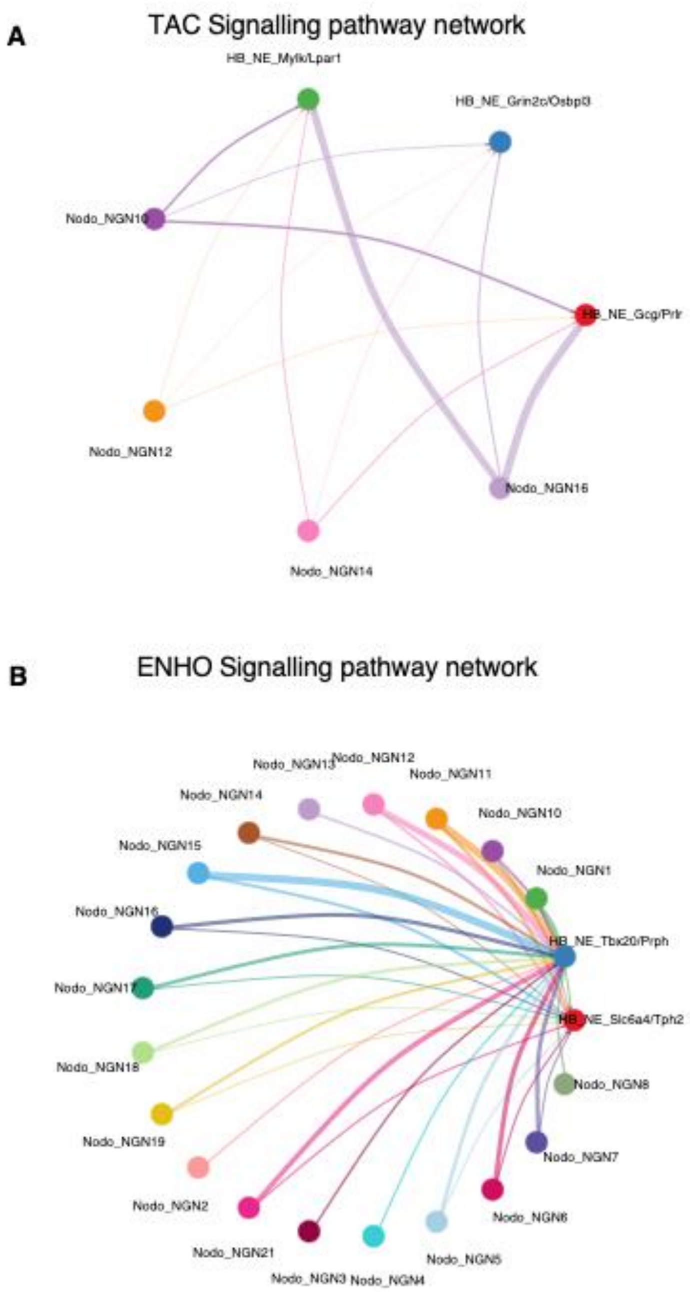

Supplementary Figure 11: CellChat mapping showing potential ligand-receptor interactions between nodose neuronal clusters and hindbrain neuronal clusters for **(A)** TAC signalling and **(B)** ENHO signalling. The thickness of the line represents the relative estimated strength of the signalling.

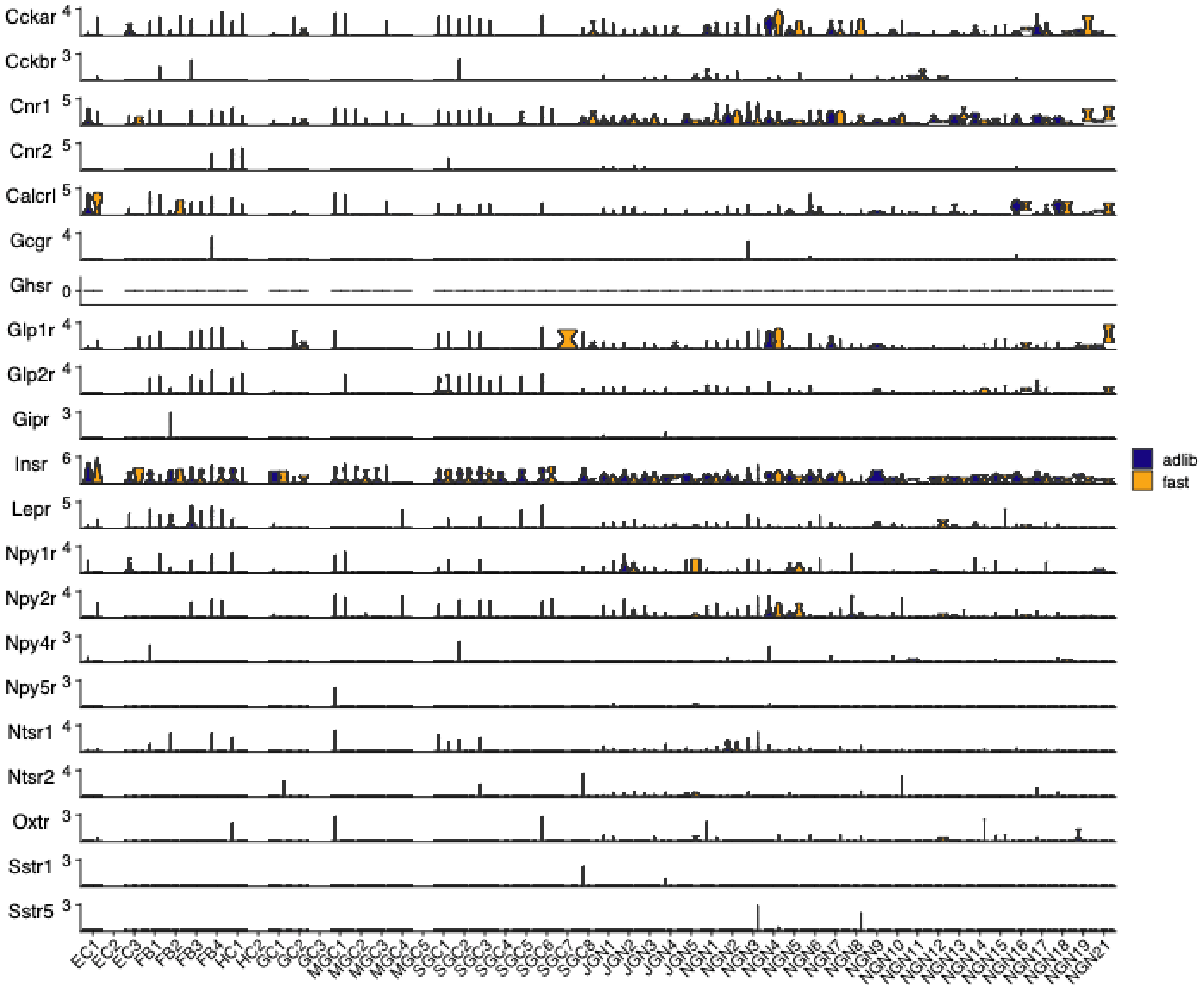

Supplementary Figure 12: Violin plot showing the expression of gastrointestinal and pancreatic hormone receptors in snRNASeq clusters compared between overnight fasting and *ad libitum* feeding**.**

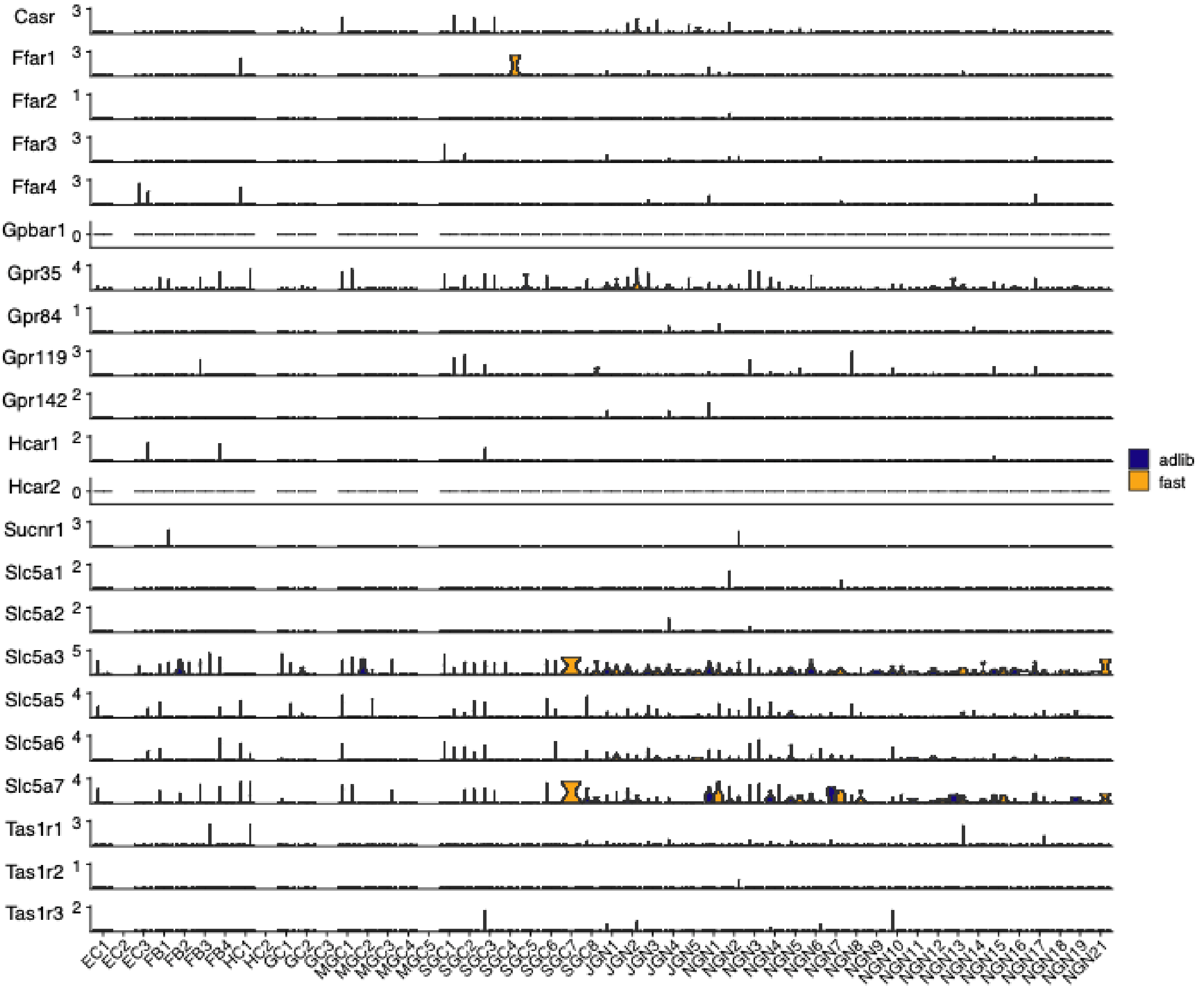

Supplementary Figure 13: Violin plot showing the expression of nutrient sensing transporters and receptors in snRNASeq clusters compared between overnight fasting and *ad libitum* feeding**.**

Supplementary Figure 14

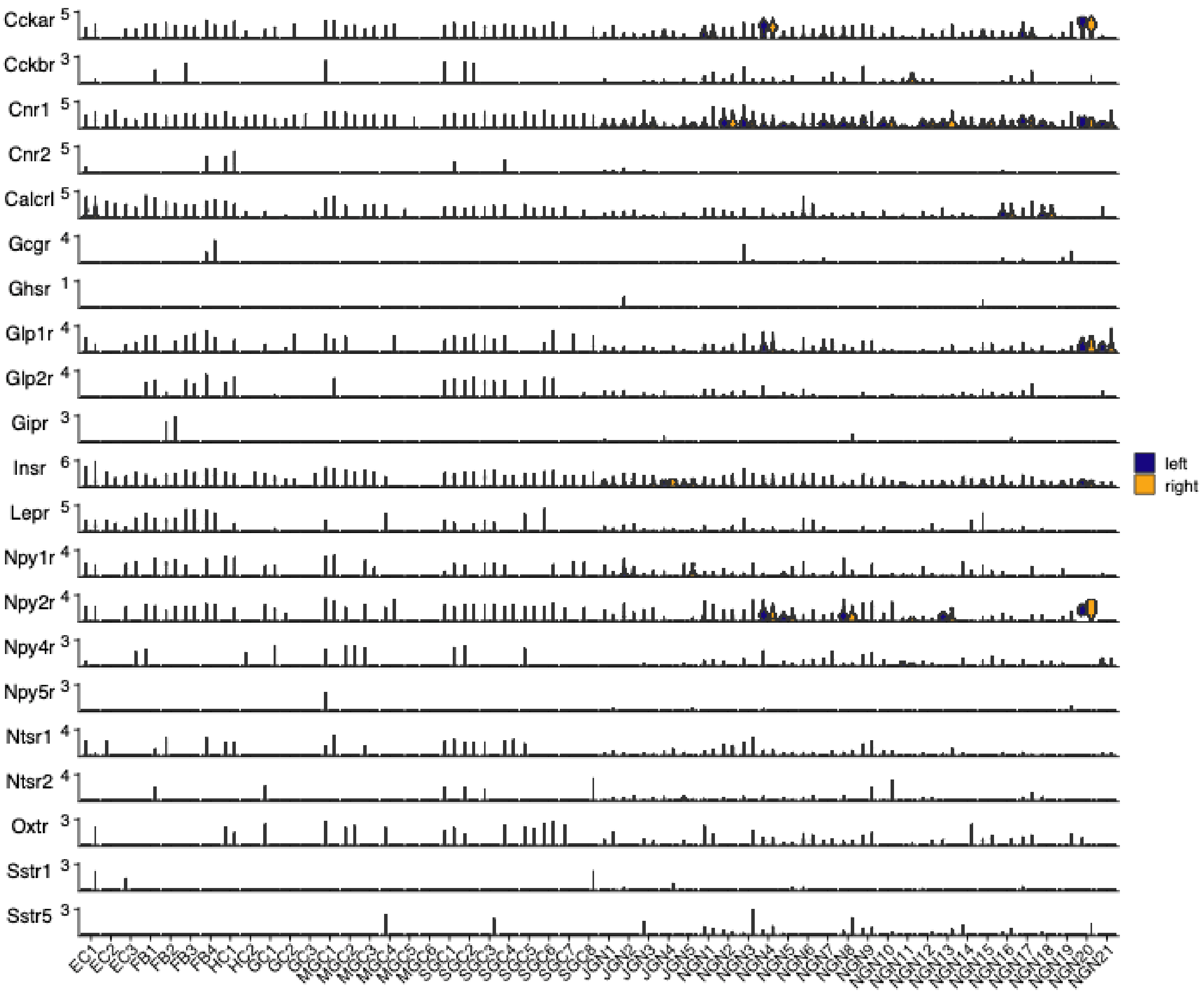

Supplementary Figure 14: Violin plot showing the expression of gastrointestinal and pancreatic hormone receptors in snRNASeq clusters compared between left and right nodose ganglia.

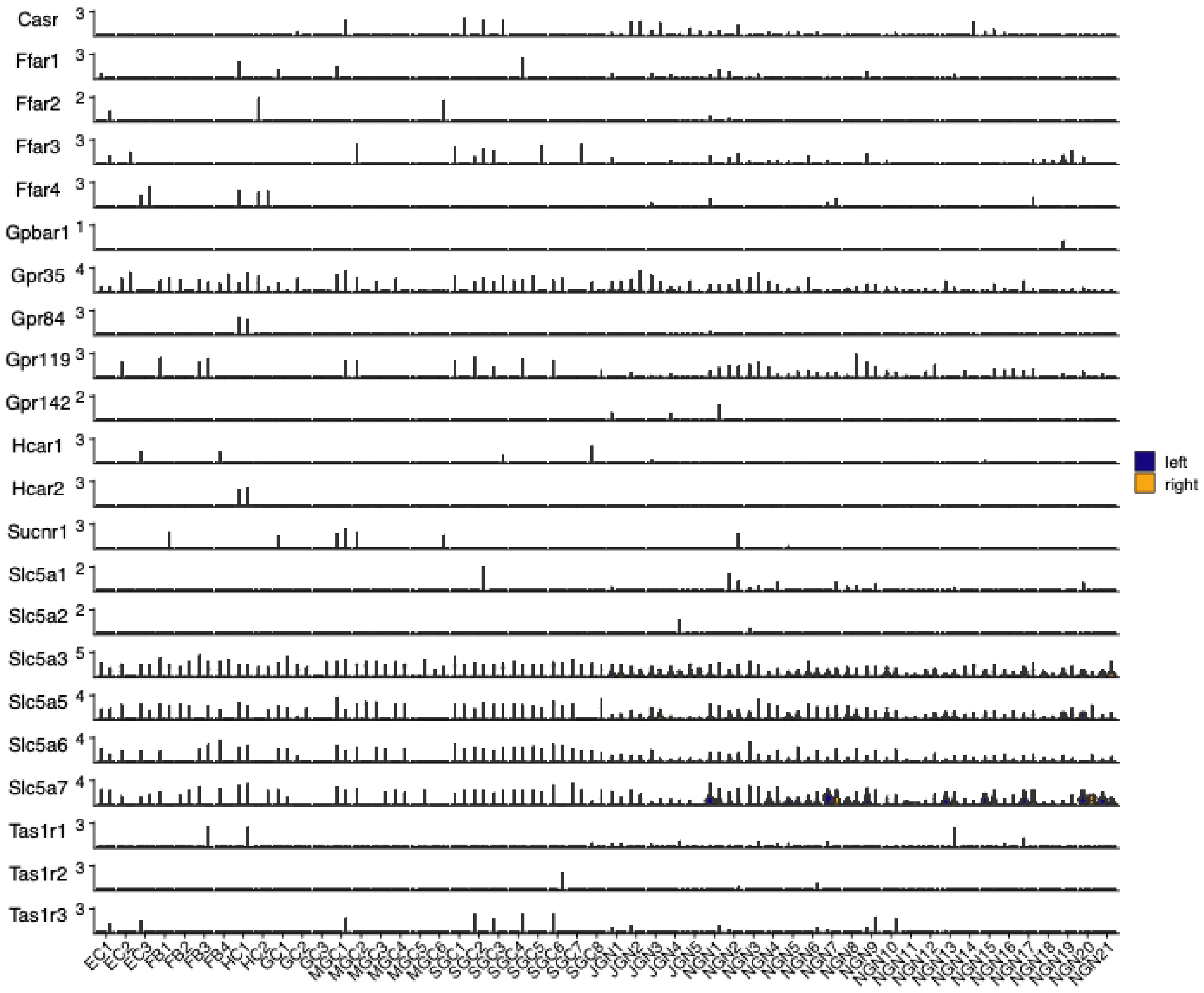

Supplementary Figure 15: Violin plot showing the expression of nutrient sensing transporters and receptors in snRNASeq clusters compared between left and right nodose ganglia.
