# Supplementary material for: NodoMap: A single-cell and spatial transcriptomic atlas of the mouse nodose ganglion
Source: Cell Press Blue. 2026 Jul 20;1(4):None. doi: 10.1016/j.cpblue.2026.100072 (PMC13385471; doi:10.1016/j.cpblue.2026.100072)
Supplement: Document S2. Article plus supplemental information [file mmc21.pdf]

Cell Press Blue

Resource

NodoMap: A single-cell and spatial transcriptomic atlas of the mouse nodose ganglion

Graphical abstract

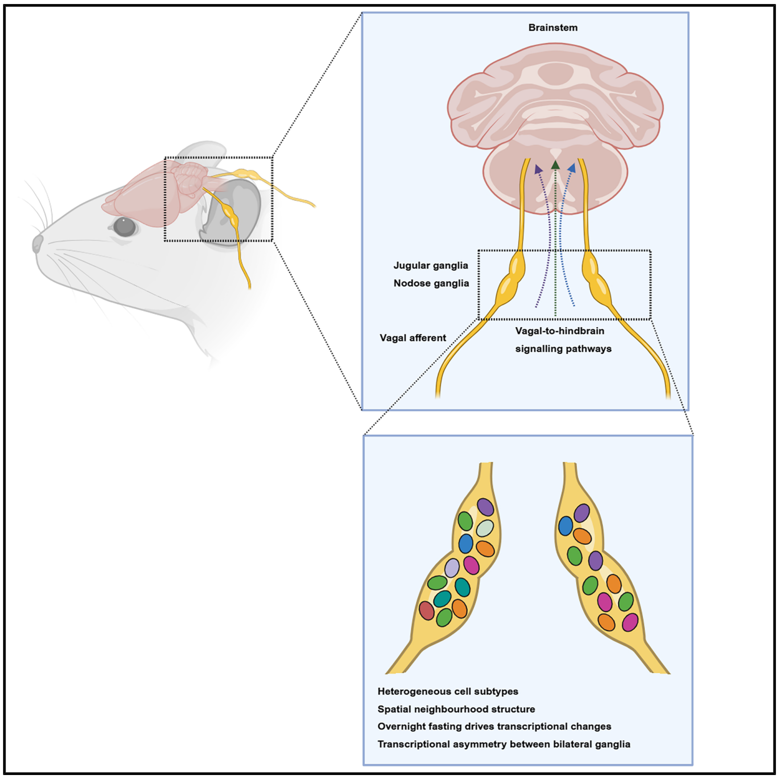

Highlights

- NodoMap integrates data from 106,436 cells and nuclei
- Different cell subtypes form spatial neighborhoods within the ganglia
- Fasting drives distinct transcriptional changes in specific cell subtypes
- Transcriptional asymmetry in specific cell subtypes between left and right ganglia

Authors

Sijing Cheng, Georgina K.C. Dowsett, Kara Rainbow, ..., Brian Y.H. Lam, Giles S.H. Yeo, Kevin G. Murphy

Correspondence

yhbl2@medschl.cam.ac.uk (B.Y.H.L.), gshy2@cam.ac.uk (G.S.H.Y.), k.g.murphy@imperial.ac.uk (K.G.M.)

In brief

Cheng and colleagues present NodoMap, an atlas of the mouse nodose ganglia, which house the neurons of the vagus nerve. Integrating single-cell sequencing and spatial transcriptomics, they define neuronal subtypes, map fasting-induced transcriptional responses, and reveal molecular asymmetries between left and right ganglia, providing a new resource for studying gut-brain communication.

Cheng et al., 2026, Cell Press Blue *1*, 100072
July 20, 2026 © 2026 The Author(s). Published by Elsevier Inc.
https://doi.org/10.1016/j.cpblue.2026.100072

CellPress

Cell Press
OPEN ACCESS

Resource

NodoMap: A single-cell and spatial transcriptomic atlas of the mouse nodose ganglion

Sijing Cheng,[1,4] Georgina K.C. Dowsett,[2,4] Kara Rainbow,[2] Mariana Norton,[1] Anna G. Roberts,[1] Phyllis Phuah,[1] Gavin A. Bewick,[3] Brian Y.H. Lam,[2,*] Giles S.H. Yeo,[2,5,*] and Kevin G. Murphy[1,5,6,*]
[1]Division of Diabetes, Endocrinology and Metabolism, Department of Metabolism, Digestion, and Reproduction, Faculty of Medicine, Hammersmith Hospital, 6th Floor Commonwealth Building, London W12 0NN, UK
[2]MRC Metabolic Diseases Unit, Institute of Metabolic Science Metabolic Research Laboratories, University of Cambridge, Cambridge CB2 0QQ, UK
[3]Diabetes and Obesity Theme, School of Cardiovascular and Metabolic Medicine and Sciences, Faculty of Life Sciences and Medicine, King's College London, London SE1 1UL, UK
[4]These authors contributed equally
[5]These authors contributed equally
[6]Lead contact
*Correspondence: yhbl2@medschl.cam.ac.uk (B.Y.H.L.), gshy2@cam.ac.uk (G.S.H.Y.), k.g.murphy@imperial.ac.uk (K.G.M.)
https://doi.org/10.1016/j.cpblue.2026.100072

SUMMARY

The vagus nerve is a central component of the parasympathetic nervous system, innervating multiple abdominal organs to monitor and regulate their function. Vagal sensory neuron cell bodies reside in the nodose ganglia, but their molecular diversity and spatial organization remain incompletely defined. Here, we generated and integrated single-nucleus RNA sequencing (snRNA-seq) data with multiple published datasets to construct a unified atlas of 106,436 cells and combined this with spatial transcriptomics to create a single-cell and spatial map of the mouse nodose ganglion (NodoMap). We identify 21 neuronal subtypes and multiple non-neuronal populations, revealing subtype-specific gene expression differences between left and right ganglia and transcriptional responses to fasting. Spatial analysis shows intermingled neuronal populations with distinct cellular neighborhoods. Together, NodoMap provides a high-resolution resource for dissecting vagal sensory circuits and identifying molecular targets involved in energy homeostasis and metabolic disease.

INTRODUCTION

The vagus nerve is a major component of the parasympathetic nervous system and transmits information crucial to an array of physiological functions between the brain and peripheral organs. Vagal afferent neurons signal to the brainstem, where they can act on interneurons that either modulate other circuits or input into local vagal efferent neurons, forming vagovagal neurocircuits that detect and respond to sensory inputs to regulate motor functions.[1,2] The vagus is an integral part, and the main neuronal pathway, of the gut-brain axis, which regulates appetite and gut function. The subdiaphragmatic sensory afferent fibers of the vagus nerve innervate the gastrointestinal tract, where vagal mechanoreceptors and chemoreceptors can detect gastrointestinal distension, as well as the presence and absorption of nutrients, both directly and via their effects on the release of gastrointestinal hormones.[3,4] Information regarding luminal pressure, nutrient, and hormonal signaling can thus be promptly transduced to the brain and integrated to regulate appetite and gastrointestinal motility. Vagus nerve stimulation can also be used to treat specific neurological conditions[5–8] and has been suggested to be potentially useful in other diseases, including obesity.[9–12] Understanding which specific vagal neuron populations respond to metabolic hormones such as glucagon-like peptide-1[13,14] and how such signaling is transmitted to the brain is also highly relevant to the development of new treatments for metabolic disease.

Beyond the gut, the vagus nerve also conveys sensory information from the heart, lungs, liver, and kidneys and plays important roles in regulating cardiovascular function, respiration, and inflammatory responses. The anti-inflammatory reflex mediated by vagal efferents has attracted growing interest as a potential therapeutic target in inflammatory diseases.[15] Vagal afferent signaling is also important in transmitting information about immune status and infection to the brain, contributing to sickness behavior and the coordination of systemic responses to pathogens.[16–19]

The breadth of physiological systems under vagal influence underscores the importance of understanding the cellular and molecular diversity of the neurons that form this nerve. The cell bodies of vagal afferent fibers reside in the nodose ganglia, located at the base of the skull.[20] In mice, the nodose ganglia form part of a nodose-jugular ganglia complex,[21] and mouse models have been widely used for preclinical studies of vagal

© 2026 The Author(s). Published by Elsevier Inc.
This is an open access article under the CC BY license (http://creativecommons.org/licenses/by/4.0/).

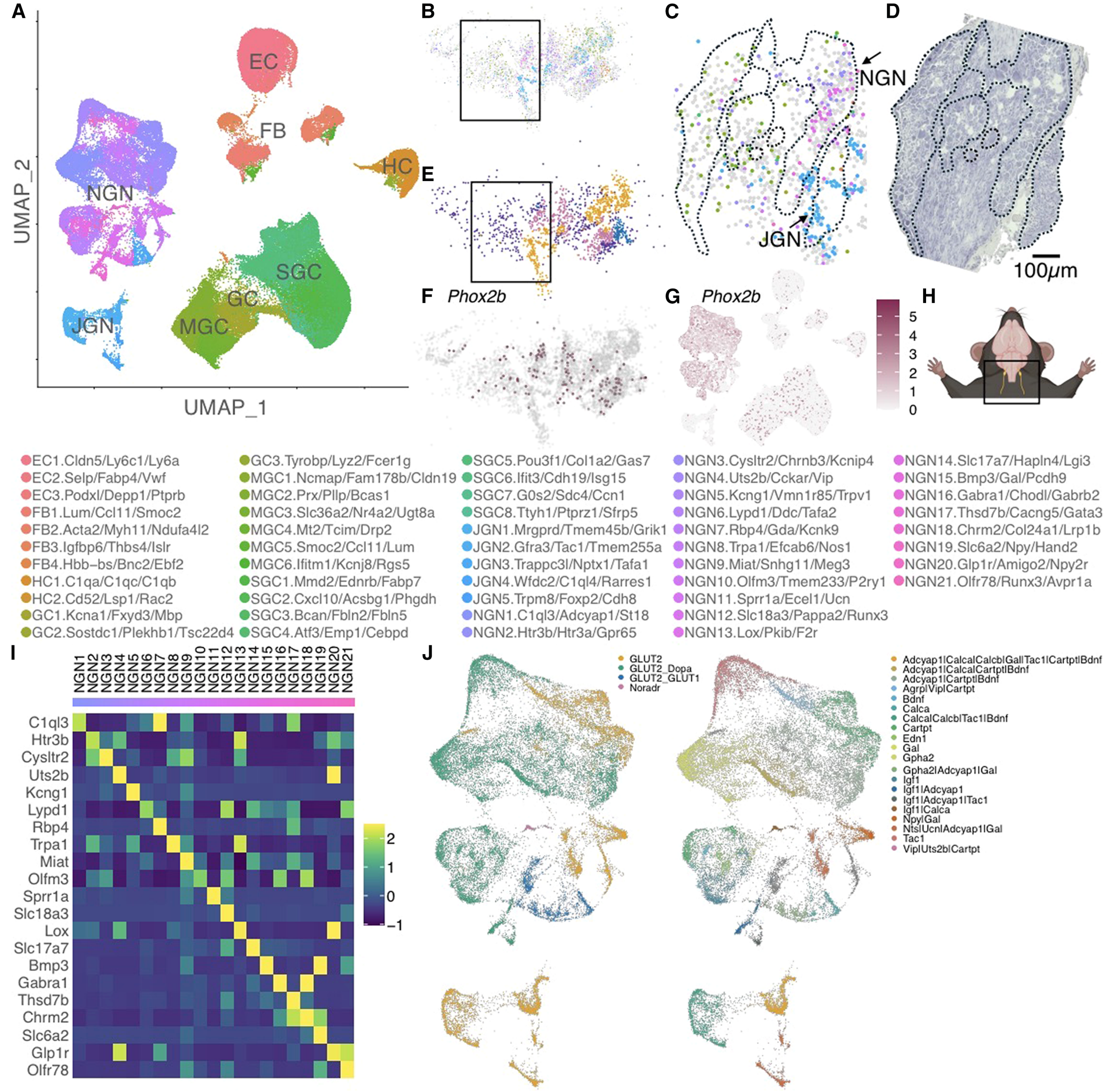

Figure 1. A spatio-cellular atlas of the mouse nodose ganglion

(A) A uniform manifold approximation and projection (UMAP) plot of the single-cell/single-nucleus sequencing (sc/snRNA-seq) dataset, colored by cluster and with cluster names written below. We integrated 4 publicly available and 1 in-house sc/snRNA-seq datasets of the mouse nodose ganglia to create a database of 106,436 cells. Of these, 26,554 were neurons, and 50,485 were satellite glial cells and myelinating glial cells. There were also 10,709 endothelial cells, 8,874 fibroblasts, and 4,963 hematopoietic cells. Clusters were annotated based on cell type and the top 3 marker genes with the highest specificity for that cluster.

(B) Spatial transcriptomics of the nodose ganglion. To identify the cell type present underneath each spot, we integrated the sc/snRNA-seq atlas together with the spatial transcriptomics data using RCTD (see STAR Methods) to predict the cell type and number of cells present at each bead. Slide-seq data are presented for 1 tile of 3 left nodose ganglia sections; spots represent beads where RNA transcripts were detected. Spots are colored based on the predicted cell type present at each bead. Beads were annotated with a cell type only if the RCTD pipeline identified them as singlets. Colors match the sc/snRNA-seq cluster colors.

(C) Zoomed-in image of the square section from (B).

(D) Hematoxylin staining of an adjacent section of the same region in (B). In (C and D), regions of the tissue containing nodose and jugular neurons have been highlighted, matching the output from RCTD. Beads were assigned nodose (purple) and jugular (blue) neuronal subtypes by RCTD.

(E) Neighborhood analysis on the RCTD output, including the cell types assigned to singlet and multiplet beads. We identified 4 distinct neighborhood types. Neighborhoods were labeled based on the cell types that co-occur in the regions. Neighborhoods highlighted in pink predominantly contained NGN cell types,

(legend continued on next page)

signaling. Application of single-cell (scRNA-seq) and single-nucleus (snRNA-seq) RNA sequencing to sensory ganglia has revealed far greater cellular heterogeneity than previously appreciated[22,23] and has enabled the linking of transcriptional identity to function. In the last decade, these new technologies have revolutionized our understanding of the vagus, revealing that nodose ganglia neurons are highly heterogeneous.[24–30] However, each of these nodose datasets was generated and analyzed in isolation, using different analytical parameters and variable cell-type resolution. No unified reference atlas integrating information across studies exists, and the transcriptional diversity of non-neuronal populations within the ganglia has received comparatively little attention. Though it has long been known that nutritional state influences vagal afferent signaling,[31–36] the transcriptional changes occurring in individual neuronal subtypes in response to fasting have not been defined. Similarly, although different signals have been shown to be transduced through the left or right nodose and studies have reported differences in gene expression between the left and right nodose ganglia,[27,37–39] the extent and nature of these lateralized differences have not been systematically characterized. Furthermore, the spatial organization of the nodose ganglia at cellular resolution has not been described, and the extent to which different neuronal populations occupy distinct regions or exist within structured multicellular neighborhoods remains unexplored.

Here, we address these gaps by generating new snRNA-seq data from the mouse nodose ganglia and integrating these with four existing published datasets to create a unified single-cell atlas of 106,436 cells, which we have named NodoMap. We complement this with high-resolution spatial transcriptomics, enabling the spatial mapping of transcriptionally defined cell types within the ganglia.

RESULTS

Creating the NodoMap

Several scRNA-seq studies have been performed on murine nodose ganglia.[24,28,30,37,40] However, single-cell dissociation has to be performed on fresh tissue, is time-consuming, risks cell-type selection bias, and often induces transcriptional perturbation.[41] In contrast, snRNA-seq can be performed on frozen samples, and nuclear RNA has been found to be more stable than cytoplasmic RNA in brain samples.[42] Crucially, several studies have shown that the transcriptomic profile of murine brain tissues generated from snRNA-seq is comparable to that generated by scRNA-seq.[41–44] Thus, we performed snRNA-seq on left and right nodose ganglia taken from mice fed *ad libitum* or fasted overnight. To establish a clear transcriptomic definition of the nodose ganglia, we integrated our in-house snRNA-seq data with four other published datasets (Figure S1A),[24,30,37,40] in order to create a unified reference atlas, NodoMap (Figure 1A). The atlas comprises a total of 106,436 cells and characterizes corresponding cell types across different studies (Tables S1, S2, S3, S4, S5, S6, S7, S8, S9, S10, S11, S12, S13, S14, S15, S16, S17, S18, and S19).[45]

In mice, the nodose and jugular ganglia merge to form the vagal ganglia.[21] Our analysis identified a total of 52 clusters, which we annotated based on cell type and three marker genes with the highest specificity score (Figures 1A and 1I; Tables S1, S2, and S3). We identified 21 nodose and 5 jugular neuronal clusters; 3 glial, 8 satellite glial, and 6 myelinating glial cell clusters; and 3 endothelial, 4 fibroblast, and 2 hemopoietic cell clusters (Figures 1A and S2; Table S4). To validate our clustering strategy with other datasets included in NodoMap, we compared our clustering with that reported by Kupari et al.[30] and Buchanan et al.[37] and found that while we had slightly higher nodose neuronal granularity, cell-type grouping was well preserved across the studies (Figures S3 and S4). We also identified more non-neuronal clusters than Kupari, highlighting improved cell-type segregation through integration of multiple datasets and higher cell count. Interestingly, the number of jugular neuronal clusters remained similar, suggesting limited heterogeneity. In addition to snRNA-seq, we performed whole-transcriptome spatial transcriptomics at a resolution of 10 μm (Curio Seeker platform[46]) on a total of 9 nodose ganglia sections from the left and right ganglia. Integration of snRNA-seq and spatial transcriptomics was performed using the robust cell-type decomposition (RCTD)[47] method, which allowed us to map the majority of the snRNA-seq clusters onto both the left and right nodose ganglia (Figures 1B–1E).[47] As expected, expression of the nodose ganglia neuronal marker *Phox2b* was able to distinguish between nodose and jugular neurons in the snRNA-seq dataset[48] and was also found in regions where nodose neuronal clusters were mapped in the spatial transcriptomics dataset (Figures 1F and 1G). We also saw some low-level *Phox2b* expression in myelinating glial cells, satellite glial cells, and other glial cell populations[48] in our snRNA-seq dataset. The spatial expression of highly expressed receptors for metabolic signals and common nodose ganglia neuron (NGN) cluster markers is shown in Figures S5 and S6.

The spatial relationships between different nodose ganglia cell types have not previously been comprehensively evaluated. We carried out cellular neighborhood analyses (see STAR Methods)[49] to investigate whether specific multicellular combinations were more likely to be found together, indicating structural organization of regions within the ganglia. We identified four types of cellular neighborhood from the spatial transcriptomics data (Figures 1E and S7). As expected given the known

NGN/glia neighborhoods are highlighted in purple, JGN neighborhoods are indicated in orange, and a non-neuronal neighborhood is highlighted in blue (see the legend of Figure S5 for more details on expression patterns in each neighborhood).
(F and G) *Phox2b* is a marker highly expressed in nodose neurons with only low expression in jugular neurons. The cells expressing *Phox2b* were colored based on the same scale bar. (F) Log-normalized expression of *Phox2b* in 1 tile of the spatial transcriptomics dataset. (G) Log-normalized expression of *Phox2b* in the single-cell atlas. *Phox2b* expression is enriched in nodose ganglion neuronal clusters.
(H) Diagram highlighting the orientation of the nodose ganglion sections in the mouse.
(I) Heatmap showing scaled expression of marker genes in nodose ganglion neuronal clusters.
(J) UMAP plots of nodose and jugular neurons, colored by neurotransmitter (left) and neuropeptide (right) assignment.

segregation of nodose and jugular neurons within the ganglia, one neighborhood consisted of the presence of both nodose neurons and glia (NGN3, MGC2, SGC5, MGC4, and NGN2 being the top 5 most significantly present in this neighborhood), another was formed of mainly nodose ganglion neurons (NGN9, NGN1, NGN8, NGN4, and NGN13), and a third comprised predominantly jugular neuronal populations (JGN1–JGN4). These three neighborhoods were present at similar frequencies across left and right ganglia. A rarer niche present in our spatial data was made up of mainly non-neuronal cells (FB4, NGN3, EC2, MGC2, EC3, and FB2). This non-neuronal niche was present only in one section, and in a region of the spatial data that was marked by expression of Hba-a1, suggesting this region likely includes a blood vessel (Figures 1E and S8).

Classifying neuronal clusters

We also characterized neuronal subtypes through their expression of neurotransmitter and neuropeptide-associated genes. As expected, the vast majority of nodose neurons were glutamatergic, with specific clusters expressing distinct elements of the glutamatergic molecular machinery. Of note, while most nodose neuronal types expressed only the vGLUT2 (*Slc17a6*) vesicular glutamate transporter, two nodose clusters and one jugular neuronal cluster expressed both vGLUT1 (*Slc17a7*) and vGLUT2 (Figures 1J and S7), suggesting specialized differences in the synapses associated with these neurons and differences in the probability of glutamatergic release from these neurons in comparison to other neuronal populations.[50] A number of other glutamatergic clusters were also found to be dopaminergic (Figure 1J), and NGN19, marked by the expression of *Slc6a2*, *Npy*, and *Hand2*, lacked expression of glutamatergic genes but expressed *Dbh*, *Ddc*, and *Th* transcripts. *Dbh* encodes dopamine beta-carboxylase, an enzyme that converts dopamine into noradrenaline, suggesting that this neuronal population is likely noradrenergic (and contains the necessary molecular machinery for dopamine synthesis). This cluster was previously identified as representing "sympathetic neurons" by Kupari et al.[30] and Buchanan et al.[37] (Figures S3 and S4). Nodose neurons also expressed genes encoding different combinations of neuropeptides, including *Tac1*, *Npy*, *Nts*, *Cartpt*, and *Adcyap1* (Figures 1J and S7B). *Npy* expression was enriched in NGN19, and this cluster also expressed *Gal*. *Vip* transcripts were found in NGN20, NGN4, and NGN13 (Figure 1J). Regarding notable membrane sensors and channels, a cluster of neurons expressed the Cck A receptor (*Cckar*) (NGN4), and other discrete clusters expressed *Trpa1* (NGN8), *Slc18a3* (NGN12), and *Olfr78* (NGN21).

In addition to classifying neurons by the expression of neurotransmitters and neuropeptides, we used previously reported information to classify each of our neuronal clusters based on the type of expressed sodium channel, the myelination level, the sensor type, and the organ each cluster innervated[24,30,40] (Figures S9 and S10). Of the 26 neuronal clusters, 10 were identified as expressing predominantly Nav1.1 channels and 14 as expressing predominantly Nav1.8 channels, with 2 clusters expressing similar levels of both channels (Figures S9A and S10A). Myelination affects the speed at which action potentials travel along axons and therefore the speed of signaling to the brain. Only 5 neuronal populations were found to be myelinated (JGN3, NGN14, NGN12, NGN10, and NGN21), as determined by expression of *Nefh*, *Cntn1*, *Cntnap1*, and *Ncam1*. Eleven neuronal clusters were unmyelinated, and 10 were lightly myelinated (Figures S9B and S10B). Neurons in the nodose and jugular ganglia sense mechanical and nociceptive information from the periphery and relay this information to the CNS. Altogether, 10/26 neuron clusters were identified as mechanosensors, 10/26 as nocisensors, and 6 as a mix of the two (Figures S9C and S10C).

Finally, nodose and jugular neurons communicate information from different organs to the brain. Using transcriptomic signatures, we used marker genes established by Zhao et al.[40] and Bai et al.[24] to provisionally predict the organ each neuronal population innervated and calculated the proportion of cells expressing these marker genes in every neuronal cluster. These signatures suggested that of the 5 jugular neuronal clusters, JGN3 is likely to transduce information from the gut, JGN5 to innervate the lungs, and the other 3 jugular clusters to have a broader projection pattern. With regard to nodose neurons, the signatures suggested that it was likely that NGN7 innervates the pancreas, NGN15 the heart, NGN21 the jejunum/ileum, and NGN12 the duodenum (Figures S9D and S10D). Previously reported gene markers for vagal afferents innervating the gut[24] were also expressed in another 8 nodose neuronal clusters, which we also classed as likely projecting to the gut. An additional 9 nodose neuronal clusters expressed gut marker genes and organ projection marker genes to one or more other organs and were thus classified as having broad projections. However, while others have verified these marker genes using tracing studies,[15] we have not done so for the specific clusters identified in our analyses, and they must therefore be taken as provisional until such experimental verification is completed.[18,28,29]

Exploring potential vagal to hindbrain pathways

Afferent vagal neurons synapse with neurons of the dorsal vagal complex (DVC) in the hindbrain, predominantly in the nucleus tractus solitarius (NTS) and the area postrema (AP).[27,51] To identify possible interneuronal communications between the nodose ganglia neurons and neuronal populations within the hindbrain, we employed CellChat[52] to explore possible ligand-receptor interactions between NodoMap and a previously published single-cell transcriptomic dataset of the mouse hindbrain.[53] We identified a total of 54 potentially enriched pathways (32 of which are annotated as non-protein signaling or secretory signaling) signaling in the biologically relevant direction (nodose to hindbrain neurons; Table S14). To categorize signaling pathways based on how many neuron types they are signaling from/to, we classified pathways as having a "many" relationship if the number of source or target clusters was greater than 25% of the total clusters (and if less than 25%, then this was a "few" relationship). In this instance, we identified 28 potential pathways with many-to-many nodose-to-hindbrain neuronal connectivity, 12 pathways with a few-to-many relationship, 5 with a many-to-few relationship, and 9 with a few-to-few relationship.

As expected, a common signaling pathway from all nodose neuronal clusters to all hindbrain neuronal clusters was glutamatergic signaling, with no GABAergic signaling identified between

the two regions.[54–56] We investigated whether the analysis identified signaling pathways that might represent previously identified connections between nodose neurons and DVC neurons. Bai et al.[24] demonstrated signaling between Oxtr+ nodose neurons innervating the gut and hindbrain ppg neurons. In NodoMap, we identify 2 nodose neuronal clusters with >10% Oxtr expression, which were also labeled as mechanosensors and annotated to have broad projections: NGN6 and NGN17. CellChat identified that these clusters might communicate with ppg neurons via several signaling pathways, including glutamatergic signaling (Table S14). To focus on nodose-to-AP/NTS-specific signaling pathways, we identified six neuronal clusters in the hindbrain dataset that were highly likely to originate from the AP or NTS, based on expression of AP/NTS-specific marker genes (see STAR Methods). One pathway of particular interest was tachykinin (TAC) signaling, which highlighted 4 potential nodose neuronal source clusters—NGN10, NGN12, NGN14, and NGN16—and 4 potential target clusters in the hindbrain neuronal dataset (few to few, Figure S11A). All four of these nodose clusters have the potential to signal to a specific neuronal population in the DVC: HB_NE_Gcg/Prlr, likely to be GLP-1-producing neurons known to be involved in modulation of appetite,[57] and all 4 were predicted to innervate either the duodenum specifically or the gut more generally (Figure S9D), highlighting a possible gastrointestinal-nodose-DVC signaling pathway via *Tac1*/*Tac1r*.[58,59] This aligns with previous reports that GLP-1-producing neurons in the nucleus of the solitary tract are directly innervated by vagal afferents.[60] Additionally, energy-homeostasis-associated gene (*Enho*) signaling was identified with a few-to-many signaling relationship (Figure S11B). *Enho* encodes the small peptide Adropin, reported to bind and signal via the G protein-coupled receptor 19 (*Gpr19*).[61,62] All nodose neuronal clusters expressed at least some levels of *Enho*, with potential target clusters in the hindbrain including HB_NE_Tbx20/Prph, thought to be NTS specific due to its expression of transcription factor *Phox2b*. A list of all identified potential signaling pathways, their ligands and receptors, source and target clusters, and their classifications is shown in Table S14. However, anatomical mapping studies are required to determine how many of these putative cellular connections reflect actual physiological signaling pathways.

The effects of fasting on nodose neurons

The vagus nerve plays a crucial role in the gut-brain axis, regulating feeding behavior in response to food ingestion.[20,24,63] Fasting has previously been reported to alter the expression of specific genes associated with energy homeostasis in the nodose ganglia,[31,34,64,65] but the changes occurring in individual neuronal types are largely unknown. We performed snRNA-seq on the nodose ganglia of mice fasted overnight and compared the expression profile to that from *ad libitum*-fed mice, using data from 598 nuclei from overnight fasted mice and 1,126 from *ad libitum*-fed animals. These nuclei were distributed across nearly all of the clusters we identified across the five datasets (50 out of 52 clusters, with no nuclei found from the MGC6 and NGN20 clusters) (Figures 2A and 2B). As expected, fasting did not alter cell cluster identity, but differential gene expression analysis within each nodose neuronal cluster identified a number of genes upregulated or downregulated in specific nodose neuronal populations by fasting (Figure 2C; Table S5). NGN2, NGN5, NGN8, and NGN10, which are all predicted to project to the gut or to have broad projections including the gut, appeared particularly transcriptionally sensitive to fasting. NGN2 and NGN10 had the greatest number of genes downregulated, and NGN8 and NGN5 had the greatest number upregulated in response to fasting. These changes included the upregulation by fasting of the gene encoding amyloid-like protein 1 (*Aplp1*) in the NGN2 cluster, which has been implicated in the modulation of glucose and insulin homeostasis,[66] and the downregulation of the gene encoding the metabotropic glutamate receptor 8 (*Grm8*) in NGN10 (Figures 2D–2G). Examining specifically the expression of gastrointestinal and pancreatic hormone receptors and nutrient-sensing transporters and receptors, cholecystokinin B receptor (*Cckbr*) was downregulated in NGN1 by fasting, while glucagon-like peptide-1 receptor (*Glp1r*) was upregulated by fasting in NGN14 (Figures S12 and S13; Table S5). Ingenuity Pathway Analysis of the altered expression profiles in NGN2, NGN5, NGN8, and NGN10 found diverse signaling and metabolic pathways were altered in specific clusters, including the upregulation of stress-sensitive pathways such as the eukaryotic initiation factor 2 (*Eif2*) signaling and the response of eukaryotic translation initiation factor 2α kinase 4 (EIF2AK4/GCN2) to amino acid deficiency in NGN5 and the upregulated AMPK signaling in NGN10 (Figure 2H; Table S7).

Differences between left and right nodose neuronal gene expression

Current literature suggests functional differences in the left and right nodose ganglia, with, for example, the left nodose ganglion thought to be more involved in distension-induced satiety and the right nodose ganglion having a greater role in food preference.[27] Differential gene expression was analyzed using in-house data and Buchanan et al.,[37] totaling 11,032 cells or nuclei from the left nodose and 8,411 from the right nodose ganglia (Figures 3A and 3B), distributed across all 52 clusters (Figure 3A). Similar numbers of cell clusters and neighborhoods were observed in both left and right ganglia (Figures 3C–3J). However, all clusters showed differential expression of specific genes between left and right ganglia (Figure 3K; Table S6). In particular, clusters NGN3, NGN9, NGN16, and NGN18 had 1,418, 1,929, 1,714, and 1,278 significantly differentially expressed genes (DEGs), respectively (Figures 3K–3O). Examining once again the expression of gastrointestinal and pancreatic hormone receptors and nutrient-sensing transporters and receptors, cannabinoid receptor 1 (Cnr1), insulin receptor (Insr), and the solute carriers Slc5a3, Slc5a5, and Slc5a7 were significantly enriched in left compared to right nodose ganglia in NGN9 (Figures S14 and S15; Table S6). Pathway analysis on the DEGs in NGN3, NGN9, NGN16 and NGN18 identified a number of signaling pathways enriched in left or right nodose neurons. For example, oxidative phosphorylation and respiratory electron transport were significantly upregulated in right nodose neurons in the NGN3 clusters, indicating the right ganglia may be considered more nutrient sensitive. Glutaminergic receptor signaling pathways, the insulin secretion signaling pathway,

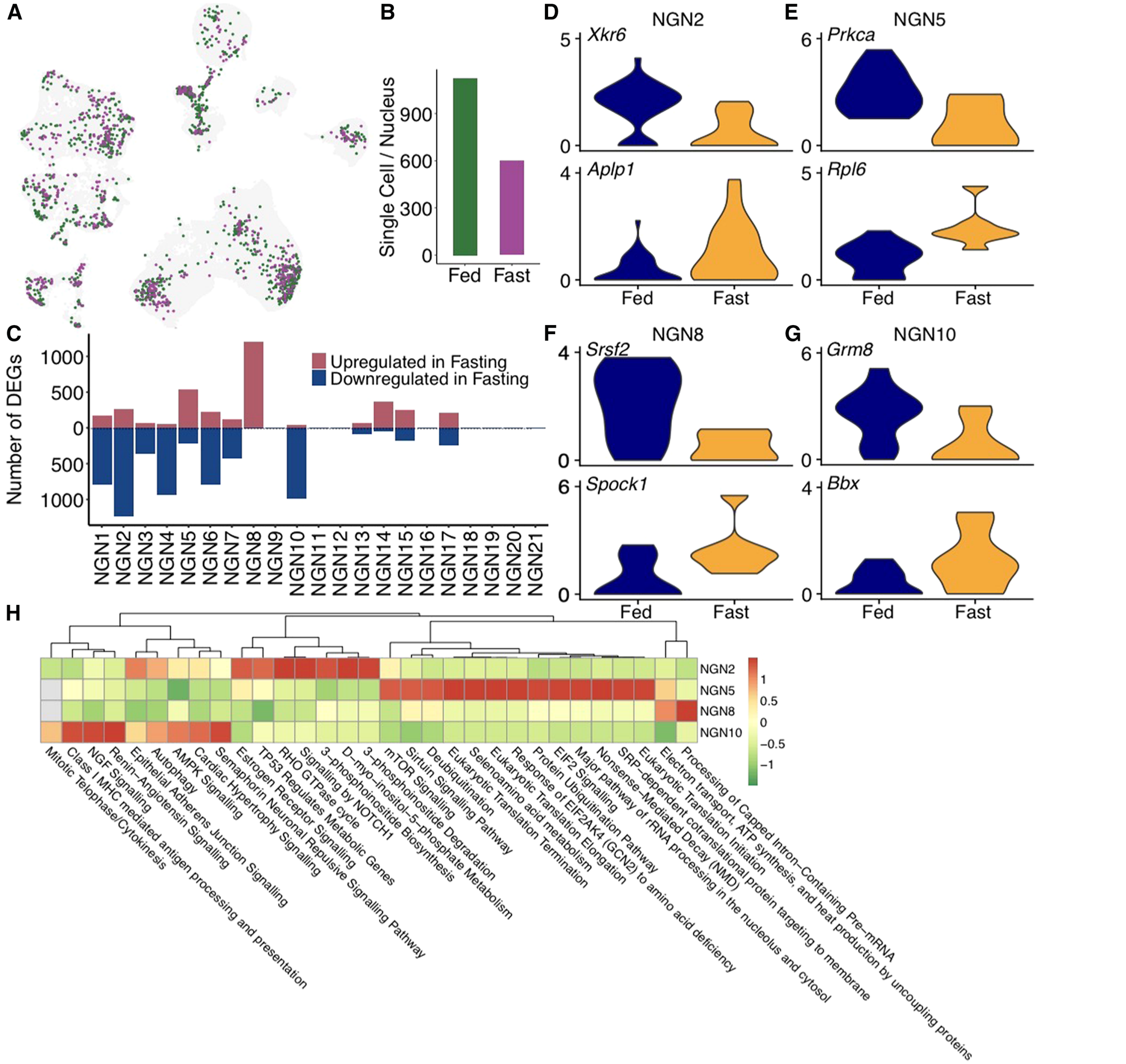

Figure 2. The effects of fasting on nodose ganglion neuronal gene expression

(A) A UMAP plot with cells from *ad libitum*-fed (green) and overnight-fasted (pink) mice highlighted.

(B) The number of nuclei originating from *ad libitum*-fed and overnight-fasted mice.

(C) The number of significantly upregulated (red) and downregulated (blue) genes in each nodose neuronal cluster in response to overnight fasting ($p < 0.05$).

(D–G) The top upregulated and downregulated genes in NGN2, NGN5, NGN8, and NGN10 in fasted animals, selected based on specificity scores and their expression levels (see STAR Methods).

(H) The top 10 pathways that were enriched in the top 4 transcriptionally sensitive nodose neuronal clusters (see STAR Methods).

and serotonin receptor signaling were significantly enriched in right nodose neurons in the NGN9, NGN16, and NGN18 clusters, respectively (Figure 3P; Table S8).

Jugular neurons

In accord with previous analyses, jugular neurons organized into fewer clusters than nodose neurons.[30] Jugular neurons were identified by enriched expression of Prdm12[30] and formed five clusters distinct from the nodose clusters (Figures 4A and 4B) and defined by enrichment of individual genes (Figure 4C). Interestingly, JGN5 expressed the cold and menthol receptor Trpm8, thought to be involved in esophageal vagal sensory signaling.[67] Jugular neuron clusters also showed a number of DEGs in response to fasting and between the left and right ganglia (Figures 4D–4I), though the numbers of DEGs were lower than in nodose neurons. The JGN1 and JGN5 clusters showed the highest number of significantly downregulated (890) and upregulated (612) DEGs in response to fasting, respectively. The

CellPress
OPEN ACCESS

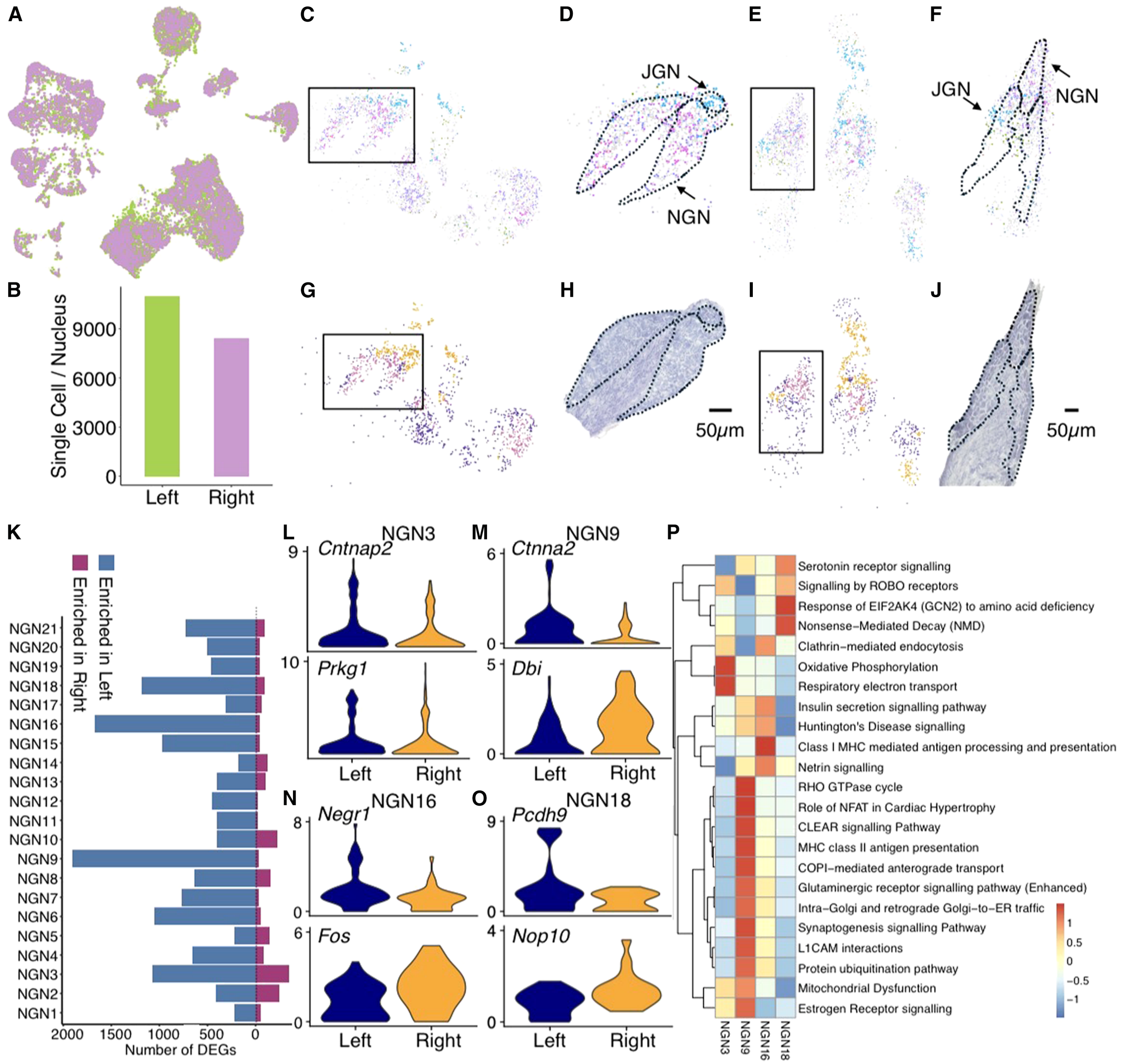

Figure 3. Differences in gene expression between the left and right nodose ganglia

(A) A UMAP plot highlighting cells that originated from left nodose ganglia (green) and right nodose ganglia (lilac).

(B) The number of cells from left or right nodose ganglia within the dataset.

(C and D) RCTD mapping of snRNA-seq clusters on 3 left nodose ganglion sections on 1 tile, with (D) showing a zoomed-in image of the highlighted region in (C). Regions containing nodose or jugular neurons are highlighted.

(E and F) RCTD mapping of snRNA-seq clusters on 3 right nodose ganglion sections on 1 tile, with (F) showing a zoomed-in image of the highlighted region in (E). Regions containing nodose or jugular neurons are highlighted.

(G) Neighborhoods identified in the left nodose ganglia, with NGN neighborhoods labeled in pink, NGN/glia neighborhoods highlighted in purple, and JGN neighborhoods highlighted in orange.

(H) Hematoxylin-stained near-adjacent tissue section of the nodose ganglia highlighted in the box in (C) and (D). NGN and JGN regions are highlighted by dotted lines.

(I) Neighborhoods identified in the right nodose ganglia, with NGN neighborhoods labeled in pink, NGN/glia neighborhoods highlighted in purple, and JGN neighborhoods highlighted in orange.

(J) Hematoxylin-stained near-adjacent tissue section of the nodose ganglia highlighted in the box in (E) and (F). NGN and JGN regions are highlighted by dotted lines.

(legend continued on next page)

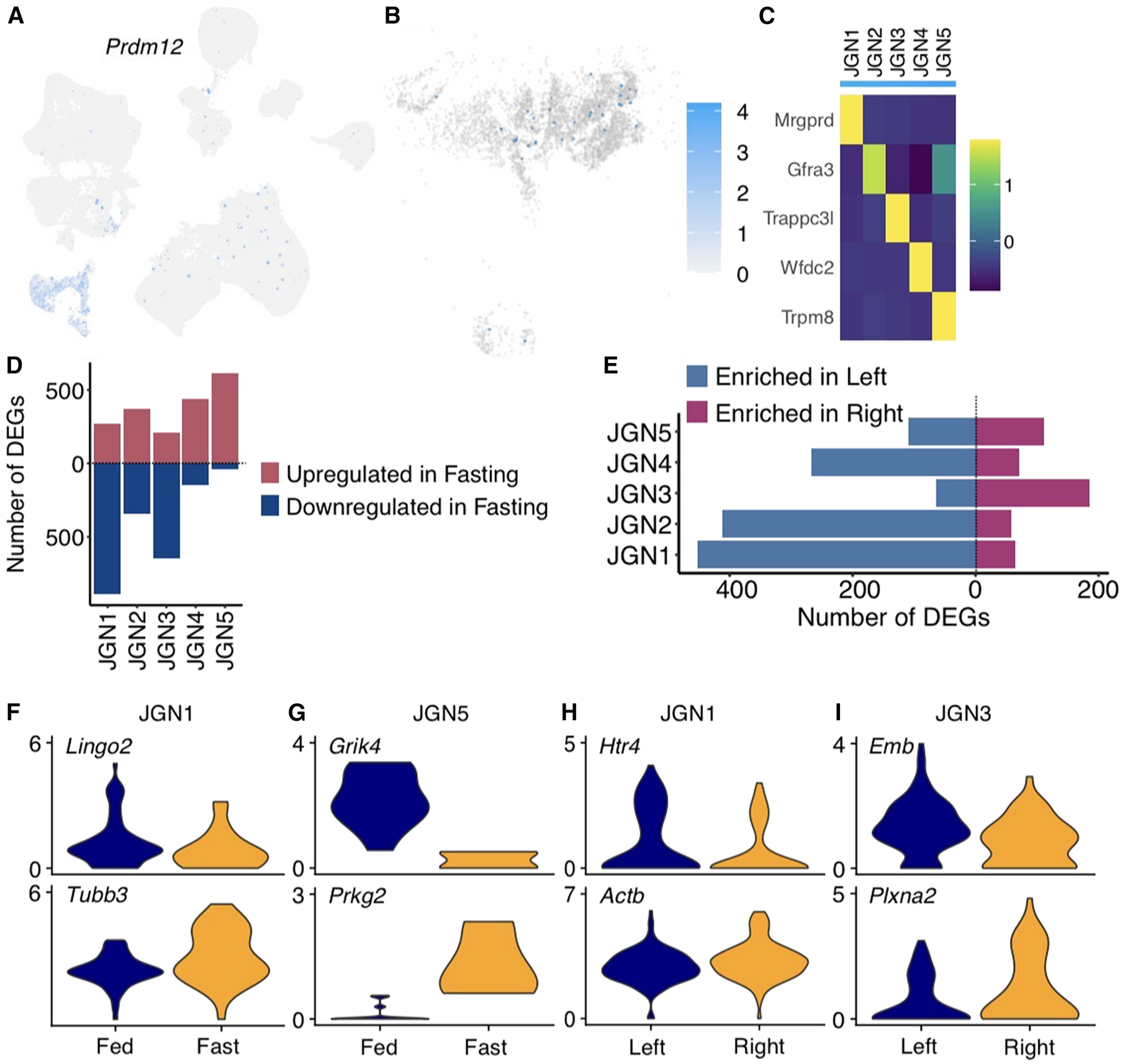

Figure 4. Overview of jugular neurons

(A) UMAP plot highlighting expression of *Prdm12* in NodoMap (log_2 scale).

(B) Log-normalized expression of *Prdm12* in spatial transcriptomics dataset. Cells were colored by the same expression level scale bar as (A).

(C) Heatmap showing scaled expression of five marker genes in jugular ganglion neuronal clusters.

(D and E) Graph showing the number of statistically significantly DEGs ($p < 0.05$) in each jugular neuronal cluster (D) when comparing overnight fasting to *ad libitum*-fed (red: upregulated in fasting, blue: downregulated in fasting) (E) and left vs. right (red: enriched in right nodose ganglia, blue: enriched in left nodose ganglia).

(F–I) Violin plots showing log-normalized expression of top DEGs in (F and G) fed and fasted animals and in (H and I) left vs. right nodose ganglia.

nutrient-sensitive and metabolism pathways altered in response to fasting in JGN clusters were similar to those observed in NGN clusters (Table S16). For example, the response of EIF2AK4 (GCN2) to the amino acid deficiency pathway was upregulated by fasting in JGN1, and the regulation of lipid metabolism by the PPARa pathway was upregulated in JGN5. It is interesting that both jugular neuronal clusters and nodose neuronal clusters, typically not thought to be involved in appetite or metabolic regulation, show extensive changes in gene expression in response to fasting. Such changes may reflect, for example, that fasting can change the circulating levels of neuronal trophic factors[68–71] and cytokines,[72–78] and it can also alter neuronal energy substrate use,[79–82] all of which can alter nodose neuronal activity and gene expression in diverse neuronal populations.[16,19,83–91]

(K–O) DEG analysis comparing left vs. right in nodose neuronal clusters. Some clusters displayed particularly high numbers of DEGs ($p < 0.05$). NGN3, NGN9, NGN16, and NGN18 had 1,418, 1,929, 1,714, and 1,278 significantly DEGs, respectively, with (L–O) showing the top genes enriched in left/right in each of these 4 clusters.

(P) Pathway analysis on the DEGs ($p < 0.05$) in the top 4 transcriptionally sensitive nodose neuronal clusters.

Thus, these effects may represent the broad effects of fasting on, for example, inflammation, growth, and metabolism. JGN1 also had the highest number (451) of DEGs enriched in the left ganglia, and JGN3 had the highest number (185) enriched in the right ganglia. Oxytocin and glutaminergic receptor signaling pathways were downregulated in left jugular neurons in JGN1, and nutrient-sensitive pathways, such as the response of EIF2AK4 (GCN2) to amino acid deficiency, were upregulated in right jugular neurons in JGN3 (Table S17).

Non-neuronal cells

Twenty-six clusters of non-neuronal cell types were identified in both the snRNA-seq and spatial transcriptomic datasets by the presence of the marker genes *Emcn* (endothelial cells), *Ebf2* (fibroblasts), *Ptprc* (hematopoietic cells), *Mpz* (myelinated glial cells), and *Fabp7* (satellite glial cells)[30,92–94] (Figures 5A–5K). Again, specific cell types showed differential gene expression between fed and fasted states and between left and right ganglia (Figures 5L–5Q), though the number of genes with altered expression was typically lower than the number of genes altered in the neuronal clusters. Cluster MGC1 had the highest number of fasting downregulated DEGs (258), and SGC1 had the highest number of fasting upregulated DEGs (357). Intracellular transduction and myelination signaling pathways were either downregulated or upregulated by fasting across different non-neuronal clusters (Table S16). Comparing left and right ganglia, FB3 had the highest number (1,024) of enriched DEGs in the left ganglia, and FB4 the highest number (815) enriched in the right ganglia. Interestingly, estrogen receptor and insulin-like growth factor 1 signaling were both downregulated in the left ganglia of FB3. In contrast, both estrogen receptor- and cholecystokinin/gastrin-mediated signaling were enriched in the right ganglia of FB4 (Table S17).

DISCUSSION

We have harmonized multiple scRNA-seq/snRNA-seq datasets of the mouse nodose ganglia and aligned them with spatial transcriptomics data to create a tool for researchers interested in the physiological and pathophysiological roles of the vagus nerve. Research into vagal signaling has advanced significantly in the last decade,[24,26,55,56,63,95] helped greatly by new molecular approaches, and we hope that NodoMap will facilitate further understanding of this major gut-brain pathway. Harmonizing data from multiple sources is likely to improve the resolution of cell classification, particularly for rarer populations.

NodoMap represents a significant advance over prior individual datasets in several respects. By integrating five independent datasets, we achieve substantially greater cell numbers than any single study, improving statistical power to detect rare cell populations and enabling robust identification of cluster-specific marker genes. The resulting atlas has refined resolution when comparing nodose ganglion neuronal clusters (e.g., NG14 and NG15).[30,37] It includes 52 clusters, of which 26 are neuronal subtypes. Even though we have not had the opportunity to perform similar analyses on human tissue, evidence suggests that human nodose ganglia have characteristics similar to those in the mouse, including expression of key genes and overlapping functional pathways.[96]

The observation that all neuronal clusters exhibit differential gene expression between the left and right nodose ganglia is striking and extends prior reports of functional lateralization in vagal signaling.[26,38,39,97] Left-right differences in vagal circuits have been documented at the level of connectivity, physiology, and behavior. The left vagus has been more strongly implicated in distension-induced satiety signals,[38] while the right has been linked to nutrient preference and reward signals.[26] Our data suggest that these functional differences may have a transcriptional basis, with hundreds of DEGs in some neuronal subtypes. These findings highlight the importance of treating the left and right vagus as distinct entities in experimental design and in interpreting vagal stimulation studies, where the choice of side may have significant consequences for functional outcomes.

CellChat analysis identified a number of potential signaling pathways between the vagus and the hindbrain. Although a number of specific vagal-brainstem pathways have been characterized,[55,98] there are likely to be many more, with the role of, for example, 2-AG and prostaglandin[99] synthesized in nodose neurons having been little examined and the roles of neuropeptide signaling from the vagus to the hindbrain also remaining largely unknown.

Limitations of the study

We have based our classification of neuron innervation targets on the work carried out by Bai et al.[24] and have not anatomically or functionally confirmed these pathways. While these previous data strongly suggest that the markers used are helpful for this classification, it is possible that not all neurons in a cluster target the assigned organs, and it is unclear how broad and heterogeneous the projection patterns of clusters with multiple organ innervation markers actually are. CellChat analysis likely overestimates possible connections, as the hindbrain dataset used will include many cells not innervated by vagal neurons. However, we hope that this analysis can suggest new pathways to investigate using functional studies. Future experiments using spatial transcriptomics analysis of the DVC, linked to tracing of vagal terminals, may help focus future projects on the most promising circuits.

The spatial transcriptomic analyses we have carried out were performed on a limited number of sections and ganglia, and while the major cell-type assignments were reproducible, the coverage of rarer populations in the spatial data was necessarily limited by the section sampling strategy. It is also possible that using snRNA-seq for our own dataset, rather than scRNA-seq, may result in underrepresentation of some transcripts, although both seem to capture the same cell clusters in comparable tissues.[42] Finally, while NodoMap provides a rich transcriptional resource, the links between transcriptional identity and physiological function must ultimately be established through functional studies targeting the specific cell populations identified here.

In summary, NodoMap provides a rich resource for researchers studying vagal signaling. It is intended as a dynamic tool, regularly updated as new data come to light, and to be compared with any future equivalent analysis of the human

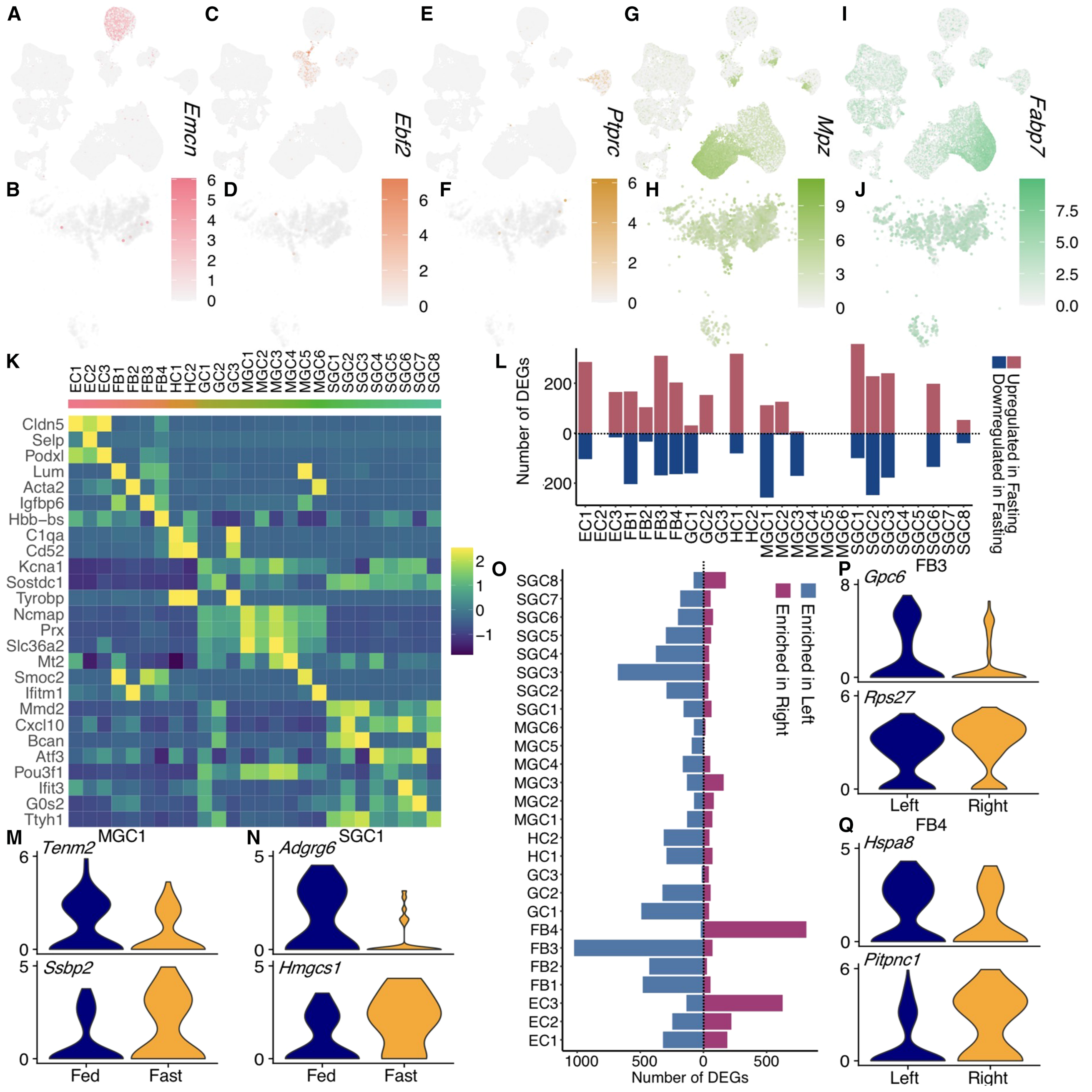

Figure 5. Overview of non-neuronal cells

(A, C, E, G, and I) UMAP plot highlighting expression of non-neuronal cell markers in single-cell atlas (log_2 scale) and (B, D, F, H, and J) a tile of the spatial transcriptomics dataset of 3 left nodose ganglia. For each marker gene, the cells were colored by the same expression scale bar (A and B: *Emcn*, endothelial cells [ECs]; C and D: *Ebf2*, fibroblasts [FBs]; E and F: *Ptprc*, hematopoietic cells [HCs]; G and H: *Mpz*, myelinated glial cells [MGCs]; and I and J: *Fabp7*, satellite glial cells [SGCs]; if both MGC and SGC markers were presented in the clusters, they were labeled as glial cells [GCs]).

(K) Heatmap with scaled expression of marker genes in non-neuronal clusters.

(L) Number of significantly DEGs in fasting across all non-neuronal clusters ($p < 0.05$).

(M and N) Violin plots showing top DEGs in the fasted state.

(O) Number of significantly DEGs comparing left and right nodose ganglia across the non-neuronal clusters ($p < 0.05$).

(P and Q) Violin plots with marker genes enriched in left or right nodose ganglia from the FB3 (P) or FB4 (Q) cluster.

nodose ganglia. We aim for NodoMap to provide a shared reference framework that facilitates the integration of findings across laboratories and experimental approaches and aids researchers seeking to interrogate specific vagal neuronal populations, identify molecular targets, or design genetically targeted experiments. NodoMap will thus hopefully support the discovery of new approaches to exploiting vagal signaling in a clinical context.

RESOURCE AVAILABILITY

Lead contact

Requests for further information and resources should be directed to and will be fulfilled by the lead contact, Kevin G. Murphy (k.g.murphy@imperial.ac.uk).

Materials availability

This study did not generate new unique reagents.

Data and code availability

- Raw sequencing files have been deposited at GEO: GSE296454. The four existing published datasets[24,30,37,40] analyzed are deposited at GEO: GSE124312, GSE138651, GSE185173, and GSE192987. The NodoMap full dataset is available to explore on CellXgene here: https://cellxgene.cziscience.com/collections/982f9f44-031c-4c8c-91ee-dcaa53b10151. The RDS objects are available to download from the University of Cambridge APOLLO repository here: https://doi.org/10.17863/CAM.125125.
- The droplet-based 10× single-cell sequence reads of mouse nodose ganglia were downloaded using FASTQ-dump from the Sequence Read Archive prefetch toolkit (NCBI SRA v.3.0.0).
- Data used to construct figures are provided in Tables S2, S3, S4, S5, S6, S7, S8, S9, S10, S11, S12, S13, S14, S15, S16, S17, S18, and S19.
- All original code for the pre-processing, integration, and plotting of data has been deposited at GitHub and is publicly available at https://github.com/sc2470/NodoMap and at Zenodo at https://doi.org/10.5281/zenodo.20329018.

ACKNOWLEDGMENTS

The Section of Endocrinology and Investigative Medicine is funded by grants from the MRC, BBSRC, and NIHR and is supported by the NIHR Biomedical Research Centre Funding Scheme and the NIHR/Imperial Clinical Research Facility. The views expressed are those of the authors and not necessarily those of the MRC, BBSRC, NHS, NIHR, or Department of Health. K.G.M. is supported by Diabetes UK (18/0005886 and 20/0006295), the BBSRC (BB/W001497/1 and BB/X017273/1), the MRC (MR/Y013980/1), and the Wellcome Trust (310835/Z/24/Z). S.C. was supported by the British Society of Neuroendocrinology. B.Y.H.L. and G.S.H.Y. are supported by BBSRC Project Grant (BB/S017593/1) and the MRC Metabolic Diseases Unit (MC_UU_00014/1). Next-generation sequencing was performed at the IMS Genomics and Bioinformatics Core supported by the MRC (MC_UU_00014/5 and RGAG/542 MC_UU_00039), the Wellcome Trust (208363/Z/17/Z and RGAG/546 226800/Z/22/Z), and the Cancer Research UK Cambridge Institute Genomics Core.

AUTHOR CONTRIBUTIONS

S.C. and G.K.C.D. were responsible for conceptualization, investigation, visualization, formal analysis, project administration, and the writing of the original draft. K.R., M.N., A.G.R., and P.P. were involved in investigation. G.A.B. contributed to visualization and formal analysis. B.Y.H.L. contributed to methodology, visualization, and formal analysis. G.S.H.Y. and K.G.M. were responsible for conceptualization, formal analysis, data curation, manuscript review and editing, supervision, and funding acquisition. All authors were involved in drafting the article or reviewing it critically for important intellectual content and gave final approval of the version to be published.

DECLARATION OF INTERESTS

B.Y.H.L. provides remunerated consultancy for Nuntius Therapeutics. G.S.H.Y. receives grant funding from Novo Nordisk and Amgen, Inc.; he also consults for both Novo Nordisk and Eli Lilly and Company.

STAR★METHODS

Detailed methods are provided in the online version of this paper and include the following:

- KEY RESOURCES TABLE
- EXPERIMENTAL MODEL AND STUDY PARTICIPANT DETAILS
- METHOD DETAILS
  - Nuclei isolation
  - Molecular tagging of nuclei
  - snRNASeq library preparation
  - Tissue preparation for spatial transcriptomics
  - Spatial transcriptomics library preparation
- QUANTIFICATION AND STATISTICAL ANALYSIS
  - Dataset downloads
  - Sequence alignment
  - Dataset quality control
  - Batch correction and clustering
  - Defining cell types
  - Differential gene expression analysis
  - Downstream pathway analysis
  - Neurotransmitter/neuropeptide assignment
  - Assigning neuronal properties
  - Cell-cell communication
  - Spatial transcriptomics pre-processing
  - Integration of the spatial transcriptomics data of mouse left and right nodose ganglia
  - Integration of snRNASeq and spatial transcriptomics data
  - Neighborhood analysis to identify spatial patterns in cell distribution
  - Statistics

SUPPLEMENTAL INFORMATION

Supplemental information can be found online at https://doi.org/10.1016/j.cpblue.2026.100072.

Received: May 11, 2025
Revised: February 3, 2026
Accepted: June 12, 2026
Published: July 20, 2026

REFERENCES

1. Browning, K.N., Babic, T., Toti, L., Holmes, G.M., Coleman, F.H., and Travagli, R.A. (2014). Plasticity in the brainstem vagal circuits controlling gastric motor function triggered by corticotropin releasing factor. J. Physiol. *592*, 4591–4605. https://doi.org/10.1113/jphysiol.2014.278192.
2. Kentish, S.J., and Page, A.J. (2014). Plasticity of gastro-intestinal vagal afferent endings. Physiol. Behav. *136*, 170–178. https://doi.org/10.1016/j.physbeh.2014.03.012.
3. Kollarik, M., Ru, F., and Brozmanova, M. (2010). Vagal afferent nerves with the properties of nociceptors. Auton. Neurosci. *153*, 12–20. https://doi.org/10.1016/j.autneu.2009.08.001.
4. Steinert, R.E., and Beglinger, C. (2011). Nutrient sensing in the gut: Interactions between chemosensory cells, visceral afferents and the

secretion of satiation peptides. Physiol. Behav. *105*, 62–70. https://doi.org/10.1016/j.physbeh.2011.02.039.

5. Shah, A., Carreno, F.R., and Frazer, A. (2014). Therapeutic modalities for treatment resistant depression: Focus on vagal nerve stimulation and ketamine. Clin. Psychopharmacol. Neurosci. *12*, 83–93. https://doi.org/10.9758/cpn.2014.12.2.83.
6. Yuan, H., and Silberstein, S.D. (2016). Vagus Nerve and Vagus Nerve Stimulation, a Comprehensive Review: Part II. Headache *56*, 259–266. https://doi.org/10.1111/head.12650.
7. Láinez, M.J., and Guillamón, E. (2017). Cluster headache and other TACs: Pathophysiology and neurostimulation options. Headache *57*, 327–335. https://doi.org/10.1111/head.12874.
8. Kumar, A., Bunker, M.T., Aaronson, S.T., Conway, C.R., Rothschild, A.J., Mordenti, G., and Rush, A.J. (2019). Durability of symptomatic responses obtained with adjunctive vagus nerve stimulation in treatment-resistant depression. Neuropsychiatr. Dis. Treat. *15*, 457–468. https://doi.org/10.2147/NDT.S196665.
9. Yao, G., Kang, L., Li, J., Long, Y., Wei, H., Ferreira, C.A., Jeffery, J.J., Lin, Y., Cai, W., and Wang, X. (2018). Effective weight control via an implanted self-powered vagus nerve stimulation device. Nat. Commun. *9*, 5349. https://doi.org/10.1038/s41467-018-07764-z.
10. Ogbonnaya, S., and Kaliaperumal, C. (2013). Vagal nerve stimulator: Evolving trends. J. Nat. Sci. Biol. Med. *4*, 8–13. https://doi.org/10.4103/0976-9668.107254.
11. Val-Laillet, D., Biraben, A., Randuineau, G., and Malbert, C.H. (2010). Chronic vagus nerve stimulation decreased weight gain, food consumption and sweet craving in adult obese minipigs. Appetite *55*, 245–252. https://doi.org/10.1016/j.appet.2010.06.008.
12. Burneo, J.G., Faught, E., Knowlton, R., Morawetz, R., and Kuzniecky, R. (2002). Weight loss associated with vagus nerve stimulation. Neurology *59*, 463–464. https://doi.org/10.1212/WNL.59.3.463.
13. Brierley, D.I., and de Lartigue, G. (2022). Reappraising the role of the vagus nerve in GLP-1-mediated regulation of eating. Br. J. Pharmacol. *179*, 584–599. https://doi.org/10.1111/bph.15603.
14. Krieger, J.P., Arnold, M., Pettersen, K.G., Lossel, P., Langhans, W., and Lee, S.J. (2016). Knockdown of GLP-1 receptors in vagal afferents affects normal food intake and glycemia. Diabetes *65*, 34–43. https://doi.org/10.2337/db15-0973.
15. Bonaz, B., Sinniger, V., and Pellissier, S. (2016). Anti-inflammatory properties of the vagus nerve: potential therapeutic implications of vagus nerve stimulation. J. Physiol. *594*, 5781–5790. https://doi.org/10.1113/JP271539.
16. Huerta, T.S., Chen, A.C., Chaudhry, S., Tynan, A., Morgan, T., Park, K., Adamovich-Zeitlin, R., Haider, B., Li, J.H., Nagpal, M., et al. (2025). Neural representation of cytokines by vagal sensory neurons. Nat. Commun. *16*, 3840. https://doi.org/10.1038/s41467-025-59248-6.
17. Goehler, L.E., Gaykema, R.P.A., Opitz, N., Reddaway, R., Badr, N., and Lyte, M. (2005). Activation in vagal afferents and central autonomic pathways: Early responses to intestinal infection with Campylobacter jejuni. Brain Behav. Immun. *19*, 334–344. https://doi.org/10.1016/j.bbi.2004.09.002.
18. Komegae, E.N., Farmer, D.G.S., Brooks, V.L., McKinley, M.J., McAllen, R.M., and Martelli, D. (2018). Vagal afferent activation suppresses systemic inflammation via the splanchnic anti-inflammatory pathway. Brain Behav. Immun. *73*, 441–449. https://doi.org/10.1016/j.bbi.2018.06.005.
19. Jin, H., Li, M., Jeong, E., Castro-Martinez, F., and Zuker, C.S. (2024). A body–brain circuit that regulates body inflammatory responses. Nature *630*, 695–703. https://doi.org/10.1038/s41586-024-07469-y.
20. Waise, T.M.Z., Dranse, H.J., and Lam, T.K.T. (2018). The metabolic role of vagal afferent innervation. Nat. Rev. Gastroenterol. Hepatol. *15*, 625–636. https://doi.org/10.1038/s41575-018-0062-1.
21. Nassenstein, C., Taylor-Clark, T.E., Myers, A.C., Ru, F., Nandigama, R., Bettner, W., and Undem, B.J. (2010). Phenotypic distinctions between neural crest and placodal derived vagal C-fibres in mouse lungs. J. Physiol. *588*, 4769–4783. https://doi.org/10.1113/jphysiol.2010.195339.
22. Zeisel, A., Hochgerner, H., Lönnerberg, P., Johnsson, A., Memic, F., van der Zwan, J., Häring, M., Braun, E., Borm, L.E., La Manno, G., et al. (2018). Molecular Architecture of the Mouse Nervous System. Cell *174*, 999–1014.e22. https://doi.org/10.1016/j.cell.2018.06.021.
23. Bhuiyan, S.A., Xu, M., Yang, L., Semizoglou, E., Bhatia, P., Pantaleo, K.I., Tochitsky, I., Jain, A., Erdogan, B., Blair, S., et al. (2024). Harmonized cross-species cell atlases of trigeminal and dorsal root ganglia. Sci. Adv. *10*, eadj9173. https://doi.org/10.1126/sciadv.adj9173.
24. Bai, L., Mesgarzadeh, S., Ramesh, K.S., Huey, E.L., Liu, Y., Gray, L.A., Aitken, T.J., Chen, Y., Beutler, L.R., Ahn, J.S., et al. (2019). Genetic Identification of Vagal Sensory Neurons That Control Feeding. Cell *179*, 1129–1143.e23. https://doi.org/10.1016/j.cell.2019.10.031.
25. Chang, R.B., Strochlic, D.E., Williams, E.K., Umans, B.D., and Liberles, S.D. (2015). Vagal sensory neuron subtypes that differentially control breathing. Cell *161*, 622–633. https://doi.org/10.1016/j.cell.2015.03.022.
26. Williams, E.K., Chang, R.B., Strochlic, D.E., Umans, B.D., Lowell, B.B., and Liberles, S.D. (2016). Sensory Neurons that Detect Stretch and Nutrients in the Digestive System. Cell *166*, 209–221. https://doi.org/10.1016/j.cell.2016.05.011.
27. Han, W., Tellez, L.A., Perkins, M.H., Perez, I.O., Qu, T., Ferreira, J., Ferreira, T.L., Quinn, D., Liu, Z.W., Gao, X.B., et al. (2018). A Neural Circuit for Gut-Induced Reward. Cell *175*, 665–678.e23. https://doi.org/10.1016/j.cell.2018.08.049.
28. Prescott, S.L., Umans, B.D., Williams, E.K., Brust, R.D., and Liberles, S.D. (2020). An Airway Protection Program Revealed by Sweeping Genetic Control of Vagal Afferents. Cell *181*, 574–589.e14. https://doi.org/10.1016/j.cell.2020.03.004.
29. Tao, J., Campbell, J.N., Tsai, L.T., Wu, C., Liberles, S.D., and Lowell, B.B. (2021). Highly selective brain-to-gut communication via genetically defined vagus neurons. Neuron *109*, 2106–2115.e4. https://doi.org/10.1016/j.neuron.2021.05.004.
30. Kupari, J., Häring, M., Agirre, E., Castelo-Branco, G., and Ernfors, P. (2019). An Atlas of Vagal Sensory Neurons and Their Molecular Specialization. Cell Rep. *27*, 2508–2523.e4. https://doi.org/10.1016/j.celrep.2019.04.096.
31. Burdyga, G., Varro, A., Dimaline, R., Thompson, D.G., and Dockray, G.J. (2006). Feeding-dependent depression of melanin-concentrating hormone and melanin-concentrating hormone receptor-1 expression in vagal afferent neurones. Neuroscience *137*, 1405–1415. https://doi.org/10.1016/j.neuroscience.2005.10.057.
32. Burdyga, G., Varro, A., Dimaline, R., Thompson, D.G., and Dockray, G.J. (2006). Ghrelin receptors in rat and human nodose ganglia: Putative role in regulating CB-1 and MCH receptor abundance. Am. J. Physiol. Gastrointest. Liver Physiol. *290*, G1289–G1297. https://doi.org/10.1152/ajpgi.00543.2005.
33. De Lartigue, G., Dimaline, R., Varro, A., and Dockray, G.J. (2007). Cocaine- and amphetamine-regulated transcript: Stimulation of expression in rat vagal afferent neurons by cholecystokinin and suppression by ghrelin. J. Neurosci. *27*, 2876–2882. https://doi.org/10.1523/JNEUROSCI.5508-06.2007.
34. Burdyga, G., De Lartigue, G., Raybould, H.E., Morris, R., Dimaline, R., Varro, A., Thompson, D.G., and Dockray, G.J. (2008). Cholecystokinin regulates expression of Y2 receptors in vagal afferent neurons serving the stomach. J. Neurosci. *28*, 11583–11592. https://doi.org/10.1523/JNEUROSCI.2493-08.2008.
35. De Lartigue, G., Dimaline, R., Varro, A., Raybould, H., De La Serre, C.B., and Dockray, G.J. (2010). Cocaine- and Amphetamine-Regulated Transcript Mediates the Actions of Cholecystokinin on Rat Vagal Afferent Neurons. Gastroenterology *138*, 1479–1490. https://doi.org/10.1053/j.gastro.2009.10.034.

36. Dockray, G.J., and Burdyga, G. (2011). Plasticity in vagal afferent neurones during feeding and fasting: Mechanisms and significance. Acta Physiol. *201*, 313–321. https://doi.org/10.1111/j.1748-1716.2010.02219.x.

37. Buchanan, K.L., Rupprecht, L.E., Kaelberer, M.M., Sahasrabudhe, A., Klein, M.E., Villalobos, J.A., Liu, W.W., Yang, A., Gelman, J., Park, S., et al. (2022). The preference for sugar over sweetener depends on a gut sensor cell. Nat. Neurosci. *25*, 191–200. https://doi.org/10.1038/s41593-021-00982-7.

38. de Araujo, A., Singh, A., McDougle, M., Braga, I., Leme, G., and de Lartigue, G. (2022). Asymmetric Control of Food Intake by Left and Right Vagal Sensory Neurons. FASEB J. *36*. https://doi.org/10.1096/fasebj.2022.36.s1.00r53.

39. Welch, H.F., Sankaranarayanan, I., Hong, V.M., Qavi, H., Mazhar, K., Kolber, B.J., Price, T.J., and Thorn, C.A. (2025). Molecular and functional asymmetry in Cckar-expressing vagal sensory neurons. Cell Rep. *44*, 116507. https://doi.org/10.1016/j.celrep.2025.116507.

40. Zhao, Q., Yu, C.D., Wang, R., Xu, Q.J., Dai Pra, R., Zhang, L., and Chang, R.B. (2022). A multidimensional coding architecture of the vagal interoceptive system. Nature *603*, 878–884. https://doi.org/10.1038/s41586-022-04515-5.

41. Wu, Y.E., Pan, L., Zuo, Y., Li, X., and Hong, W. (2017). Detecting Activated Cell Populations Using Single-Cell RNA-Seq. Neuron *96*, 313–329.e6. https://doi.org/10.1016/j.neuron.2017.09.026.

42. Bakken, T.E., Hodge, R.D., Miller, J.A., Yao, Z., Nguyen, T.N., Aevermann, B., Barkan, E., Bertagnolli, D., Casper, T., Dee, N., et al. (2018). Single-nucleus and single-cell transcriptomes compared in matched cortical cell types. PLoS One *13*, e0209648. https://doi.org/10.1371/journal.pone.0209648.

43. Yao, Z., Liu, H., Xie, F., Fischer, S., Adkins, R.S., Aldridge, A.I., Ament, S.A., Bartlett, A., Behrens, M.M., Van den Berge, K., et al. (2021). A transcriptomic and epigenomic cell atlas of the mouse primary motor cortex. Nature *598*, 103–110. https://doi.org/10.1038/s41586-021-03500-8.

44. Steuernagel, L., Lam, B.Y.H., Klemm, P., Dowsett, G.K.C., Bauder, C.A., Tadross, J.A., Hitschfeld, T.S., del Rio Martin, A., Chen, W., de Solis, A.J., et al. (2022). HypoMap—a unified single-cell gene expression atlas of the murine hypothalamus. Nat. Metab. *4*, 1402–1419. https://doi.org/10.1038/s42255-022-00657-y.

45. Stuart, T., Butler, A., Hoffman, P., Hafemeister, C., Papalexi, E., Mauck, W.M., Hao, Y., Stoeckius, M., Smibert, P., and Satija, R. (2019). Comprehensive Integration of Single-Cell Data. Cell *177*, 1888–1902.e21. https://doi.org/10.1016/j.cell.2019.05.031.

46. Russell, A.J.C., Weir, J.A., Nadaf, N.M., Shabet, M., Kumar, V., Kambhampati, S., Raichur, R., Marrero, G.J., Liu, S., Balderrama, K.S., et al. (2024). Slide-tags enables single-nucleus barcoding for multimodal spatial genomics. Nature *625*, 101–109. https://doi.org/10.1038/s41586-023-06837-4.

47. Cable, D.M., Murray, E., Zou, L.S., Goeva, A., Macosko, E.Z., Chen, F., and Irizarry, R.A. (2022). Robust decomposition of cell type mixtures in spatial transcriptomics. Nat. Biotechnol. *40*, 517–526. https://doi.org/10.1038/s41587-021-00830-w.

48. Lowenstein, E.D., Misios, A., Buchert, S., and Ruffault, P.-L. (2024). Molecular Characterization of Nodose Ganglia Development Reveals a Novel Population of Phox2b+ Glial Progenitors in Mice. J. Neurosci. *44*, e1441232024. https://doi.org/10.1523/JNEUROSCI.1441-23.2024.

49. Schürch, C.M., Bhate, S.S., Barlow, G.L., Phillips, D.J., Noti, L., Zlobec, I., Chu, P., Black, S., Demeter, J., McIlwain, D.R., et al. (2020). Coordinated Cellular Neighborhoods Orchestrate Antitumoral Immunity at the Colorectal Cancer Invasive Front. Cell *182*, 1341–1359.e19. https://doi.org/10.1016/j.cell.2020.07.005.

50. Johnson, J., Sherry, D.M., Liu, X., Fremeau, R.T., Seal, R.P., Edwards, R.H., and Copenhagen, D.R. (2004). Vesicular glutamate transporter 3 expression identifies glutamatergic amacrine cells in the rodent retina. J. Comp. Neurol. *477*, 386–398. https://doi.org/10.1002/cne.20250.

51. Clyburn, C., and Browning, K.N. (2021). Glutamatergic plasticity within neurocircuits of the dorsal vagal complex and the regulation of gastric functions. Am. J. Physiol. Gastrointest. Liver Physiol. *320*, G880–G887. https://doi.org/10.1152/AJPGI.00014.2021.

52. Jin, S., Guerrero-Juarez, C.F., Zhang, L., Chang, I., Ramos, R., Kuan, C.H., Myung, P., Plikus, M.V., and Nie, Q. (2021). Inference and analysis of cell-cell communication using CellChat. Nat. Commun. *12*, 1088. https://doi.org/10.1038/s41467-021-21246-9.

53. Dowsett, G.K.C., Lam, B.Y.H., Tadross, J.A., Cimino, I., Rimmington, D., Coll, A.P., Polex-Wolf, J., Knudsen, L.B., Pyke, C., and Yeo, G.S.H. (2021). A survey of the mouse hindbrain in the fed and fasted states using single-nucleus RNA sequencing. Mol. Metab. *53*, 101240. https://doi.org/10.1016/j.molmet.2021.101240.

54. Zhuo, H., Ichikawa, H., and Helke, C.J. (1997). Neurochemistry of the nodose ganglion. Prog. Neurobiol. *52*, 79–107. https://doi.org/10.1016/S0301-0082(97)00003-8.

55. Travagli, R.A., and Anselmi, L. (2016). Vagal neurocircuitry and its influence on gastric motility. Nat. Rev. Gastroenterol. Hepatol. *13*, 389–401. https://doi.org/10.1038/nrgastro.2016.76.

56. Young, R.L., Cooper, N.J., and Blackshaw, L.A. (2008). Anatomy and function of group III metabotropic glutamate receptors in gastric vagal pathways. Neuropharmacology *54*, 965–975. https://doi.org/10.1016/j.neuropharm.2008.02.010.

57. Trapp, S., and Brierley, D.I. (2022). Brain GLP-1 and the regulation of food intake: GLP-1 action in the brain and its implications for GLP-1 receptor agonists in obesity treatment. Br. J. Pharmacol. *179*, 557–570. https://doi.org/10.1111/bph.15638.

58. Borgmann, D., Ciglieri, E., Biglari, N., Brandt, C., Cremer, A.L., Backes, H., Tittgemeyer, M., Wunderlich, F.T., Brüning, J.C., and Fenselau, H. (2021). Gut-brain communication by distinct sensory neurons differently controls feeding and glucose metabolism. Cell Metab. *33*, 1466–1482.e7. https://doi.org/10.1016/j.cmet.2021.05.002.

59. Xie, Z., Zhang, X., Zhao, M., Huo, L., Huang, M., Li, D., Zhang, S., Cheng, X., Gu, H., Zhang, C., et al. (2022). The gut-to-brain axis for toxin-induced defensive responses. Cell *185*, 4298–4316.e21. https://doi.org/10.1016/j.cell.2022.10.001.

60. Holt, M.K., Pomeranz, L.E., Beier, K.T., Reimann, F., Gribble, F.M., and Rinaman, L. (2019). Synaptic inputs to the mouse dorsal vagal complex and its resident preproglucagon neurons. J. Neurosci. *39*, 9767–9781. https://doi.org/10.1523/JNEUROSCI.2145-19.2019.

61. Stein, L.M., Yosten, G.L.C., and Samson, W.K. (2016). Adropin acts in brain to inhibit water drinking: Potential interaction with the orphan G protein-coupled receptor. Am. J. Physiol. Regul. Integr. Comp. Physiol. *310*, R476–R480. https://doi.org/10.1152/ajpregu.00511.2015.

62. Rao, A., and Herr, D.R. (2017). G protein-coupled receptor GPR19 regulates E-cadherin expression and invasion of breast cancer cells. Biochim. Biophys. Acta. Mol. Cell Res. *1864*, 1318–1327. https://doi.org/10.1016/j.bbamcr.2017.05.001.

63. Wang, Y.B., de Lartigue, G., and Page, A.J. (2020). Dissecting the Role of Subtypes of Gastrointestinal Vagal Afferents. Front. Physiol. *11*, 643. https://doi.org/10.3389/fphys.2020.00643.

64. Burdyga, G., Lal, S., Varro, A., Dimaline, R., Thompson, D.G., and Dockray, G.J. (2004). Expression of Cannabinoid CB1 Receptors by Vagal Afferent Neurons, Is Inhibited by Cholecystokinin. J. Neurosci. *24*, 2708–2715. https://doi.org/10.1523/JNEUROSCI.5404-03.2004.

65. de Lartigue, G., Barbier de la Serre, C., Espero, E., Lee, J., and Raybould, H.E. (2012). Leptin resistance in vagal afferent neurons inhibits cholecystokinin signaling and satiation in diet induced obese rats. PLoS One *7*, e32967. https://doi.org/10.1371/journal.pone.0032967.

66. Needham, B.E., Wlodek, M.E., Ciccotosto, G.D., Fam, B.C., Masters, C.L., Proietto, J., Andrikopoulos, S., and Cappai, R. (2008). Identification of the Alzheimer's disease amyloid precursor protein (APP) and its

homologue APLP2 as essential modulators of glucose and insulin homeostasis and growth. J. Pathol. *215*, 155–163. https://doi.org/10.1002/path.2343.

67. Yu, X., Hu, Y., Ru, F., Kollarik, M., Undem, B.J., and Yu, S. (2015). TRPM8 function and expression in vagal sensory neurons and afferent nerves innervating guinea pig esophagus. Am. J. Physiol. Gastrointest. Liver Physiol. *308*, G489–G496. https://doi.org/10.1152/ajpgi.00336.2014.

68. Marosi, K., and Mattson, M.P. (2014). BDNF mediates adaptive brain and body responses to energetic challenges. Trends Endocrinol. Metab. *25*, 89–98. https://doi.org/10.1016/j.tem.2013.10.006.

69. Hu, E., Du, H., Zhu, X., Wang, L., Shang, S., Wu, X., Lu, H., and Lu, X. (2018). Beta-hydroxybutyrate Promotes the Expression of BDNF in Hippocampal Neurons under Adequate Glucose Supply. Neuroscience *386*, 315–325. https://doi.org/10.1016/j.neuroscience.2018.06.036.

70. Sleiman, S.F., Henry, J., Al-Haddad, R., El Hayek, L., Abou Haidar, E., Stringer, T., Ulja, D., Karuppagounder, S.S., Holson, E.B., Ratan, R.R., et al. (2016). Exercise promotes the expression of brain derived neurotrophic factor (BDNF) through the action of the ketone body β- hydroxybutyrate. eLife *5*, e15092. https://doi.org/10.7554/eLife.15092.

71. Bastani, A., Rajabi, S., and Kianimarkani, F. (2017). The effects of fasting during ramadan on the concentration of serotonin, dopamine, brainderived neurotrophic factor and nerve growth factor. Neurol. Int. *9*, 7043. https://doi.org/10.4081/ni.2017.7043.

72. Pereira, M., Liang, J., Edwards-Hicks, J., Meadows, A.M., Hinz, C., Liggi, S., Hepprich, M., Mudry, J.M., Han, K., Griffin, J.L., et al. (2024). Arachidonic acid inhibition of the NLRP3 inflammasome is a mechanism to explain the anti-inflammatory effects of fasting. Cell Rep. *43*, 113700. https://doi.org/10.1016/j.celrep.2024.113700.

73. Bouck, T., Monteleone, J., Duffy, J., Ainslie, P.N., Little, J.P., Thomas, K.N., Gibbons, T.D., and Islam, H. (2025). Changes in plasma cytokines following a 60-h fast are not influenced by the addition of exercise despite elevated ketones in healthy young adults. Physiol. Rep. *13*, e70294. https://doi.org/10.14814/phy2.70294.

74. Speaker, K.J., Paton, M.M., Cox, S.S., and Fleshner, M. (2016). A Single Bout of Fasting (24 h) Reduces Basal Cytokine Expression and Minimally Impacts the Sterile Inflammatory Response in the White Adipose Tissue of Normal Weight F344 Rats. Mediators Inflamm. *2016*, 1698071. https://doi.org/10.1155/2016/1698071.

75. Fazeli, P.K., Zhang, Y., O'Keefe, J., Pesaresi, T., Lun, M., Lawney, B., and Steinhauser, M.L. (2020). Prolonged fasting drives a program of metabolic inflammation in human adipose tissue. Mol. Metab. *42*, 101082. https://doi.org/10.1016/j.molmet.2020.101082.

76. Jordan, S., Tung, N., Casanova-Acebes, M., Chang, C., Cantoni, C., Zhang, D., Wirtz, T.H., Naik, S., Rose, S.A., Brocker, C.N., et al. (2019). Dietary Intake Regulates the Circulating Inflammatory Monocyte Pool. Cell *178*, 1102–1114.e17. https://doi.org/10.1016/j.cell.2019.07.050.

77. Janssen, H., Kahles, F., Liu, D., Downey, J., Koekkoek, L.L., Roudko, V., D'Souza, D., McAlpine, C.S., Halle, L., Poller, W.C., et al. (2023). Monocytes re-enter the bone marrow during fasting and alter the host response to infection. Immunity *56*, 783–796.e7. https://doi.org/10.1016/j.immuni.2023.01.024.

78. Almeneessier, A.S., BaHammam, A.A., Alzoghaibi, M., Olaish, A.H., Nashwan, S.Z., and BaHammam, A.S. (2019). The effects of diurnal intermittent fasting on proinflammatory cytokine levels while controlling for sleep/wake pattern, meal composition and energy expenditure. PLoS One *14*, e0226034. https://doi.org/10.1371/journal.pone.0226034.

79. Owen, O.E., Morgan, A.P., Kemp, H.G., Sullivan, J.M., Herrera, M.G., and Cahill, G.F. (1967). Brain metabolism during fasting. J. Clin. Investig. *46*, 1589–1595. https://doi.org/10.1172/JCI105650.

80. Dahlquist, G., and Persson, B. (1976). The rate of cerebral utilization of glucose, ketone bodies, and oxygen: A comparative in vivo study of infant and adult rats. Pediatr. Res. *10*, 910–917. https://doi.org/10.1203/00006450-197611000-00002.

81. Brocchi, A., Rebelos, E., Dardano, A., Mantuano, M., and Daniele, G. (2022). Effects of Intermittent Fasting on Brain Metabolism. Nutrients *14*, 1275. https://doi.org/10.3390/nu14061275.

82. Puchalska, P., and Crawford, P.A. (2017). Multi-dimensional Roles of Ketone Bodies in Fuel Metabolism, Signaling, and Therapeutics. Cell Metab. *25*, 262–284. https://doi.org/10.1016/j.cmet.2016.12.022.

83. Steinberg, B.E., Silverman, H.A., Robbiati, S., Gunasekaran, M.K., Tsaava, T., Battinelli, E., Stiegler, A., Bouton, C.E., Chavan, S.S., Tracey, K.J., and Huerta, P.T. (2016). Cytokine-specific Neurograms in the Sensory Vagus Nerve. Bioelectron. Med. *3*, 7–17. https://doi.org/10.15424/bioelectronmed.2016.00007.

84. Watkins, L.R., Maier, S.F., and Goehler, L.E. (1995). Cytokine-to-brain communication: A review & analysis of alternative mechanisms. Life Sci. *57*, 1011–1026. https://doi.org/10.1016/0024-3205(95)02047-M.

85. Tracey, K.J. (2002). The inflammatory reflex. Nature *420*, 853–859. https://doi.org/10.1038/nature01321.

86. Zhuo, H., and Helke, C.J. (1996). Presence and localization of neurotrophin receptor tyrosine kinase (TrkA, TrkB, TrkC) mRNAs in visceral afferent neurons of the nodose and petrosal ganglia. Brain Res. Mol. Brain Res. *38*, 63–70. https://doi.org/10.1016/0169-328X(95)00313-H.

87. Dinh, Q.T., Groneberg, D.A., Peiser, C., Springer, J., Joachim, R.A., Arck, P.C., Klapp, B.F., and Fischer, A. (2004). Nerve growth factor-induced substance P in capsaicin-insensitive vagal neurons innervating the lower mouse airway. Clin. Exp. Allergy *34*, 1474–1479. https://doi.org/10.1111/j.1365-2222.2004.02066.x.

88. Hunter, D.D., Myers, A.C., and Undem, B.J. (2000). Nerve growth factor-induced phenotypic switch in guinea pig airway sensory neurons. Am. J. Respir. Crit. Care Med. *161*, 1985–1990. https://doi.org/10.1164/ajrccm.161.6.9908051.

89. Weigand, L.A., Kwong, K., and Myers, A.C. (2015). The effects of nerve growth factor on nicotinic synaptic transmission in mouse airway parasympathetic neurons. Am. J. Respir. Cell Mol. Biol. *53*, 443–449. https://doi.org/10.1165/rcmb.2014-0280OC.

90. Pasqualette, L., Fidalgo, T.K.d.S., Freitas-Fernandes, L.B., Souza, G.G.L., Imbiriba, L.A., Lobo, L.A., Volchan, E., Domingues, R.M.C.P., Valente, A.P., and Miranda, K.R. (2024). Alterations in Vagal Tone Are Associated with Changes in the Gut Microbiota of Adults with Anxiety and Depression Symptoms: Analysis of Fecal Metabolite Profiles. Metabolites *14*, 450. https://doi.org/10.3390/metabo14080450.

91. Langhans, W., Egli, G., and Scharrer, E. (1985). Selective hepatic vagotomy eliminates the hypophagic effect of different metabolites. J. Auton. Nerv. Syst. *13*, 255–262. https://doi.org/10.1016/0165-1838(85)90014-1.

92. Mahmoudi, S., Mancini, E., Xu, L., Moore, A., Jahanbani, F., Hebestreit, K., Srinivasan, R., Li, X., Devarajan, K., Prélot, L., et al. (2019). Heterogeneity in old fibroblasts is linked to variability in reprogramming and wound healing. Nature *574*, 553–558. https://doi.org/10.1038/s41586-019-1658-5.

93. Al Barashdi, M.A., Ali, A., McMullin, M.F., and Mills, K. (2021). Protein tyrosine phosphatase receptor type C (PTPRC or CD45). J. Clin. Pathol. *74*, 548–552. https://doi.org/10.1136/jclinpath-2020-206927.

94. Berger, P., Niemann, A., and Suter, U. (2006). Schwann cells and the pathogenesis of inherited motor and sensory neuropathies (Charcot-Marie-Tooth disease). Glia *54*, 243–257. https://doi.org/10.1002/glia.20386.

95. Kaelberer, M.M., Buchanan, K.L., Klein, M.E., Barth, B.B., Montoya, M.M., Shen, X., and Bohórquez, D.V. (2018). A gut-brain neural circuit for nutrient sensory transduction. Science *361*, eaat5236. https://doi.org/10.1126/science.aat5236.

96. Avraham, O., Chamessian, A., Feng, R., Yang, L., Halevi, A.E., Moore, A.M., Gereau, R.W., and Cavalli, V. (2022). Profiling the molecular signature of satellite glial cells at the single cell level reveals high similarities between rodents and humans. Pain *163*, 2348–2364. https://doi.org/10.1097/j.pain.0000000000002628.

97. Berthoud, H.R., Münzberg, H., Morrison, C.D., and Neuhuber, W.L. (2025). Gut-brain communication: Functional anatomy of vagal afferents. Curr. Opin. Neurobiol. *93*, 103058. https://doi.org/10.1016/j.conb.2025.103058.

98. McDougle, M., de Araujo, A., Singh, A., Yang, M., Braga, I., Paille, V., Mendez-Hernandez, R., Vergara, M., Woodie, L.N., Gour, A., et al. (2024). Separate gut-brain circuits for fat and sugar reinforcement combine to promote overeating. Cell Metab. *36*, 393–407.e7. https://doi.org/10.1016/j.cmet.2023.12.014.

99. Bin, N.R., Prescott, S.L., Horio, N., Wang, Y., Chiu, I.M., and Liberles, S.D. (2023). An airway-to-brain sensory pathway mediates influenza-induced sickness. Nature *615*, 660–667. https://doi.org/10.1038/s41586-023-05796-0.

100. McCarthy, D.J., Campbell, K.R., Lun, A.T.L., and Wills, Q.F. (2017). Scater: pre-processing, quality control, normalization and visualization of single-cell RNA-seq data in R. Bioinformatics *33*, 1179–1186. https://doi.org/10.1093/bioinformatics/btw777.

101. Griffiths, J.A., Richard, A.C., Bach, K., Lun, A.T.L., and Marioni, J.C. (2018). Detection and removal of barcode swapping in single-cell RNA-seq data. Nat. Commun. *9*, 2667. https://doi.org/10.1038/s41467-018-05083-x.

102. Lun, A.T.L., Riesenfeld, S., Andrews, T., Dao, T.P., Gomes, T., and participants in the 1st Human Cell Atlas Jamboree; and Marioni, J.C. (2019). EmptyDrops: Distinguishing cells from empty droplets in droplet-based single-cell RNA sequencing data. Genome Biol. *20*, 63. https://doi.org/10.1186/s13059-019-1662-y.

103. Germain, P.L., Lun, A., Garcia Meixide, C., Macnair, W., and Robinson, M.D. (2021). Doublet identification in single-cell sequencing data using scDblFinder. F1000Res. *10*, 979. https://doi.org/10.12688/f1000research.73600.2.

104. Hao, Y., Hao, S., Andersen-Nissen, E., Mauck, W.M., Zheng, S., Butler, A., Lee, M.J., Wilk, A.J., Darby, C., Zager, M., et al. (2021). Integrated analysis of multimodal single-cell data. Cell *184*, 3573–3587.e29. https://doi.org/10.1016/j.cell.2021.04.048.

105. Hafemeister, C., and Satija, R. (2019). Normalization and variance stabilization of single-cell RNA-seq data using regularized negative binomial regression. Genome Biol. *20*, 296. https://doi.org/10.1186/s13059-019-1874-1.

106. Batool, F., and Hennig, C. (2021). Clustering with the Average Silhouette Width. Comput. Stat. Data Anal. *158*, 107190. https://doi.org/10.1016/j.csda.2021.107190.

107. Maechler, M., Rousseeuw, P., Struyf, A., Hubert, M., and Hornik, K. (2023). cluster: Cluster Analysis Basics and Extensions. https://CRAN.R-project.org/package=cluster.

108. Langlieb, J., Sachdev, N.S., Balderrama, K.S., Nadaf, N.M., Raj, M., Murray, E., Webber, J.T., Vanderburg, C., Gazestani, V., Tward, D., et al. (2023). The molecular cytoarchitecture of the adult mouse brain. Nature *624*, 333–342. https://doi.org/10.1038/s41586-023-06818-7.

109. Dobin, A., Davis, C.A., Schlesinger, F., Drenkow, J., Zaleski, C., Jha, S., Batut, P., Chaisson, M., and Gingeras, T.R. (2013). STAR: Ultrafast universal RNA-seq aligner. Bioinformatics *29*, 15–21. https://doi.org/10.1093/bioinformatics/bts635.

110. Curio Bioscience (2023). Curio Seeker pipeline. https://knowledgebase.curiobioscience.com/bioinformatics/pipeline-install/.

STAR★METHODS

KEY RESOURCES TABLE

REAGENT or RESOURCE	SOURCE	IDENTIFIER
Chemicals, peptides, and recombinant proteins		
1M DTT	Sigma	CAT: 646563-10X
100% Triton X-100	Sigma	CAT: T8787-50ML
cOmplete EDTA-free protease inhibitor	Roche	CAT: 11873580001
RnaseIn	Promega	CAT: N2611
Superase.In	ThermoFisher	CAT: AM2696
Draq5	BioStatus	CAT: DR50200
Sucrose	Thermo Scientific	CAT: 419762500
2M KCl	Invitrogen	CAT: AM9640G
1M $MgCl_2$	Invitrogen	CAT: AM9530G
1M Tris Buffer (pH 8.0)	Invitrogen	CAT: AM9855G
Optiprep (60%)	Sigma-Aldrich	CAT: D1556-250ML
Nuclease Free Water	Ambion, Invitrogen	CAT: AM9932
PBS	Invitrogen	CAT: AM9624
BSA	Sigma	CAT: A8412
Tissue-Tek O.C.T. Compound,	Sakura	CAT: 4583
Mayer's Hematoxylin Solution	Sigma-Aldrich	CAT: MHS32
Critical commercial assays		
3′ CellPlex Kit (48 rxns)	10x Genomics	PN-1000261
Chromium Next GEM Single Cell 3′ Kit v3.1	10x Genomics	PN-1000269
Chromium Next GEM Chip G Single Cell Kit	10x Genomics	PN-1000127
Dual Index Kit TT Set A	10x Genomics	PN-1000215
Dual Index Kit NN Set A	10x Genomics	PN-1000243
Curio Seeker 3x3 kit	Curio Bioscience	SQ8002, K001, K002, K003
Nextera XT DNA Library Preparation Kit	Illumina, USA	FC-131-1024
Deposited data		
NodoMap - snRNASeq of mouse nodose ganglion	This paper	Apollo https://doi.org/10.17863/CAM.125125
NodoMap - Curio ST of mouse nodose ganglion	This paper	Apollo https://doi.org/10.17863/CAM.125125
An atlas of vagal sensory neurons and their molecular specialization	NCBI GEO	GSE124312
Genetic identification of vagal sensory neurons that control feeding	NCBI GEO	GSE138651
Single cell sequencing of the nodose ganglia	NCBI GEO	GSE185173
A molecular architecture of the vagal interoceptive system	NCBI GEO	GSE192987
Experimental models: Organisms/strains		
C57BL/6J mice	Charles River, UK	RRID: IMSR_JAX:000664
Software and algorithms		
sra-tools	NCBI SRA	Version 3.0.0
Cellranger	10xGenomics	Version 6.0.1
Scater	bioconductor https://bioconductor.org/packages/release/bioc/html/scater.html	Version 1.32.0

(*Continued on next page*)

Continued

REAGENT or RESOURCE	SOURCE	IDENTIFIER
DropletUtils	bioconductor https://bioconductor.org/packages/release/bioc/html/DropletUtils.html	Version 1.20.0
scDblFinder	bioconductor https://bioconductor.org/packages/release/bioc/html/scDblFinder.html	Version 1.11.4
Seurat	CRAN https://github.com/satijalab/seurat	Version 4.31.1
Sctransform	CRAN	Version 0.3.4
Cluster	CRAN https://cran.r-project.org/web/packages/cluster/index.html	Version 2.1.6
Ingenuity Pathway analysis	QIAGEN	Year 2025
CellChat	https://github.com/jinworks/CellChat	Version 2.1.2
Spacexr	CRAN https://github.com/dmcable/spacexr	Version 2.2.1
ggplot2	CRAN	Version 3.4.2
patchwork	CRAN	Version 1.1.2
dplyr	CRAN	Version 1.1.2
scCustomize	CRAN	Version 1.1.2
scales	CRAN	Version 1.2.1
RColorBrewer	CRAN	Version 1.1–3
hdf5r	CRAN	Version 1.3.8
tidyr	CRAN	Version 1.3.0
cowplot	CRAN	Version 1.1.1
Curio seeker pipeline	Curio Bioscience https://knowledgebase.curiobioscience.com/bioinformatics/pipeline-install/	Version 1.0.4
Other		
2mL Dounce homogenizer	Sigma	D8938-1SET
Superfrost Plus Adhesion Microscope slides	Epredia	N/A

EXPERIMENTAL MODEL AND STUDY PARTICIPANT DETAILS

A total of twenty-six 7-week-old male C57BL/6J mice (Charles River, UK; RRID: IMSR_JAX:000664; twenty for snRNASeq and 6 for ST) were maintained on a 12hr light/dark cycle under a constant temperature (21°C–23°C) with free access to standard chow and water. Mice were group housed in ventilated cages and pathogen free facilities. All animals were acclimatised to the experimental procedures and randomised by body weight. For snRNASeq experiments, ten mice were fasted overnight for 16-h, and another ten mice were *ad libitum* fed prior to nodose ganglia extraction. Mice were randomly assigned to experimental groups by bodyweight.

All animal studies were performed according to the UK Home Office Animals Scientific Procedures Act 1986 (Project License No. PD75F462C) and approved by the Central Biomedical Services unit at the Hammersmith Campus, Imperial College London.

METHOD DETAILS

Nuclei isolation

Mice were euthanized by decapitation at the same time of day and the nodose ganglia were immediately extracted and snap frozen on dry ice pooling overnight-fasted left, overnight-fasted right, *ad libitum* fed left, and *ad libitum* fed right nodose ganglia into four separate 1.5mL Eppendorf tubes. The pooled nodose ganglia samples were stored at −80°C before until nuclei isolation.

Each sample was separately homogenized in 500μL homogenization buffer (100μM DTT, 0.1% Triton X-100, 2x protease inhibitor, 0.4U/μL RNasin, 0.2U/μL Superase.In, 1μL/mL Draq5, and nuclei isolation medium (250mM sucrose, 25mM KCl, 5mM MgCl2, 10mM Tris buffer (pH 8.0) in nuclease-free water) in a 2mL Dounce homogenizer on ice using 10 strokes with pestle A and 10 strokes with

pestle B. Samples were transferred to separate 1.5mL Eppendorf tubes and centrifuged at 900 r.cf. at 4°C for 10 min. The supernatant was carefully removed and pellets were each thoroughly resuspended in a 1:1 mixture of homogenization buffer and 50% Optiprep diluted in iodixanol dilution media (total volume 450μL per sample, final Optiprep concentration was 25%; 250mM sucrose, 150mM KCl, 30mM MgCl2, 60mM Tris buffer (pH 8.0), and nuclease-free water). These suspensions were then each carefully layered on top of separate 450μL 29% Optiprep diluted in iodixanol dilution medium in 2mL Eppendorf tubes and were centrifuged at 13,500 r.cf., at 4°C for 20 min to separate the nuclei and debris. The supernatant was removed, leaving the nuclear pellet. Pellets were resuspended in 1mL PBS containing 0.04% BSA.

Molecular tagging of nuclei

Resuspended samples were centrifuged at 900 r.cf. for 10 min at 4°C and supernatant was removed. Samples were resuspended in 100μL of different cell multiplexing oligo (CMO; from the 10X Genomics 3′ CellPlex kit) and incubated for 5 min at room temperature. The CMO is a feature barcode oligonucleotide connected to a lipid. After incubation, 1.9mL resuspension buffer (PBS with 1% BSA and 0.4U/μL RNasin) was added to each sample, and the mixture centrifuged at 900 r.cf. for 10 min, and then the supernatant was removed. Another 1mL resuspension buffer was added, the mixture centrifuged at 900 r.cf. for 4 min and then the supernatant removed, to remove any unbound CMO. Nuclei pellets were resuspended in 100μL resuspension buffer each and then combined. The pooled sample was centrifuged at 900 r.cf. for 10 min, the top 50μL of supernatant removed, and the pellet resuspended in the rest of the supernatant.

snRNASeq library preparation

The snRNASeq library was prepared according to the Chromium Next GEM Single Cell 3' Reagent Kits v3.1 (Dual Index) with Feature Barcode technology for Cell Multiplexing protocol. Nuclei were loaded onto the Chromium Chip G along with master mix, gel beads and partitioning oil and the chip was placed into the Chromium controller. In the machine, individual nuclei are encapsulated inside partitioning oil along with a single Gel Bead to form GEMs. Each Gel Bead contains oligonucleotides which consist of cell barcodes and unique molecular identifiers (UMI) and a poly(dT) tail which capture mRNA transcripts. A cDNA library is generated through reverse transcription; bead clean up and pre-amplification PCR. The cDNA library is then fragmented enzymatically amplified once more to create the final RNA sequencing library. The snRNASeq library was sequenced on a NovaSeq 6000 (Illumina, USA) at a minimum sequencing depth of 30,000 reads per nucleus.

Tissue preparation for spatial transcriptomics

After cervical dislocation, the left and right nodose ganglia from six mice were dissected and snap frozen on dry ice. Three nodose ganglia in the same orientation were placed on a single disposable base mold (Leica Biosystems, UK) and embedded by adding chilled bubble free optimal cutting temperature compound (Sakura 4583 Tissue-Tek O.C.T. Compound, USA). The samples, comprising 6 left nodose ganglia and 3 right nodose ganglia in total, were stored at −80°C until sectioned on a cryostat at −20°C into 10μm sections (Leica CM1950, Leica Biosystems, UK). The 10μm section was mounted to a pre-chilled 3mm by 3mm Curio Seeker tile slide (Curio Bioscience, USA) for high-resolution spatial transcriptomics.

The 10μm sections sliced before and after the section that was mounted on the Curio Seeker tile were mounted on Superfrost Plus Adhesion Microscope Slides (Epredia, UK) for haematoxylin (Sigma-Aldrich, MHS32) staining to visualise the sample morphology.

Spatial transcriptomics library preparation

The cDNA library was generated according to the Curio Seeker Spatial Mapping Kit (3x3) - User Manual for Fresh Frozen Tissues. Briefly, the tile with tissue mounted on was placed in an Eppendorf containing hybridization mix and incubated to allow for mRNA to hybridize to the barcoded poly (dT) oligos on the lawn of 10μm beads on the tile. Next, reverse transcription occurred to generate double stranded cDNA and the tissues were digested to release the beads into solution. cDNA was amplified, purified and quantified. Next, the Illumina sequencing platform compatible libraries were prepared by using the Nextera XT DNA Library Preparation Kit (Illumina, USA). The library was sequenced on Novaseq 6000 (Illumina, USA, Read1 = 50bp, Read2 = 50bp, Index1 and Index2 = 8bp).

QUANTIFICATION AND STATISTICAL ANALYSIS

Dataset downloads

The droplet-based 10X single cell sequence reads of mouse nodose ganglia from 4 publicly available datasets[24,30,37,40] were downloaded using FASTQ-dump from the sequence read archive prefetch toolkit (NCBI SRA version 3.0.0) (GSE124312, GSE138651, GSE185173, GSE192987).

Sequence alignment

Data was aligned to the mouse transcriptome (GRCm38 mm10, Ensembl 100) using the cellranger count function (version 6.0.1, 10X Genomics, USA). The same reference was used for all datasets (both accessed online, and data created in-house). Sample demultiplexing was performed as follows: Mapping of trimmed 15bp (fastx_trimmer 0.0.13) 10X Nextera CMO reads (R2) to the reference CMO sequences provided by 10X Genomics using bwa's mem command (version 0.7.17). The aligned bam file was then annotated

with cell barcodes (XC) and unique molecular identifiers (XM) extracted from R1 (fastx_trimmer 0.0.13) using fgbio AnnotateBamWithUmis command (version 1.5.1). Reads were then filtered to only those annotated with valid cell barcodes. Only cells with more than 150 valid CMO reads were retained for downstream analyses. The sample assignment and CMO counts are listed in Tables S17 and S18.

Dataset quality control

Scater (Version 1.32.0),[100] DropletUtils (Version 1.20.0),[101,102] scDblFinder (Version 1.11.4),[103] and Seurat (Version 4.3.1)[104] packages were used for data pre-processing: removing low quality cells and identifying and removing doublets, prior to integration. Datasets were filtered on an individual basis to identify and remove low quality cells based on a cut off for the number of genes, UMIs and percentage of mitochondrial genes per dataset. The cut-offs used for each dataset can be found in Table S10. Doublets were identified and removed using scDblFinder. Metadata was added to each dataset, including the source of datasets, the age and the strain of mice, the position of nodose ganglia (left or right), and the nutritional status of the mice.

Batch correction and clustering

Datasets were integrated according to the Seurat integration pipeline for SCTranform normalised data. The pre-processed datasets were imported as a list, normalised using SCTransform (Version 0.3.4).[105] The highly variable gene list was selected by ranking each gene based on the number of datasets it was deemed to be highly variable in. The integration method was iterative pairwise integration. After integrating all nodose ganglia cells/nuclei, batch-corrected datasets were clustered using the Louvain algorithm (FindNeighbors and FindClusters from Seurat Version 4.3.1[104]) and a quality control performed to check the count and feature reads of each cluster. Any clusters with obviously low counts/genes per cell were removed as low-quality clusters. The final integrated nodose single cell/nucleus data had a total of 55,399 genes and 106,436 cells or nuclei.

To optimize the clustering of the dataset, we clustered by computing the k-nearest neighbors based on a set of different number of principal components (PC = 30 to 60, incremental increase by 10), and ran Louvain clustering at differing resolutions (0.6–2, incremental increase by 0.2). The optimal number of clusters was selected based on the average silhouette width[106] (ASW) of overall and *Phox2b* expressed clusters. *Phox2b* is a marker gene of nodose ganglia neurons (NGN).[30] ASW measures the distance between cells in one cluster to the cells in the neighboring clusters with a range of [-1, 1]. The closer of ASW to +1, the more distant the cells are from the neighboring clusters, and the negative ASW suggests the cells are assigned to the wrong clusters. In summary, 1) 25,000 cells were randomly selected from the dataset analyzed for each PC and resolution; 2) their coordinates from 1 to 50 PCs were used to calculate the distances were calculated using the first 50 PCs between cells to generate a dissimilarity matrix. Silhouette widths were calculated based on the dissimilarity of cells using silhouette function from Cluster (Version 2.1.6)[107]; 3) step 1) and 2) were repeated five times and all silhouette widths averaged to get the overall ASW; 4) for cells calculated from the same PC and resolution, their overall ASWs were aggregated and averaged. The averaged overall ASW of each PC and resolution were plotted together into a line graph. Additionally, to identify the best clustering for nodose ganglia neurones, the following steps were performed: 5) the silhouette widths calculated and repeated from step 2) were aggregated by clusters and averaged to get the per-cluster ASW; 6) the per-cluster ASW of clusters with proportion of cells expressing *Phox2b* > 15% were recorded; 7) the *Phox2b* positive cluster ASWs were aggregated and averaged based on corresponding PCs and resolutions, and plotted as a line graph. Based on the overall and *Phox2b* positive clusters' ASWs, the clusters generated using 50 PCs and resolution = 1.4 was regarded as the optimal model with a total of 53 clusters. (Figures S1B and S1C). After downstream quality assessment, one NGN cluster was identified to be a cluster of low-quality cells due to low gene expression and UMI/cell in comparison to other NGN clusters, low-level expression of the nodose neuron marker gene *Phox2b*, and relatively poorly defined marker genes. This cluster was removed from the atlas and downstream analysis was performed on this cleaned dataset, with a total of 52 clusters.

Defining cell types

The gene markers for neuronal cells were *Phox2b* for nodose ganglia neurons (NGN) and *Prdm12* for jugular ganglia neurons (JGN).[30] For non-neuronal cells, *Emcn* was used for endothelial cells (EC),[30] *Acta2* and *Ebf2* for Fibroblasts (FB),[92] *Ptprc* for hematopoietic cells (HC),[93] *Mpz* for myelinated glial cells (MGC),[94] and *Apoe*, *Dbi*, and *Fabp7* for satellite glial cells (SGC).[30] Additionally, a cluster was regarded as glial cells (GC) if the marker genes of MGC and SGC were both highly expressed (Table S11). The expression of each marker gene was visualized, and a histogram of expression levels was used to characterise cell clusters.

Differential gene expression analysis

To identify the marker genes for each cluster, the default setting of FindAllMarkers function from Seurat (Version 4.3.1)[104] was used. The DEGs between two clusters of cells were defined by the Wilcoxon Rank-Sum test. Specificity score was calculated as in Steuernagel et al.[44] to identify relevant markers for cluster annotation: the DEGs with the highest per cluster were used as cluster annotations. Genes starting with *Gm-*, ending with *-Rik*, or genes used by previous clusters were excluded as per.[44]

Differentially expressed genes in cells or nuclei from *ad libitum* fed or overnight fasted, or from left or right nodose ganglia, were identified using the FindMarkers function of Seurat (Version 4.3.1)[104] with the Wilcoxon Rank-Sum test. The number of statistically significantly upregulated ($p < 0.05$; Average $\log_2$Foldchange >0) and downregulated (Average $\log_2$Foldchange <0) DEGs from each cluster were plotted.

Downstream pathway analysis

The ingenuity pathway analysis (IPA, QIAGEN, Germany) was used to identify pathways that were upregulated or downregulated under fed and fasted conditions, or what pathways were differentially regulated in the left or right nodose ganglia. The DEG lists used for pathway analysis were from the two NGN clusters with highest number of statistically significantly DEGs ($p < 0.05$) either upregulated (NGN5, NGN8) or downregulated (NGN2, NGN10) in fasting. Similarly, when comparing left to right nodose ganglia, the DEG lists were from the three NGN clusters with the highest number of statistically significantly DEGs ($p < 0.05$) enriched in left nodose ganglia (NGN9, NGN16, NGN18), and the cluster with the highest number of DEGs ($p < 0.05$) enriched in right nodose ganglia (NGN3). The pathways with top 10 -log(B-H *p* value) from each cluster were grouped and plotted into a heatmap.

Neurotransmitter/neuropeptide assignment

Neuropeptides and neurotransmitters were assigned to individual neuronal clusters based on a set of criteria similar to what was completed in Langlieb et al.[108] For neurotransmitters, neuronal clusters were assigned neurotransmitter identity based on the percentage of cells in a cluster expressing marker genes required for the synthesis/transport of a neurotransmitter. In this instance, a threshold of 30% was used. If a cluster was not assigned any neurotransmitter identity, the expression of neurotransmitter related genes was inspected, and a neurotransmitter was manually assigned. Table S12 indicates the genes used to assign neurotransmitter identity to each cluster. For neuropeptides, expression was defined if over 30% of cells in a cluster had at least 1 transcript of the gene. Some neuropeptides were found to be highly expressed in all clusters, and so higher cut offs were chosen for these genes after manually inspecting a histogram of gene expression across all clusters. The following cut offs were used for high expressing genes: *Cartpt* > 60%; *Adcyap1*, *Calca*, *Calcb*, *Tac1*, *Bdnf* > 50%; *Gal* > 40%.

Assigning neuronal properties

The neuronal clusters were further characterised into different subtypes based on the expression level of sodium channels, mechanosensors and nocisensors, and the marker genes of neuronal fibers and neuronal projection to peripheral organs (Table S14).[24,30,40]

Cell-cell communication

CellChat (2.1.2)[52] was used to investigate the enriched signaling pathways between mouse nodose ganglia and hindbrain neurons. We extracted neuronal clusters from Dowsett et al.[53] and merged these with neuronal clusters from NodoMap to create a CellChat object with nodose and hindbrain neurons. We focused on 'Non-protein Signaling' and 'Secreted Signaling' pathways to look at neurotransmitter and neuropeptide interactions between nodose neurons and hindbrain neurons. Enriched pathways were subsetted to only include pathways that were signaling in the biologically appropriate direction (from nodose to hindbrain), and were split into classifications based the number of clusters each pathway was signaling from/to (many:many, many:few, few:many, few:few). For each enriched pathway, if the number of clusters it was signaling from or to was greater than 25% of the clusters from that region then it was classified as 'many'. To focus on enriched pathways likely to be reaching regions within the dorsal vagal complex (DVC), expression of DVC-specific genes were inspected in clusters from the hindbrain snRNASeq dataset to highlight populations that are most likely from the area postrema, nucleus of the solitary tract, and dorsal motor nucleus of the vagus. We looked at expression of *Phox2b, Glp1r, Gcg, Dbh, Prlh, Gfral* and identified HB_NE_Ddc/Sctr, HB_NE_Tbx20/Prph, HB_NE_Gcg/Prlr, HB_NE_Ddc/Dbh, HB_NE_Gal/Dkk2 and HB_NE_Tfap2b/Olfr78 as DVC specific clusters, and so focused on ligand-receptor pairs that involved these populations.

Spatial transcriptomics pre-processing

After demultiplexing, STAR (version 2.7.5)[109] was used to align the spatial RNA sequencing data to the reference transcriptome GRCm38 (mm10). Following Curio Seeker pipeline (version_2.0.0),[110] the detected cell-associated barcodes, features, and the feature counts of every barcode were generated and saved as rds files for downstream analysis. Each dataset was filtered to keep the spot with more than 100 transcripts and 190 genes.

Integration of the spatial transcriptomics data of mouse left and right nodose ganglia

The integration of spatial transcriptomics datasets followed the same Seurat integration pipeline as the mouse nodose ganglia sc/snRNASeq data using sctransform (Version 0.3.4).[105] The integrated nodose ganglia spatial transcriptomic data had 24,576 features/genes and 9,683 barcodes/spots.

Integration of snRNASeq and spatial transcriptomics data

To visualise the location of different cell types across the nodose ganglia, the Robust Cell Type Decomposition (RCTD)[47] pipeline (spacexr, version 2.2.1) was followed. RCTD is a computational method that uses a sc/snRNASeq data as a reference to characterise the cell types in an ST dataset. From the integrated mouse nodose ganglia sc/snRNASeq dataset, the untransformed count matrix, the cell cluster information (PC = 50, resolution = 1.4), and the total number of counts/transcripts of each cell were extracted as the reference. In the integrated ST data, the spatial coordinates list, the untransformed count matrix, and the total number of counts of

each pixel were used as the query. We ran the RCTD model with a gene cutoff of 0.0001, fc cutoff of 0.25, fc cutoff reg of 0.5, and UMI min sigma of 100 using spacexr (version 2.2.1),[47] the cell cluster information from the reference was used to annotate every spot of the query.

Neighborhood analysis to identify spatial patterns in cell distribution

To identify spatial niches within the ganglia, neighborhood analysis was performed to identify which types of cells commonly appeared together. For each slice, all cells assigned 'reject' by RCTD were first removed, and a k-nearest neighbor (KNN) graph was constructed on the spatial coordinates of each cell (k = 15). For each cell neighborhood, weighted counts (inverse distance) of assigned cell types based on the singlet or doublet assignments were generated to create a celltypeXspatial barcode count matrix. If a cells neighbor was marked as a doublet by RCTD, each assignment was treated as a separate count with equal weighting. The new count matrices for each slice were then concatenated. This matrix was then scaled, regressing out any batch effects across slices, and clustering was run on 10 PCs at a resolution of 0.2 to identify four spatial niches. To identify what cell types were enriched in each niche, Seurat's 'FindMarkers' function was run on the niches.

Statistics

Detailed statistical analyses are described in the sections respectively. For differential gene expression analysis, Seurat (Version 4.3.1)[104] in R (Version 4.4.0) were used to perform the Wilcoxon Rank-Sum test. The specificity score calculation formula was adapted from Steuernagel et al.[44] to define the marker genes of clusters.

CPBLUE, Volume *1*

Supplemental information

NodoMap: A single-cell and spatial transcriptomic atlas of the mouse nodose ganglion

Sijing Cheng, Georgina K.C. Dowsett, Kara Rainbow, Mariana Norton, Anna G. Roberts, Phyllis Phuah, Gavin A. Bewick, Brian Y.H. Lam, Giles S.H. Yeo, and Kevin G. Murphy

Supplementary Figure 1

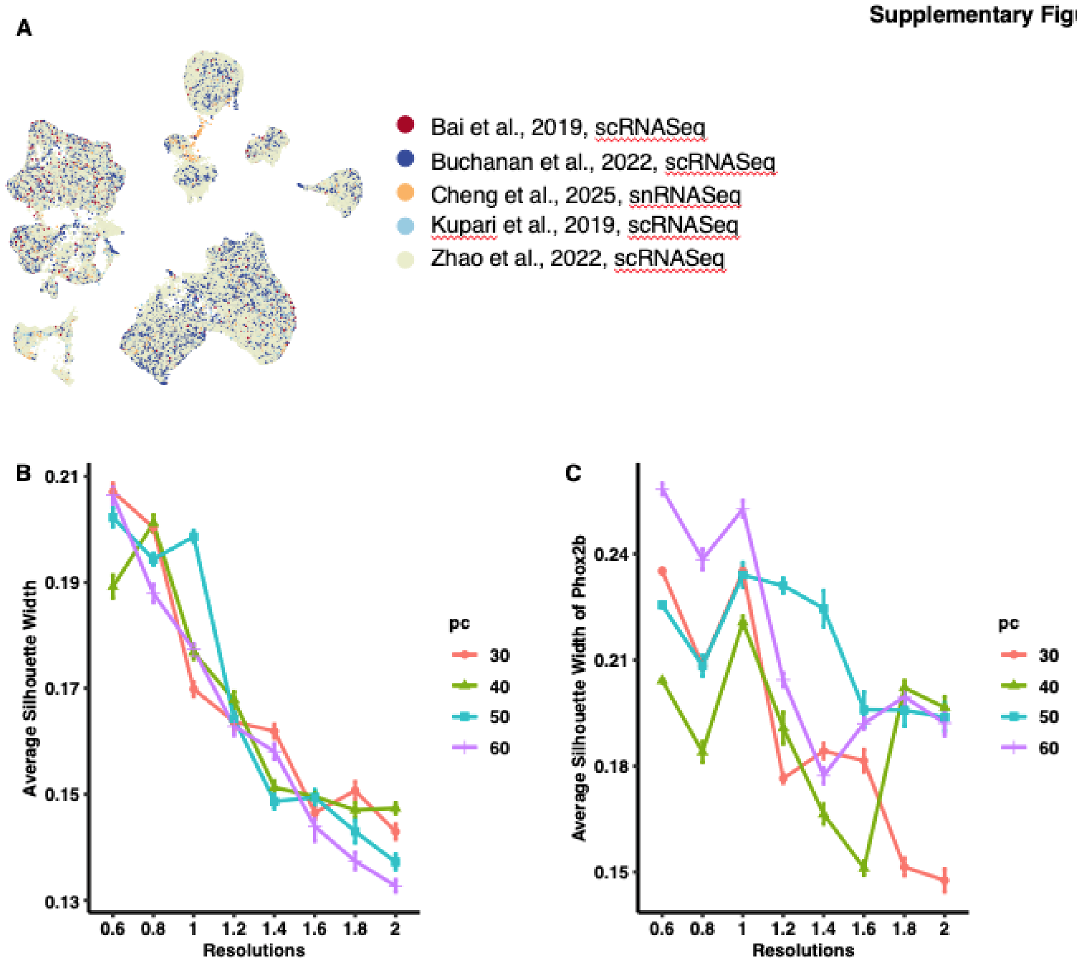

Supplementary Figure 1: Dataset integration quality control. **(A)** UMAP plot coloured by dataset. There were 1979 from Bai et al[15], 17719 from Buchanan et al[28], 1724 from Cheng et al, 4,127 from Kupari et al[14] and 80887 from Zhao et al[31] single cells/nuclei used for downstream analysis. **(B-C)** Line plots of averaged average silhouette width score of all clusters **(B)** and *Phox2b* expressed clusters **(C)** from principal components (PCs) 30 to 60 (increment = 10) and resolutions 0.6 to 2 (increment = 0.2). Together with log-normalised expression of *Phox2b* (NGN marker) and *Prdm12* (JGN marker), single cell atlas defined under PC = 50 and resolution = 1.4 was regarded as the best clustering model.

Supplementary Figure 2

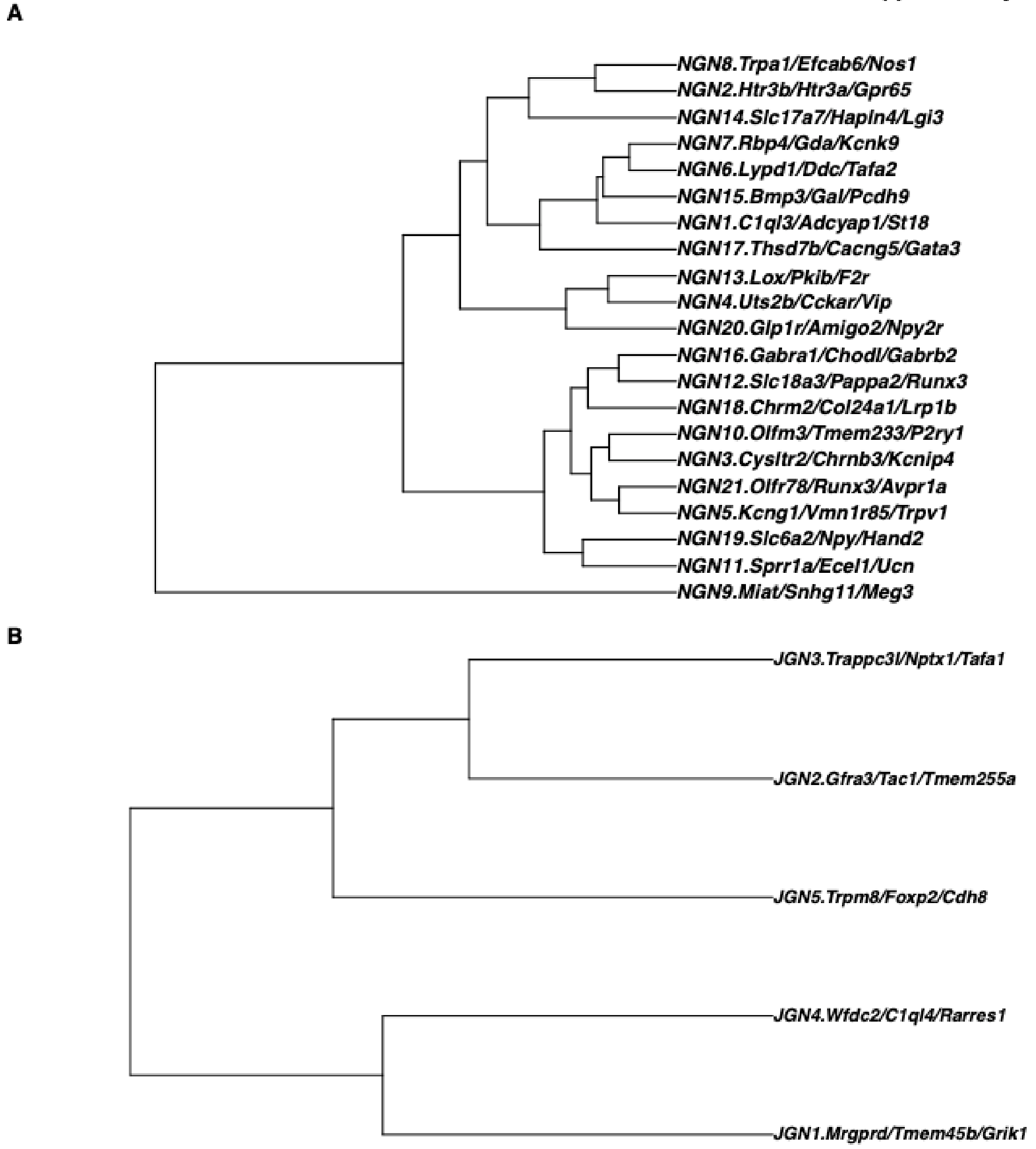

Supplementary Figure 2: Dendrograms of the neuronal clusters. **(A)** The phylogenetic tree plot of the 21 NGN clusters. **(B)** The phylogenetic tree plot of the 5 JGN clusters.

Supplementary Figure 3

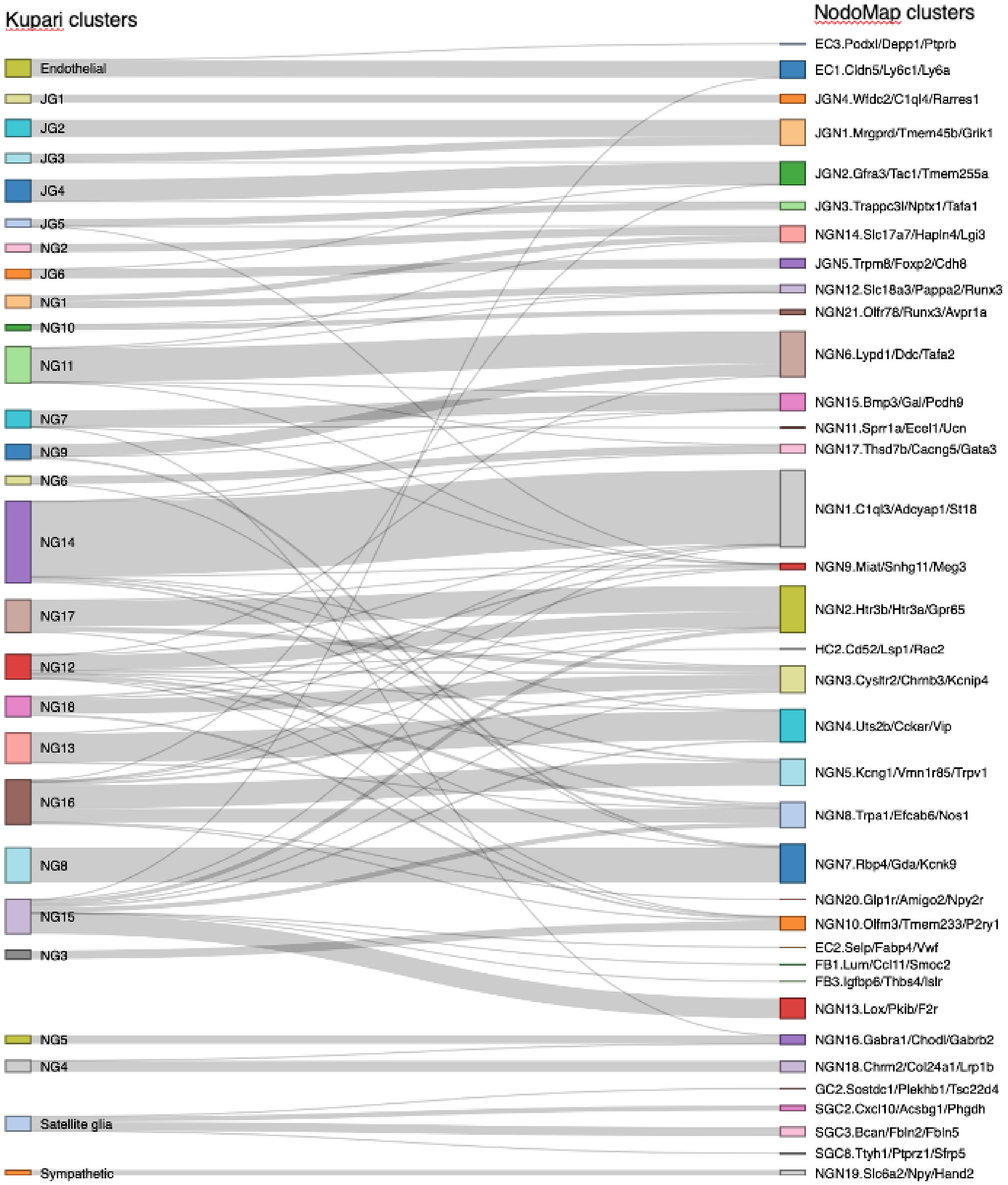

Supplementary Figure 3: Sankey plot highlighting the alignment of the cluster annotation from Kupari et al[14] compared with the annotation based on all five datasets (NodoMap clusters).

Supplementary Figure 4

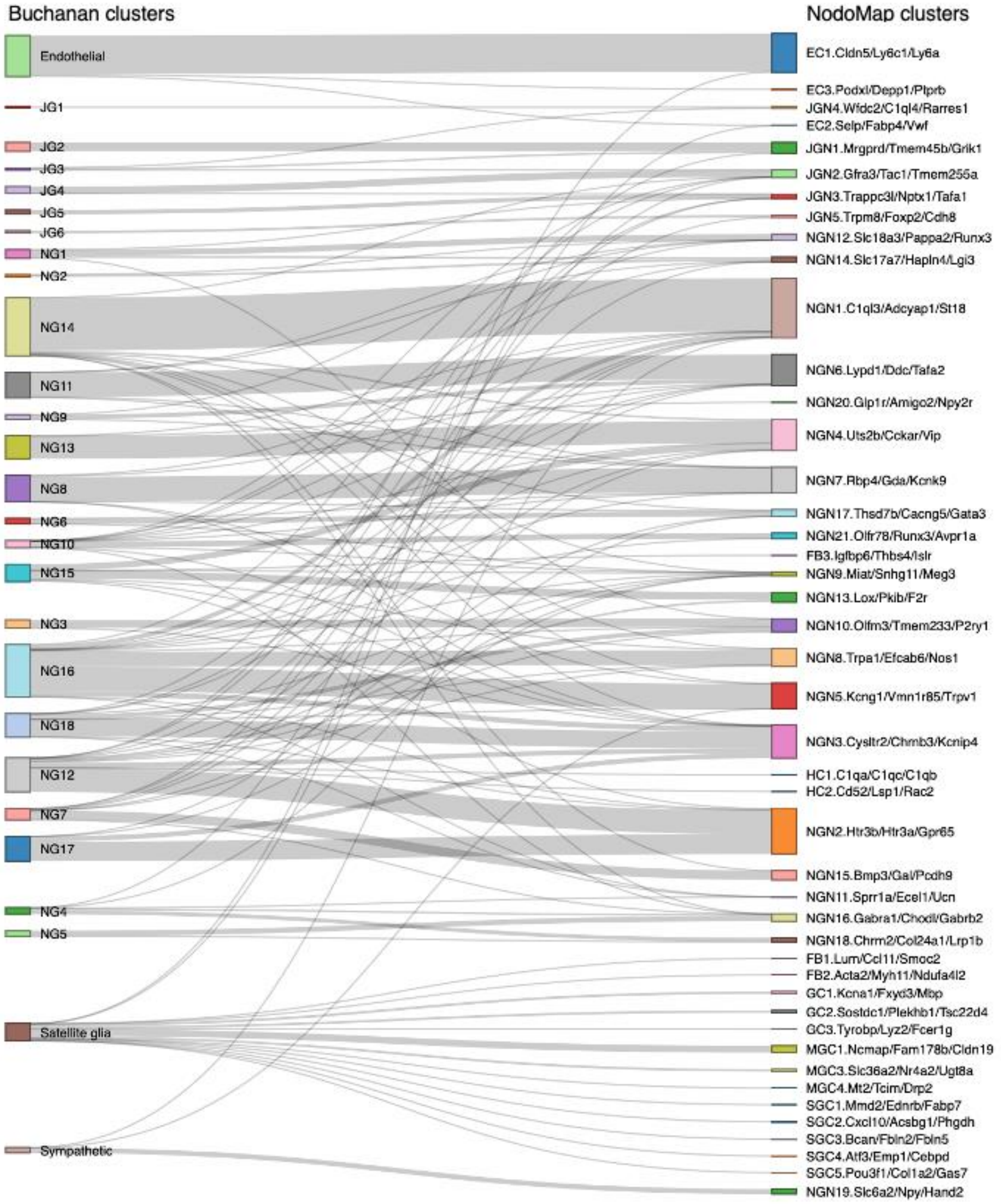

Supplementary Figure 4: Sankey plot highlighting the alignment of the cluster annotation from Buchanan et al[28] compared with the annotation based on all five datasets (NodoMap clusters).

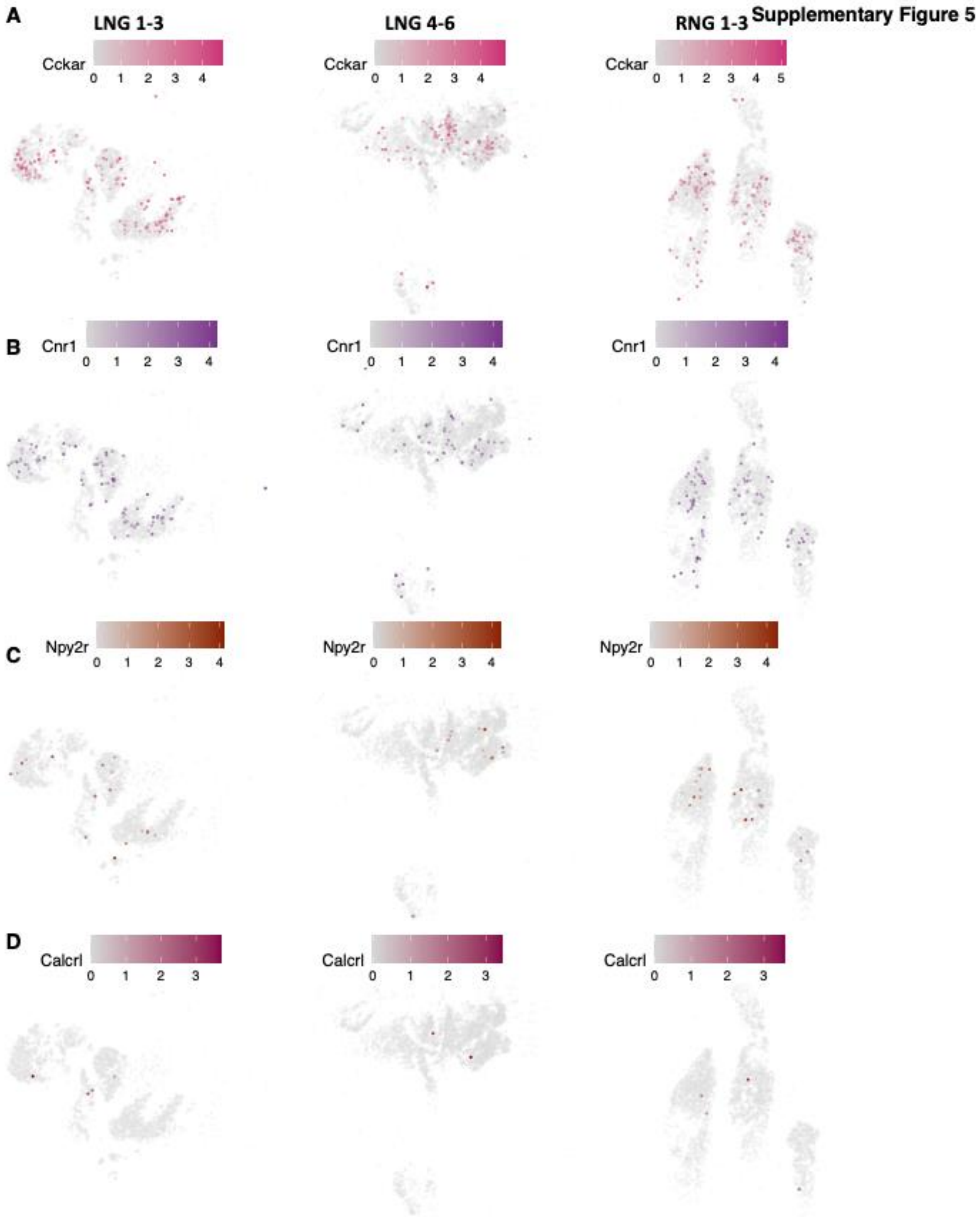

Supplementary Figure 5: Spatial feature plot showing the expression of highly expressed receptors for metabolic signals **(A-D)**. The left and central tiles each were mounted with sections from three left nodose ganglia (six individual left ganglia represented in total). The right tile was a section from three right nodose ganglia (three individual right ganglia represented).

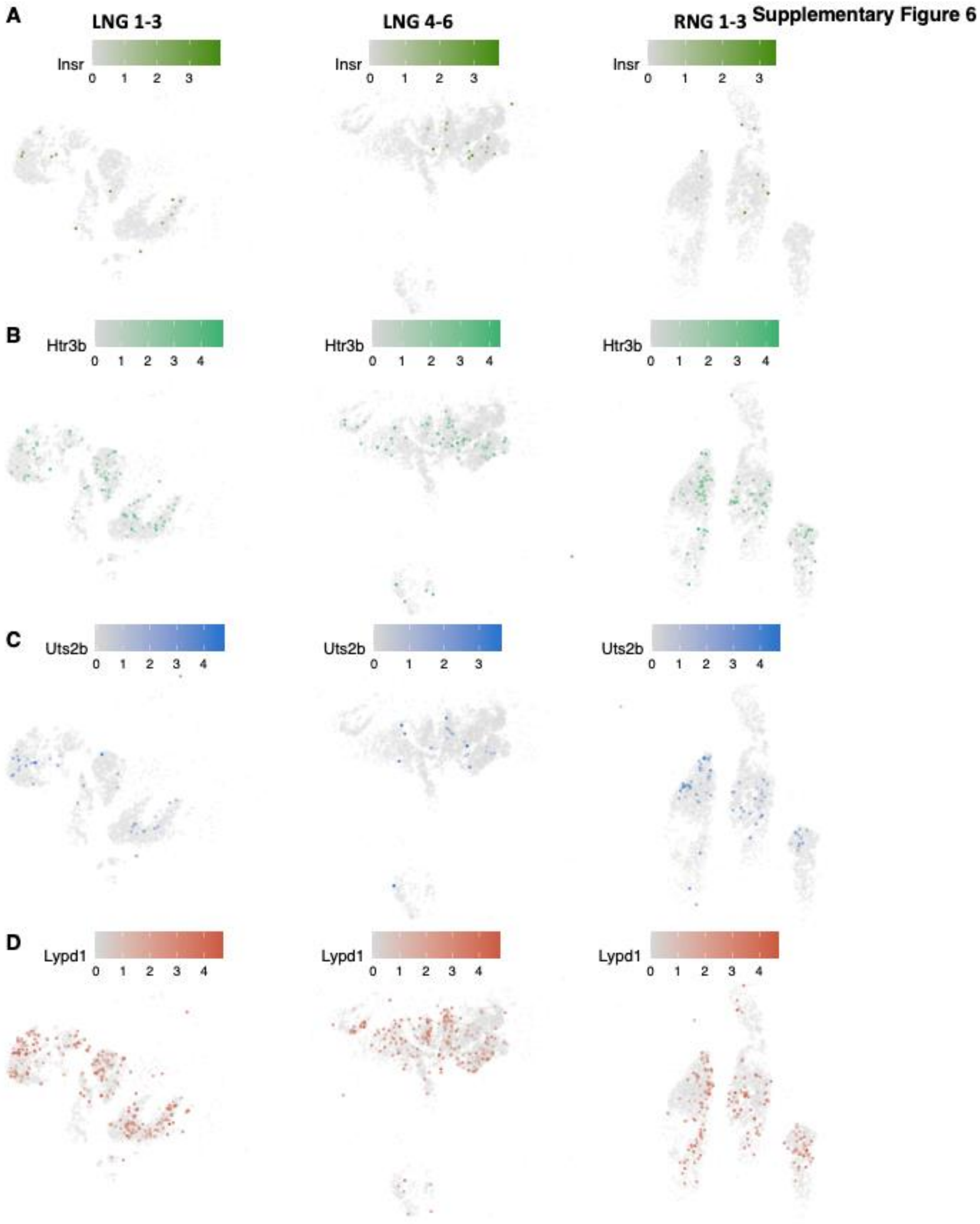

Supplementary Figure 6: Spatial feature plot showing the expression of highly expressed receptors for common NGN cluster markers **(A-D)**. The left and central tiles each were mounted with sections from three left nodose ganglia (six individual left ganglia represented in total). The right tile was a section from three right nodose ganglia (three individual right ganglia represented).

Supplementary Figure 7

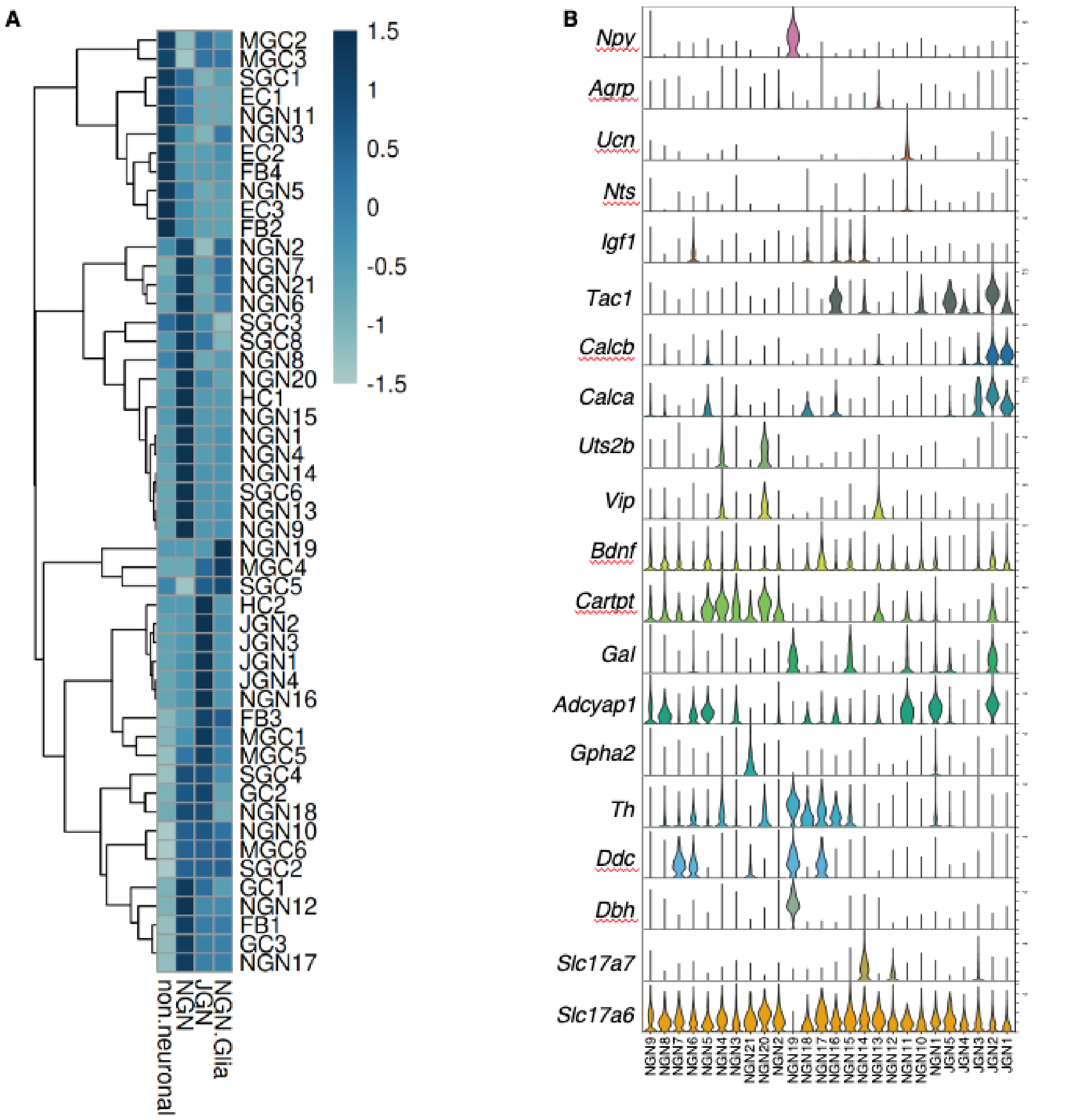

Supplementary Figure 7: Neighbourhood analysis and neuropeptide and neurotransmitter expression across neuronal clusters **(A)** Neighbourhood analysis was performed on the RCTD cell assignments in the spatial transcriptomics data. This formed a total of 4 neighbourhoods which were then labelled based on the co-occurrence of different cell types. The heatmap displays the scaled appearance of assigned cell types within each bin in the spatial transcriptomics dataset. Rows are clustered. **(B)** Violin plot showing expression of neuropeptide- and neurotransmitter-associated genes in nodose and jugular neuronal clusters.

Supplementary Figure 8

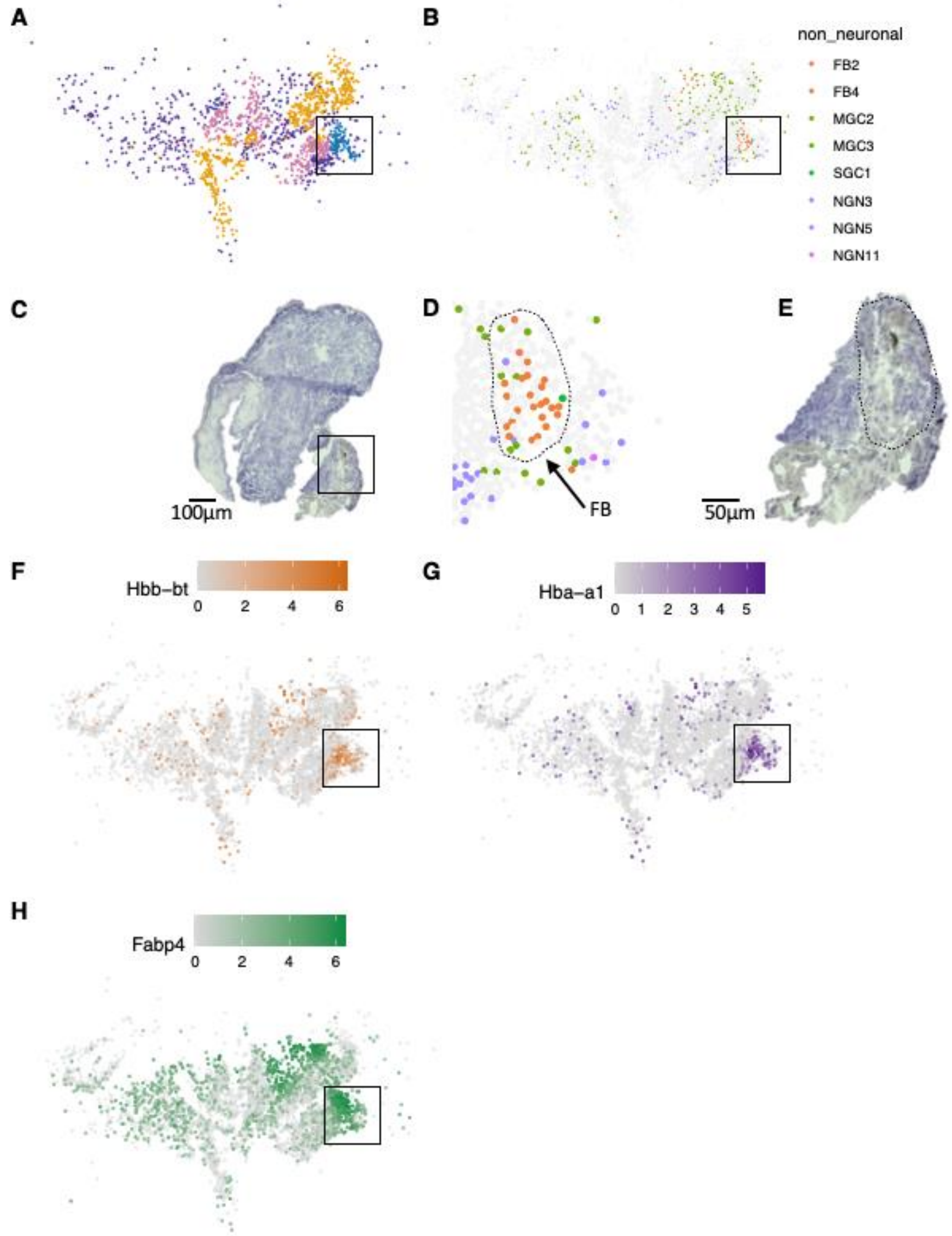

Supplementary Figure 8: Neighbourhood analyses including the non-neuronal niche which likely includes a blood vessel **(A)** Neighbourhood analysis on the RCTD output, with blue to highlight non-neuronal cell types (same as Figure 1E). **(B)** Spatial transcriptomics with RCTD and neighbourhood predicted non-neuronal clusters. **(C)** Haematoxylin staining of an adjacent section of the nodose ganglion. The zoomed-in image of the square section from panel B **(D)**, and panel C **(E)** with the fibroblasts (FB, orange) circled. **(F-H)** Spatial feature plot showing the blood cell marker genes Hbb-bt **(F)** and Hba-a1 **(G)** and the fibroblast marker gene Fabp4 **(H)**.

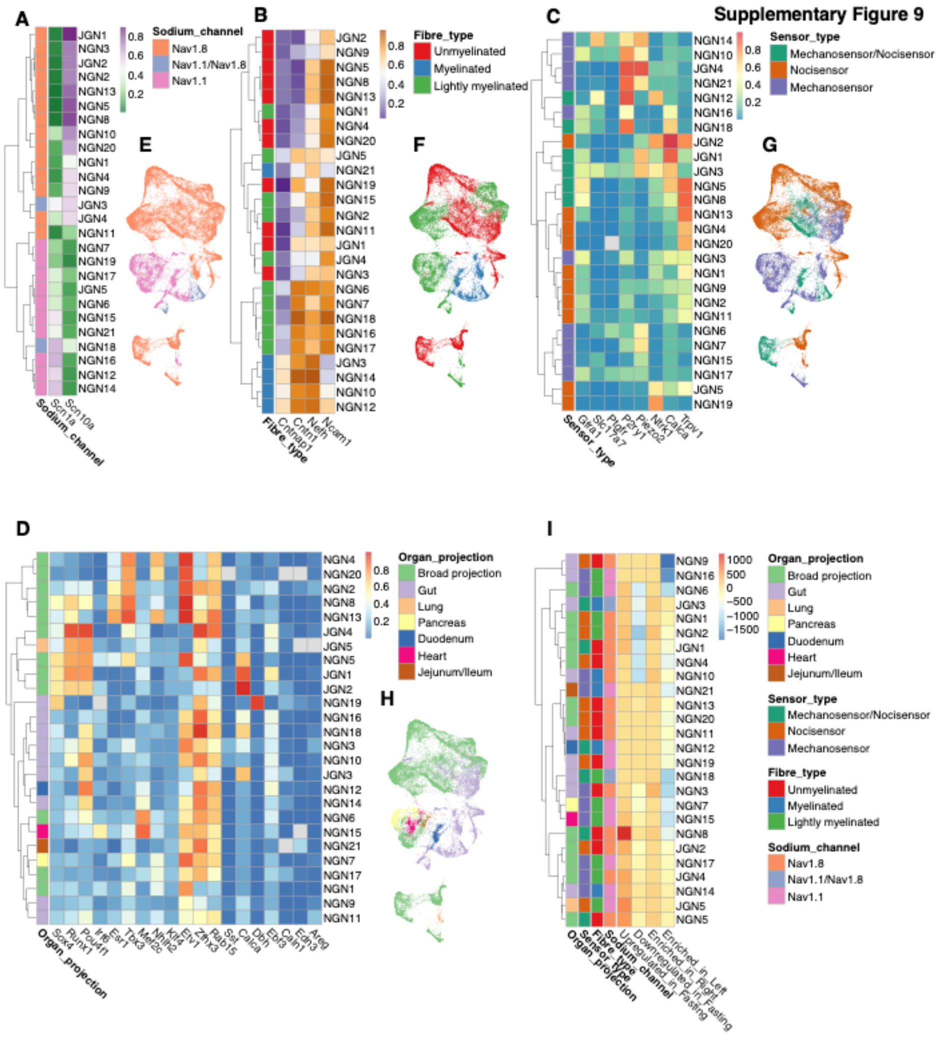

Supplementary Figure 9: Neuronal cluster classifications. **(A-D)** The clustered heatmaps showing the proportion of cells expressing the marker genes within the corresponding neuronal clusters. The neuronal clusters were clustered by the Boolean values. By comparing the expression level of marker genes, the neuronal clusters were further characterised based on their **(A)** sodium channels (Nav1.1 (*Scn1a*) or Nav1.8 (*Scn10a*)); **(B)** fibre types (myelinated or unmyelinated (*Cntnap1*, *Cntn1, Nefh, Ncam1*)); **(C)** sensor types (mechanosensor (*Gfra1, Slc17a7, Ptgfr, P2ry1, Piezo2*) or nocisensor (*Ntrk1, Calca, Trpv1*)); **(D)** organ projections (lung (*Sox4, Runx1, Pou4f1*), heart (*Irf6, Esr1, Tbx3, Mef2c*), pancreas (*Nhlh2, Klf4*), gut (*Etv1, Zfhx3, Rab15*), stomach (*Sst, Calca*), duodenum (*Dbh, Ebf3*), jejunum/ileum (*Caln1, Edn3*), broad projection (marker genes from two or more organs). **(E-H)** UMAP plots of NodoMap neuronal clusters, coloured based on the classification of sodium channels **(E)**, fibre types **(F)**, sensor types **(G)**, and the peripheral organ projections **(H)**. **(I)** The heatmap of 4 neuronal annotations and the number of upregulated/downregulated DEGs in fasting or enriched DEGs in left or right nodose ganglia.

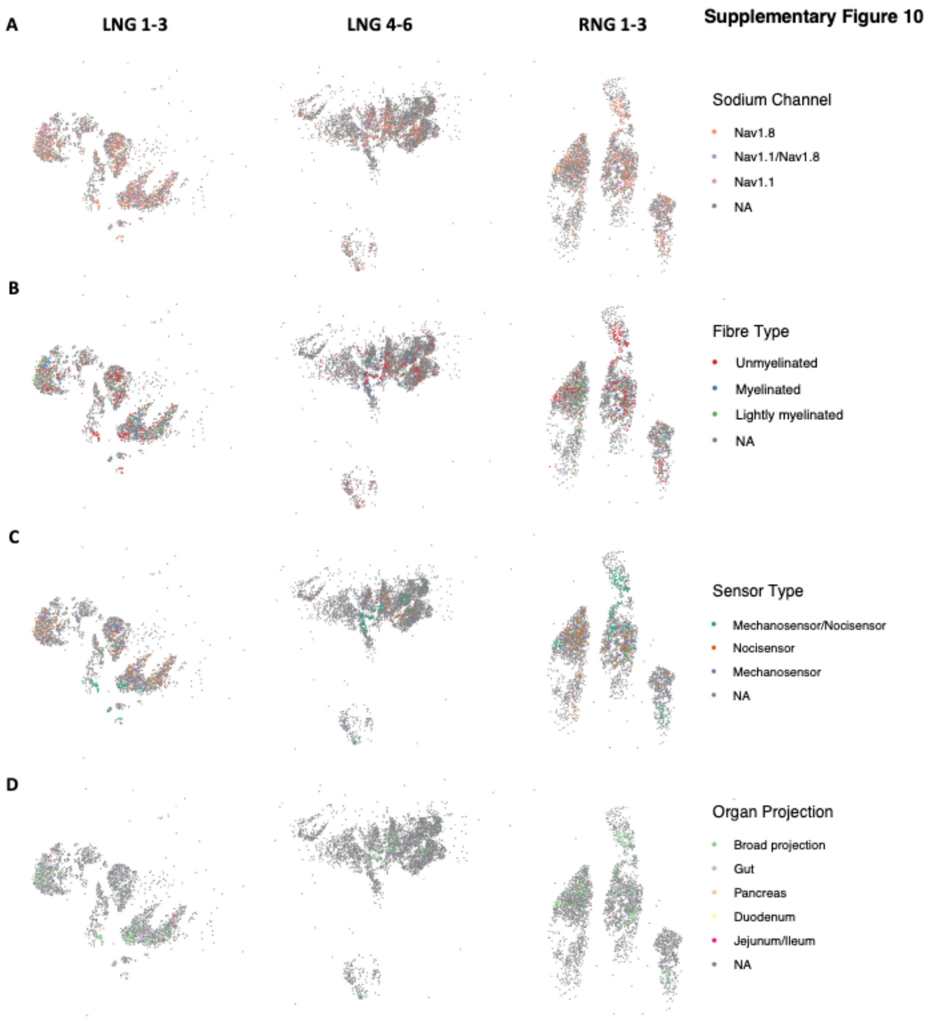

Supplementary Figure 10: Spatial analysis of neuronal cluster classifications **(A)** RCTD annotation of spatial transcriptomics spots, coloured by sodium channel type. **(B)** RCTD annotation of spatial transcriptomics spots, coloured by fibre type. **(C)** RCTD annotation of spatial transcriptomics spots, coloured by sensor type. **(D)** RCTD annotation of spatial transcriptomics spots, coloured by organ projection annotation. The left and central tiles each were mounted with sections from three left nodose ganglia (six individual left ganglia represented in total). The right tile was a section from three right nodose ganglia (three individual right ganglia represented).

Supplementary Figure 11

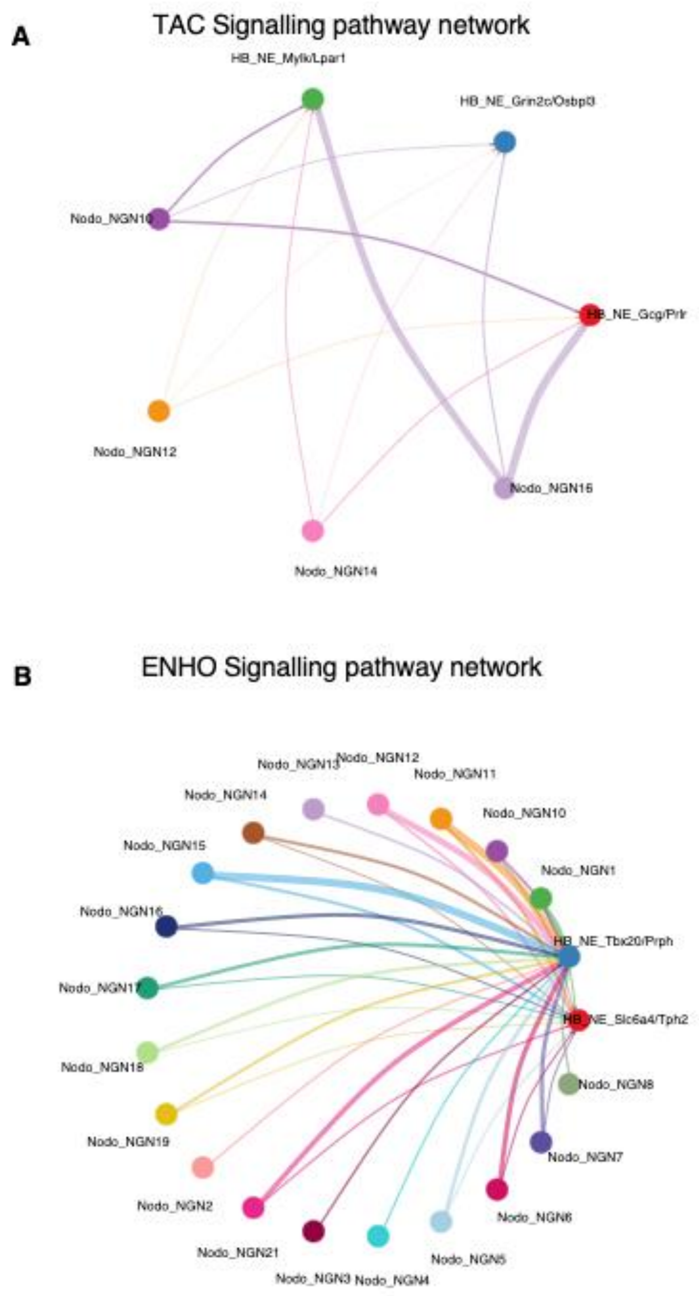

Supplementary Figure 11: CellChat mapping showing potential ligand-receptor interactions between nodose neuronal clusters and hindbrain neuronal clusters for **(A)** TAC signalling and **(B)** ENHO signalling. The thickness of the line represents the relative estimated strength of the signalling.

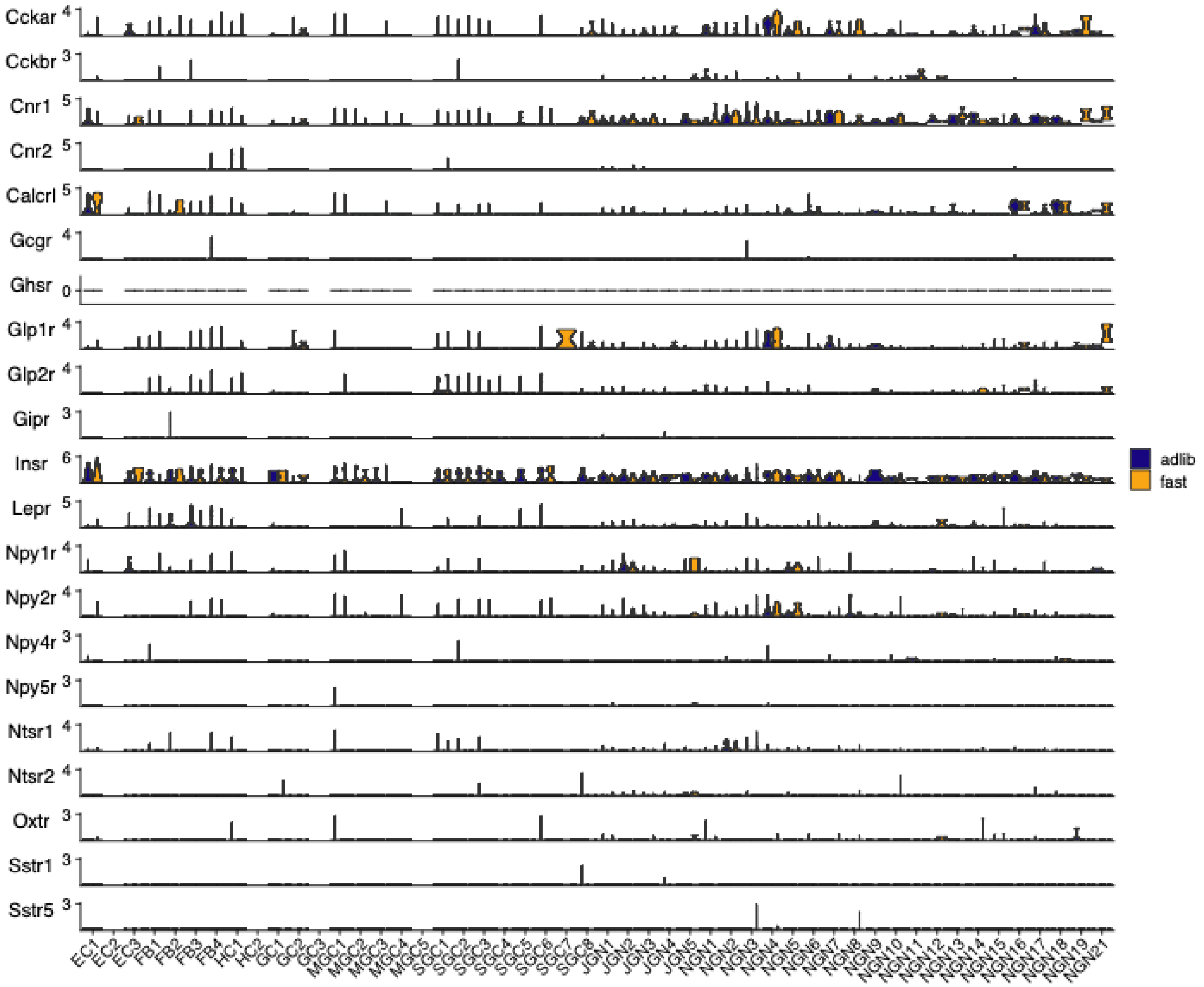

Supplementary Figure 12: Violin plot showing the expression of gastrointestinal and pancreatic hormone receptors in snRNASeq clusters compared between overnight fasting and *ad libitum* feeding.

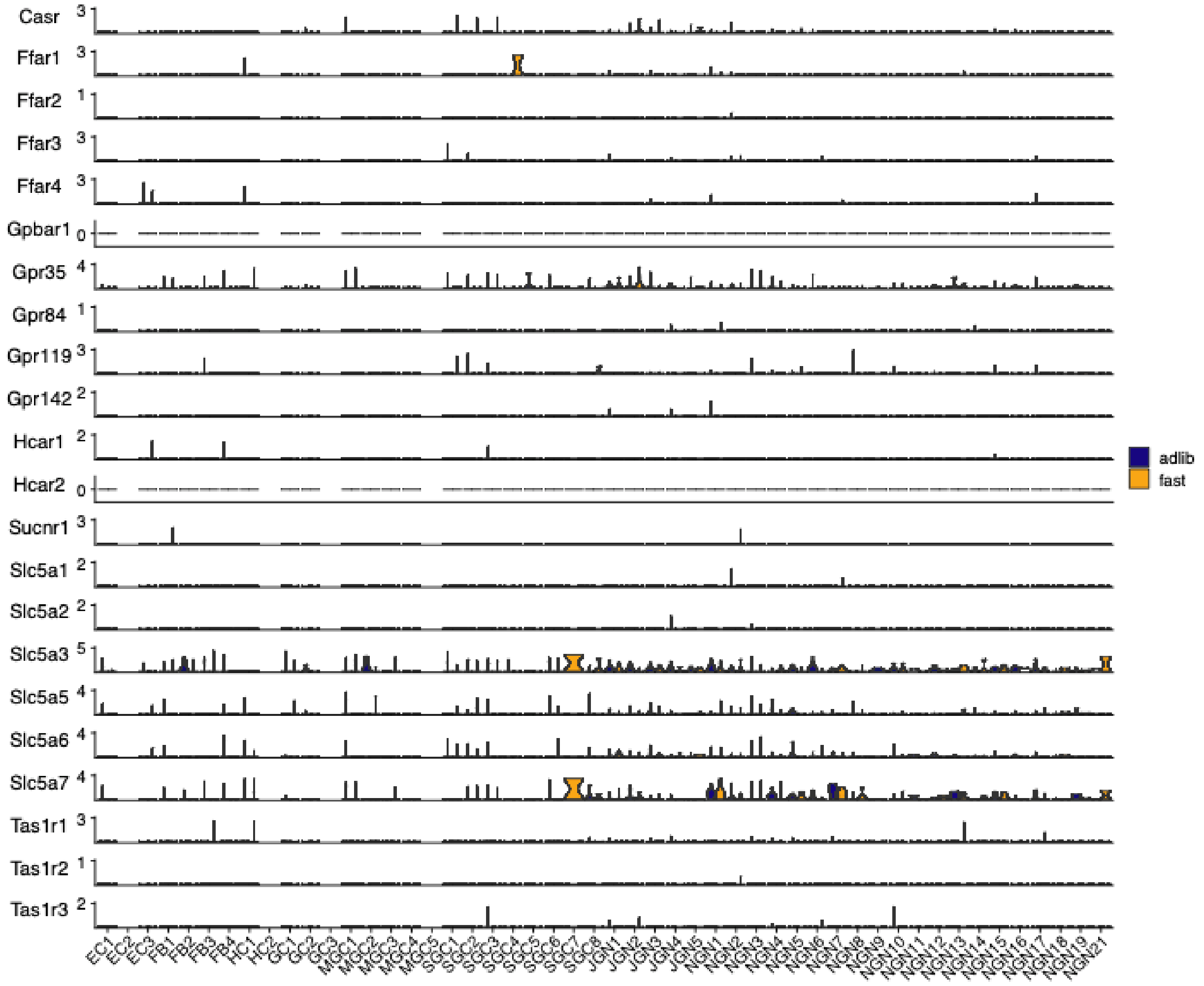

Supplementary Figure 13: Violin plot showing the expression of nutrient sensing transporters and receptors in snRNASeq clusters compared between overnight fasting and *ad libitum* feeding**.**

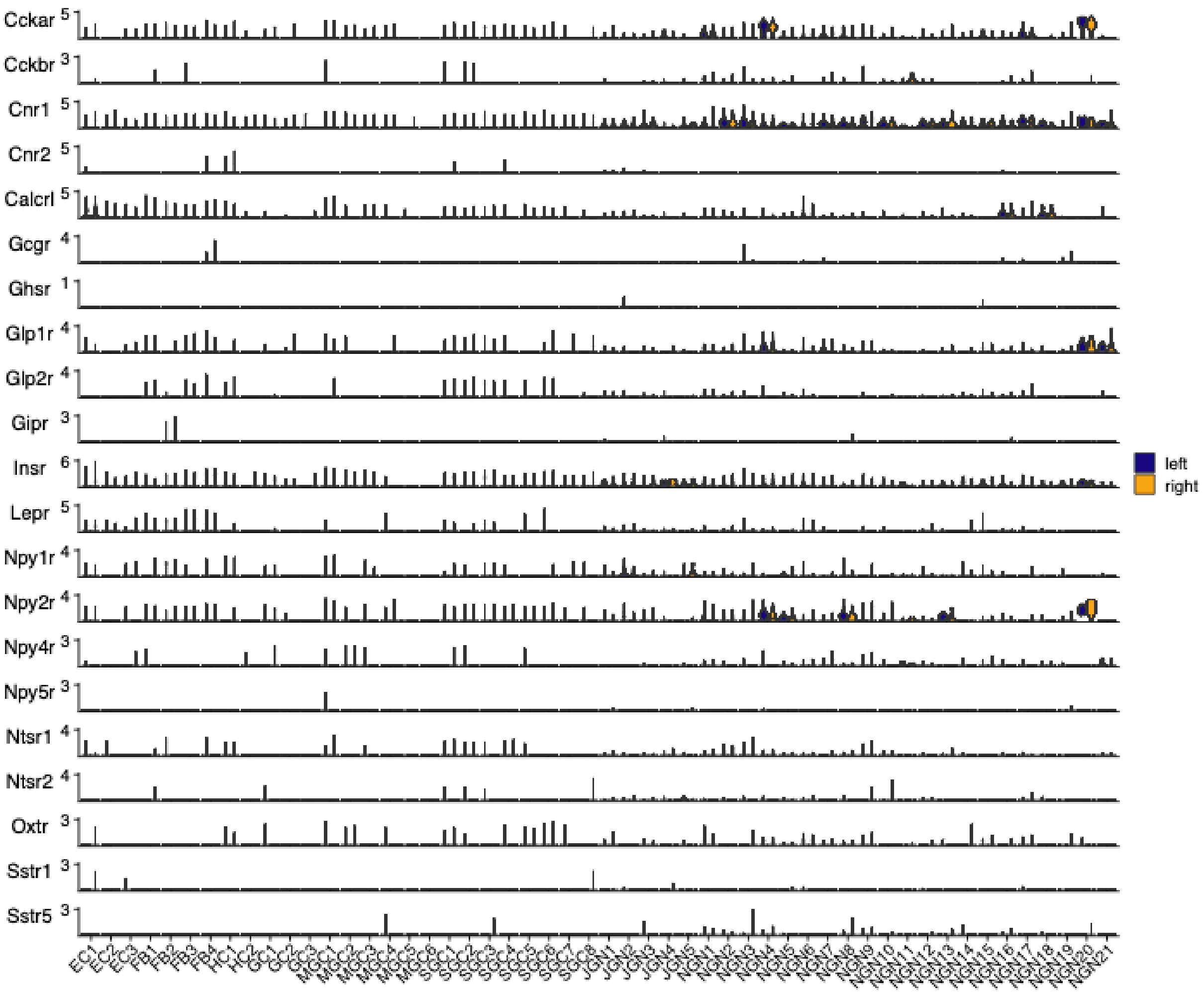

Supplementary Figure 14: Violin plot showing the expression of gastrointestinal and pancreatic hormone receptors in snRNASeq clusters compared between left and right nodose ganglia.

Supplementary Figure 15

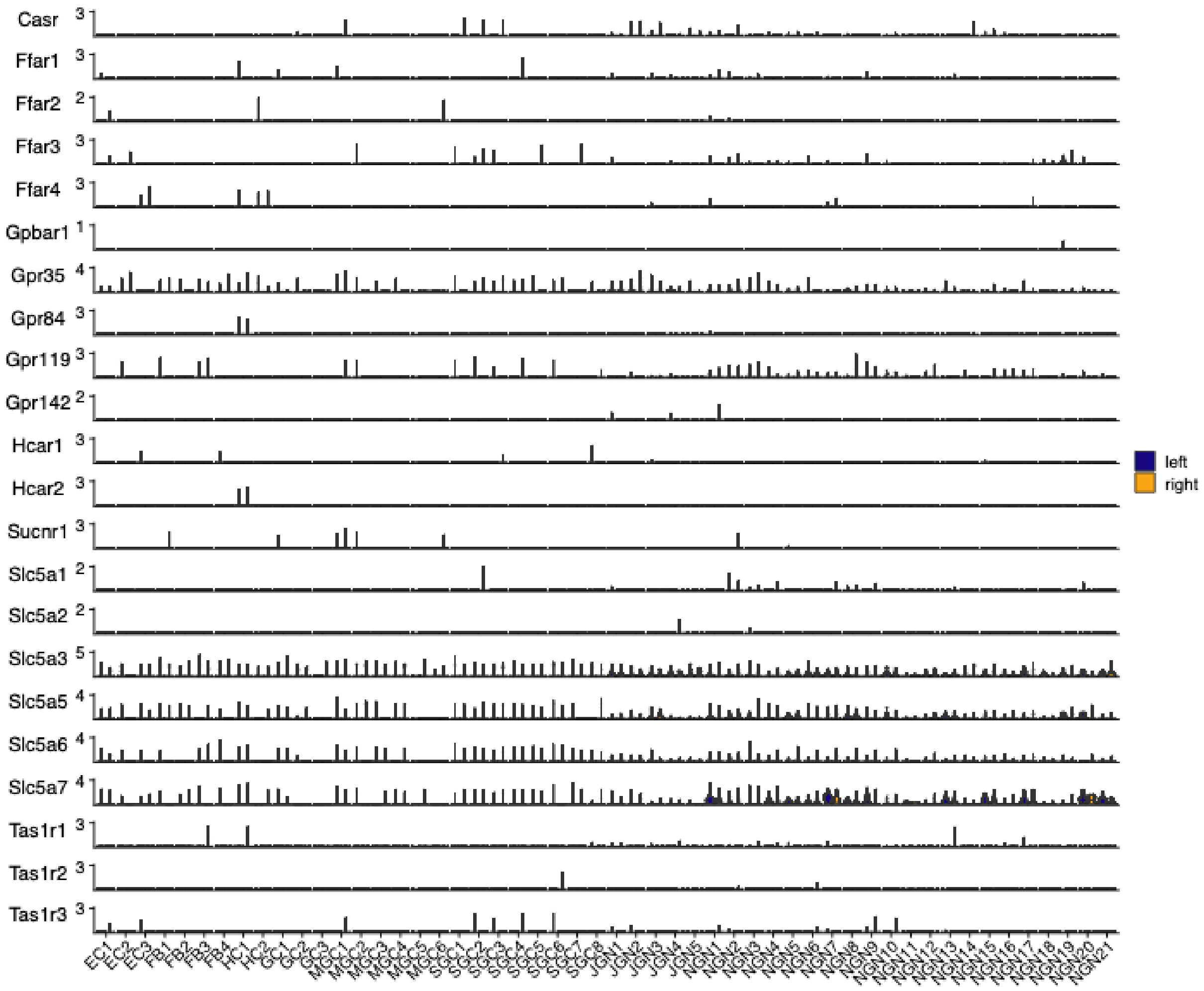

Supplementary Figure 15: Violin plot showing the expression of nutrient sensing transporters and receptors in snRNASeq clusters compared between left and right nodose ganglia.
